# Photoresponsive molecular tweezers modulate Taspase 1 activity

**DOI:** 10.1039/d5cb00069f

**Published:** 2025-09-05

**Authors:** Antonio L. Figueroa Bietti, Alisa-Maite A. Kauth, Katrin Hommel, Mike Blueggel, Laurenz Mohr, Felix C. Niemeyer, Christine Beuck, Peter Bayer, Shirley K. Knauer, Bart Jan Ravoo, Thomas Schrader

**Affiliations:** a Institute of Organic Chemistry I, Biosupramolecular Chemistry, University of Duisburg-Essen Universitätsstrasse 7 45141 Essen Germany thomas.schrader@uni-due.de; b Organic Chemistry Institute and Center for Soft Nanoscience, University of Münster Busso-Peus-Straße 10 48149 Münster Germany b.j.ravoo@uni-muenster.de; c Molecular Biology II, Center of Medical Biotechnology (ZMB) and Center for Nanointegration (CENIDE), University of Duisburg-Essen Universitätsstrasse 5 45141 Essen Germany shirley.knauer@uni-due.de; d Structural and Medicinal Biochemistry, Center of Medical Biotechnology (ZMB), University of Duisburg-Essen Universitätsstrasse 5 45141 Essen Germany

## Abstract

Light serves as an exceptional stimulus for the precise spatiotemporal regulation of protein activity and protein–protein interactions. Here, we introduce a light-responsive supramolecular ligand system designed to modulate Taspase 1, a protease critical for embryogenesis and implicated in tumor progression. Our approach utilizes photoswitchable divalent molecular tweezers engineered to target lysine-rich regions within the Taspase 1 loop. By incorporating arylazopyrazole (AAP) photoswitches, we achieve dynamic and reversible control of ligand binding. These photoswitches exhibit high photostationary states, excellent reversibility, and prolonged thermal stability of the *Z* isomer, ensuring reliable switching without photodegradation. The tweezer distance varies between *E* and *Z* isomers, enabling tunable binding interactions. Through a combination of surface plasmon resonance, enzymatic cleavage assays, and molecular dynamics simulations, we demonstrate that these ligands bind Taspase 1 with low micromolar affinity and effectively inhibit its proteolytic activity. While isomerization did not significantly affect the inhibition of protein–protein interaction, the *E*-isomers of larger tweezers exhibited powerful enzyme inhibition, likely due to their ability to bridge lysines flanking the active site. This photoswitchable tweezer system provides a versatile tool for light-controlled modulation of protein function, offering new opportunities for selectively targeting lysine-rich proteins in dynamic biological environments.

## Abstract 2

“Claws Out!”: Molecular tweezers equipped with photoswitchable linkers dynamically open and close their “claws” on irradiation. This structural transition allows precise, reversible control over proteolytic activity in a light-dependent manner.

## Graphical abstract

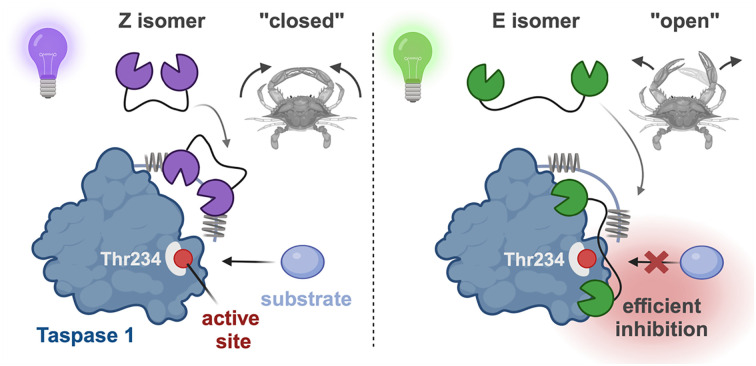

## Introduction

The ability to regulate protein function with light creates an exciting opportunity to explore fundamental biological mechanisms and to develop innovative tools for chemical biology.^1^ In addition, it bears potential for diagnostic and therapeutic applications, offering a non-invasive method that can be operated remotely through light sources.

Three main approaches have been developed to achieve photocontrol in proteins: optogenetics, photoxenoprotein engineering, and photopharmacology.^2,3^ While photopharmaceuticals have yet to achieve regulatory approval as medical drugs, light-sensitive inhibitors have been developed for numerous proteins, representing potential precursors to therapeutic applications.^4^

Supramolecular agents have already been explored as chemical tools for modulating protein function,^5–8^ as they can dock onto protein hot spots and efficiently disrupt interactions with partner proteins, as shown by us and others.^9–26^ This strategy introduces a new approach for targeting pathological protein functions, with the protein interactome - comprising over half a million biologically relevant protein–protein interactions (PPIs) - offering immense potential for therapeutic innovation. Our work is driven by the hypothesis that photopharmacology concepts can be applied to convert a rigid protein surface ligand into a dynamic, photoresponsive inhibitor of PPIs. The goal is to enable the reversible activation or inhibition of protein recognition through light irradiation. Nature provides key examples of light-controlled protein function, such as the photo-induced *cis*/*trans*-isomerization of retinal in rhodopsin, which triggers a signalling cascade allowing vertebrates to respond to light stimuli. Achieving this goal requires the development of highly efficient artificial molecular photoswitches, which can be integrated into synthetic protein ligands. These light-responsive ligands would make it possible to precisely control protein function using irradiation.^27^ Efficient photoresponsive behaviour has been achieved using synthetic photoswitches, such as azobenzenes (AZO) and diarylethenes (DAE), covalently conjugated to biomolecules like nucleic acids, peptides, and proteins.^28^*E.g.*, integrating an AZO switch into a natural peptidic binder for WDR5 enabled reversible photoswitching, which ultimately blocked leukemic cell proliferation.^29^ Similarly, “go” and “stop” peptides with incorporated photoswitches were derived from the C-terminal β-Arrestin peptide.^30^ Introducing two strategically placed cysteines into a DNA helicase enabled crosslinking with an AZO-based spacer, whose photoswitching effectively toggled protein function on and off.^31^

Photoswitchable ligands have also been engineered to bind deep clefts, such as ion channels or active sites, allowing for precise control of neuronal activity.^32^ Notably, even minor changes in binding efficacy upon photoswitching can have a profound impact on cellular output.^33^ Light-switchable DAE ligands that bind to the active site of phosphoribosyl isomerase A (PriA) *via* their terminal phosphates modulate the enzyme's catalytic activity.^34^ Similarly, photocontrol was obtained on catalysis and allosteric regulation of ImGP-S (imidazole glycerol phosphate synthase).^35^

However, to the best of our knowledge, no examples of photoswitching protein activity using supramolecular ligands targeting the protein surface have been reported. In recent years, our groups have developed unique host molecules designed to target well accessible lysine and arginine residues on protein surfaces.^9–12,36^ Known as molecular tweezers, these host molecules feature a pincer-like shape with an electron-rich cavity that selectively binds suitable cationic guest molecules through a highly selective threading mechanism.^37^ Tweezer docking to strategic lysines or arginines on a target protein modulates relevant PPIs.^11,12,36^ Multivalent tweezer assemblies are superior PPI inhibitors,^38^ and were also shown to block the tumor-relevant interaction between Taspase 1 and Importin α.^10^ Inhibitors of Taspase's catalytic activity, are discussed as promising antitumor agents. Taspase 1 is a unique threonine endopeptidase essential for embryonic development and implicated in the progression of leukemias and solid tumors.^39^ To become enzymatically active, Taspase 1 must first be transported into the nucleus (Fig. S1). This nuclear import relies on the interaction between a bipartite basic nuclear localization signal (NLS), located on two adjacent α-helices forming a flexible loop, and the transport receptor Importin α (Fig. S1A).^40^ Once inside the nucleus, Taspase 1 undergoes autoproteolysis, generating a functional heterodimer composed of an α- and a β-subunit (Fig. S1B). Previous studies have demonstrated that multivalent tweezer assemblies bind to the NLS and prevent receptor docking as well as proteolytic cleavage.^10^

**Fig. 1:**
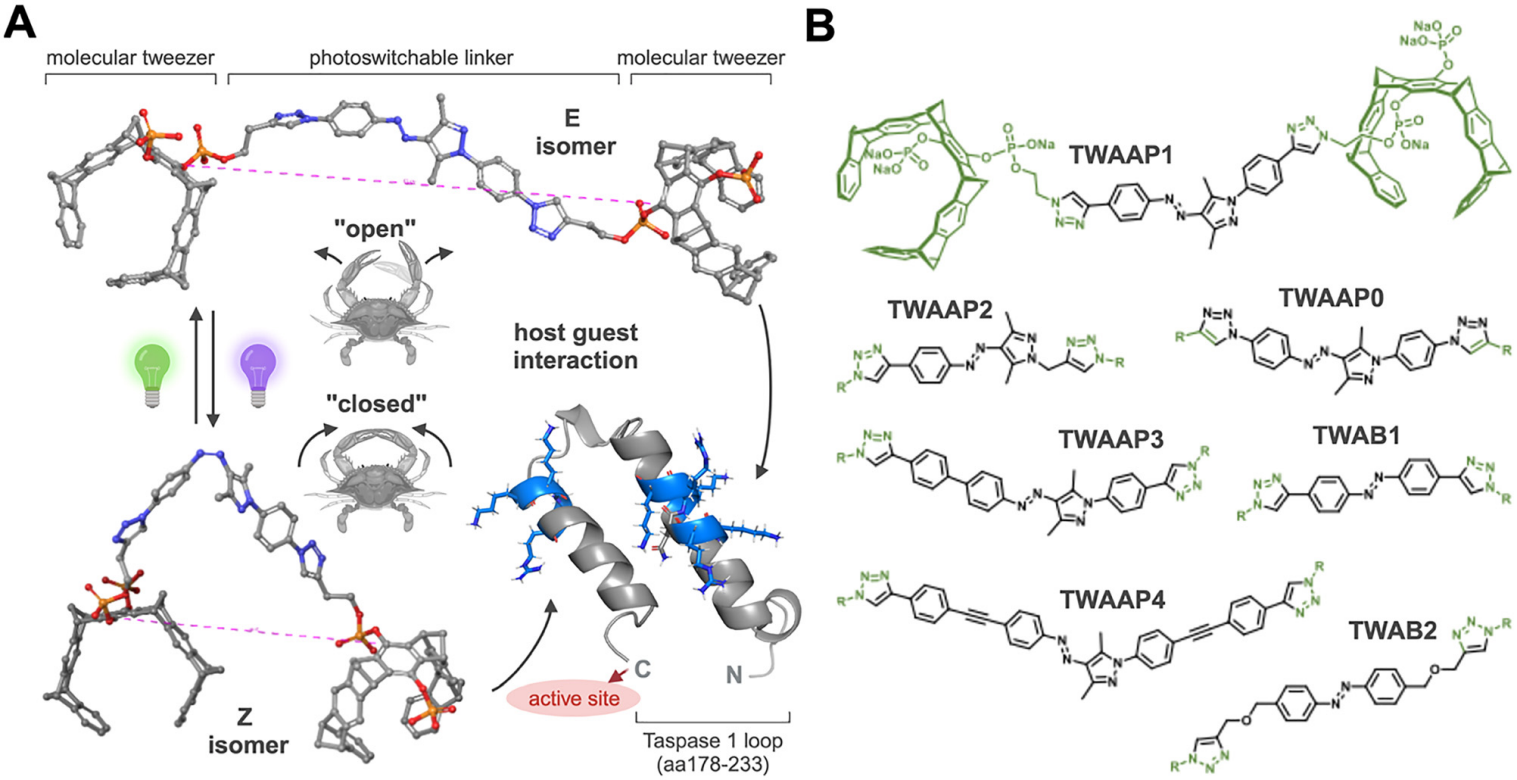
Molecular design of photoswitchable divalent molecular tweezer (TW) constructs. (A) MD energy minimized structures depicting the *E* and *Z* isomers of dimeric photoswitchable tweezer TWAAP0. Global energy minimum calculated separately for the folded (“closed”) *Z* and the extended (“open”) *E* isomer after exposure to UV (365 nm) or green light (520 nm), respectively. The distances between the tweezer units in the photoisomers are highlighted by the dotted lines. Molecular tweezer units enable the molecular recognition of lysine side chains protruding from basic amino acid clusters (blue) located on the surface loop of Taspase 1 preceding its active site (red). (B) *E* isomers of AAP photoswitches TWAAP0–4 and AZO photoswitches TWAB1–2. Composed with https://BioRender.com.

In this report, we demonstrate how to achieve light-switchable tweezer-mediated protein inhibition. To this end, we incorporate a molecular photoswitch between two tweezer moieties, connected by rigid linkers of variable lengths. We use highly efficient arylazopyrazoles (AAP) (Fig. 1), which overcome the limitations of conventional AZO photoswitches and provide near-quantitative conversion between *E* and *Z* isomers,^41–43^ a long half-life of the metastable *Z* isomer,^44^ and excellent photophysical properties in water.^45^ In recent years, the versatility of AAP has been demonstrated in various aqueous supramolecular systems, including host–guest complexes,^46,47^ hydrogels,^48^ and surfactants.^49^

Our new dimeric molecular tweezers can be effectively switched by UV light to a closed *Z* isomer and back with green light to the open *E* isomer with drastic differences in tweezer distance (Fig. 1A). In this study, we explore the photoisomerization of these dimeric tweezers and their interaction with Taspase 1. By leveraging this novel dynamic supramolecular approach, we demonstrate that protease activity can be reversibly modulated with light.

## Experimental

### Synthesis of switchable tweezer constructs

All divalent photoswitchable tweezer constructs were synthesized using an optimized click chemistry protocol. The photoswitch cores, featuring two terminal alkynes, were coupled with the monoazido tweezer, which contains a single terminal azide. The photoswitches are divided into two series, one based on the AAP unit (TWAAP0–4) and the other on the AZO unit (TWAB1–2).

### General procedure for iterative click coupling of tweezer units to diazide switches

A dry 10 mL Schlenk flask was filled under argon with 5 mg (6.28 μmol, 1.0 eq.) of the asymmetric tweezer together with the respective photoswitch (6.28 μmol, 1.0 eq.) in 3 mL dry DMF. The solution was treated with 5.7 μL (33.9 μmol, 5.4 eq.) of DIPEA and stirred for 5 min. Subsequently, 4.5 mg (31.4 μmol, 5.0 eq.) CuBr was added, and the reaction mixture was stirred for 16 h at 60 °C. The progress of the first coupling step was monitored by analytical HPLC: as soon as the peak of the asymmetric tweezer totally disappeared, the second equivalent of the asymmetric tweezer was added, followed by another 5.4 eq. DIPEA, 5 eq. CuBr and 2 mL dry DMF. The resulting mixture was stirred for another 72 h at 60 °C. The mixture was filtered over a D4 glass filter, and the solid residue was suspended in 10 mL 1 M HCl, transferred to a centrifuge vial and sonicated for 20 min. After centrifugation, the solution was decanted and the solid residue was again treated twice by sonication and centrifugation with 1 M HCl, twice with 1 mL acetone and once with 10 mL water. Finally, the resulting purified solid was transferred to a 10 mL pointed bottom flask with water and lyophilized.

### Photoisomerization

Tweezer constructs were synthesized as an *E*/*Z* mixture, with a strong preference for the thermodynamically stable *E* isomer. For photoisomerization, UV-vis spectra of TWAAP and TWAB (50 μM in water) were recorded in their initial state. The constructs were then isomerized to the *Z* isomer with UV light (*λ* = 365 nm, 1 min) and back to the *E* isomer with green light (*λ* = 520 nm, 5 min). Two light sources were used: UV LED HighPower-LED with 3 W of *λ* = 365 nm (LED Engin Inc., San Jose, California, US) and a LSC-G HighPower-LED with 3 W of *λ* = 520 nm (Cree Inc., Durham, North Carolina, US). UV-vis spectroscopy was routinely employed to analyze the tweezer constructs, serving as the method of choice to monitor configurational integrity after multiple switching cycles. More than 10 switching cycles were performed without any loss of configurational integrity. The UV-vis spectra were recorded with a JASCO V-650 double-beam spectrophotometer (JASCO).

The thermal stability of the *Z* isomers of TWAAP and TWAB (50 μM in water) was assessed using UV-vis spectroscopy at elevated temperatures due to their relatively long half-lives at room temperature. Measurements were performed in a quartz glass cuvette with an optical path length of 10 mm. Prior to each measurement, samples were irradiated with UV light for 1 min to achieve isomerization to the *Z* isomer. For TWAB, spectra were recorded at 40 °C, as higher temperatures led to degradation. For TWAAP, measurements were taken at 40 °C, 50 °C, and 60 °C at various time intervals. The absorbance at the maximum wavelength of the *E* isomer was plotted against time. The resulting data were fitted to an exponential decay model according to:
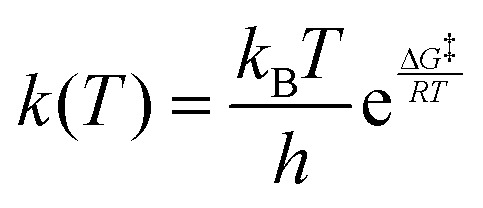


The half-life *t*_1/2_ was calculated using the following equation:
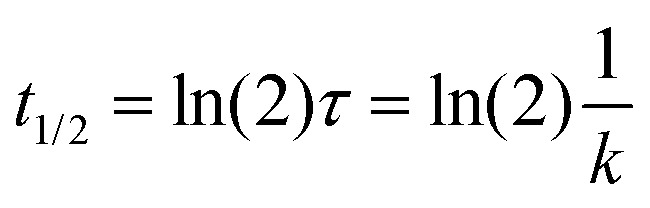
where *k* is the rate constant obtained from the exponential fit of the absorbance data over time.

To calculate the half-lives of TWAAP at 20 °C, the ln(*k*/*T*) were plotted against the inverse temperature (eyring plot) and fitted linearly:
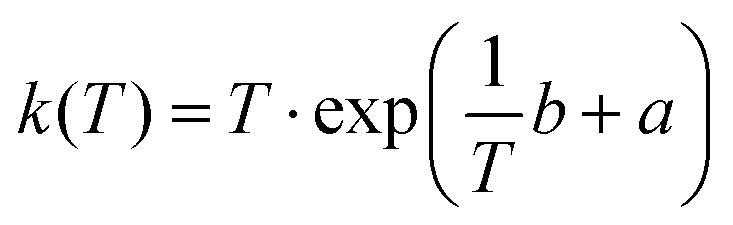


This method was used to determine *k*(20 °C) from the slope *a*, and the intercept *b* of the linear fit in the Eyring plot.

### Plasmids, expression and purification of recombinant proteins

pET22b-WT_Taspase1-His (wildtype), pET22b-Taspase1_D233A/T234A_-His (inactive), pET22b-GST-MLL_2700–2850_-GFP-His were expressed and purified as described previously.^9,10,50^ The purity of the recombinant proteins was confirmed *via* SDS polyacrylamide gel electrophoresis (PAGE) and immunoblotting.

### SDS-PAGE and immunoblotting

SDS-Page and immunoblotting was performed as described.^9,10^

### Surface plasmon resonance (SPR)

SPR measurements were conducted as described.^9,10^

### Pull-down assay

Pull-down assay: recombinant pull-down assays were performed as described.^9,10^ For details on the assay workflow, see SI (Fig. S6).

### Fluorescence assays

Fluorescence titration was conducted with a Taspase 1 loop with an N-terminal 6-Carboxyfluorescein (Cy5)-label synthesized by GeneCust (Sequence: Cy5-SCPPNIMTTRFSLAAF **KRNKRK**LELAERVDTDFMQL**KKRR**QSSEKENDSGTLD; NLS in bold) as described.^9,10^ The fluorescence anisotropy assay was modified based on previously established protocols.^51^ Measurements were conducted using an FP-8300 JASCO fluorescence spectrometer (JASCO GmbH). The Importin α:Cy5-labeled Taspase 1 loop complex was analyzed in 1× PBS (pH 7.4) at 25 °C, with Importin α titrated stepwise into a solution containing 50 nM Cy5-Taspase 1 loop. To evaluate the impact on binding, the Cy5-loop and the Importin α:Cy5-labeled loop stock solution were incubated with 20 μM of each TWAAP ligand. The latter ensures to avoid dilution effects during Importin α titration. Binding curves of the average data were fitted with GraphPad Prism 10.4 (GraphPad) using the quadratic binding equation for one-site specific binding model:
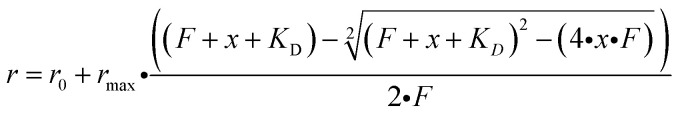
with *r* = anisotropy, *r*_0_ = anisotropy without protein, *r*_max_ = maximum anisotropy, *F* = fluorescent probe (Cy5-labeled peptide) concentration, *x* = titrant concentration, *K*_D_ = dissociation constant. All fitting curves have *R*^2^-values between 0.88 and 0.98.

### Protein NMR spectroscopy

NMR spectroscopy was performed as described.^9,10^

### Recombinant cleavage assay

The recombinant cleavage assay employing the recombinant fusion protein GST-MLL_2700–2850_-GFP-His, which contains the CS2 cleavage site of the Mixed-lineage leukemia (MLL) protein as a substrate for Taspase 1, was performed as described.^9,10^

### Software

In UV-vis spectroscopy, Spectra Manager 2.08.04 (JASCO) was used for data acquisition, and OriginPro 2024 (Originlab) was used for data analysis. Western blot and Coomassie gel images were captured using the Chemidoc Imaging System (BioRad) and quantified through densitometric analysis using Fiji.^52^ Fluorescence titration and fluorescence polarization data were acquired using Spectra Manager™ II (Jasco) or GloMax (Promega) software provided with the respective instruments. Data were analyzed using Graph Pad Prism version 9. SPR data were processed with TraceDrawer 1.10.1, employing a 1 : 1 binding model for fitting. NMR data were collected using Topspin 3.5 (Bruker) using the NMRlib 2.0 pulse sequence tools library from IBS (Grenoble, France) available at https://www.ibs.fr/research/scientific-output/software/pulse-sequence-tools/. NMR spectra were processed with Topspin 3.5 (Bruker) and analyzed in CARA 1.9.1.7 (https://cara.nmr.ch). Relative signal intensities were calculated from the raw chemical shift data and peak intensities using Excel 2016 (Microsoft) and plotted with GraphPad Prism 5.0. All molecular modelling and dynamics calculations were performed in Maestro 13. 0 & 13.6, the corresponding images generated with PyMOL. https://BioRender.com was used for the design of schematic illustrations and for assembly of multi-part composite figures.

### Protein modeling, tweezer placement, and MD simulations

The structure used for simulations was based on a model of Taspase 1 with a cleaved and opened C-terminal loop of the α-subunit, as described.^50^ Initial protein preparation was carried out using the Protein Preparation Wizard in Schrödinger's suite, applying the following steps: bond orders were assigned with reference to the CCD database, hydrogens were added, and missing side chain atoms were completed. All crystallographic water molecules were removed, and heteroatom states were generated at pH 7.3. Protonation states were refined using Epik for pKa prediction at pH 7.4, and hydrogen bond assignments were optimized using PROPKA at the same pH. A restrained energy minimization was subsequently performed to relax the structure, converging heavy atoms to an RMSD of less than 0.3 Å. To investigate active-site accessibility, synthetic molecular tweezers were manually docked onto K57 and R262, which flank the catalytic site. The tweezers were functionalized with TWAAP linkers at the phosphate moieties, enabling the large construct to span the active-site cleft and sterically hinder substrate approach to the nucleophilic threonine (Thr234). All-atom molecular dynamics (MD) simulations were conducted using Desmond (D. E. Shaw Research). The system was solvated in an orthorhombic SPC water box with a 10 Å buffer surrounding the protein complex. The system was neutralized with sodium ions and brought to physiological ionic strength with 150 mM NaCl. Simulations were run under NPT ensemble conditions at 300 K and 1 atm (1013 mbar) for 200 ns, with trajectory frames saved every 1.2 ps, yielding 1000 frames for analysis. All simulations were executed using a NVIDIA GTX 1070 GPU.

## Results and discussion

### Molecular design and synthesis of photoswitchable tweezer constructs

The rigid and efficient synthetic connection of recognition elements at both ends of a photoswitchable linker moiety was achieved through an optimized click protocol between two azido tweezers and one dialkyne photoswitch (Scheme S1). The central AAP unit was extended with additional 1,4-substituted benzene and ethyne fragments to maintain rigidity in the photoswitchable tweezers TWAAP0-4 (Fig. 1B).^53^ In addition, we included two photoswitchable tweezers TWAB1-2 with an AZO unit in our study. This design positions the tweezer units at the end of a “folding knife”-like backbone, maximizing the spacing difference between the tweezer units in the photoisomers (Fig. 1A). Molecular dynamics (MD) simulations predict a distance range of 22–38 Å for the *E* isomers and of 13–20 Å for the *Z* isomers (Fig. S2a–g and Table 1). Shorter distances were observed with similar AZO constructs, allowing us to compare the performance of the AAP and AZO photoswitches and to establish a preliminary structure–activity relationship (SAR).

**Table 1:** Distances between the outer points of the tweezer units in the *E* (Δ_*E*_) and the *Z* isomer (Δ_*Z*_) at a local energy minimum, as well as the maximum (Δ_max_) and minimum (Δ_min_) possible distance between the tweezer units

TW construct	Δ_*E*_ [Å]	Δ_*Z*_ [Å]	Δ_max_ [Å]	Δ_min_ [Å]
TWAAP0	30	18	33	9
TWAAP1	30	20	33	10
TWAAP2	22	13	29	8
TWAAP3	32	15	36	8
TWAAP4	38	13	47	9
TWAB1	28	16	31	10
TWAB2	17	12	36	10

To prevent unwanted aggregation and precipitation of the monovalent intermediate resulting from incomplete reaction, the CuAAC reaction was carried out in the solvent DMF at 40 °C in an iterative one-pot process.^53^ Following careful neutralization with aqueous NaOH, dianionic products with moderate to good water solubility in the micromolar range were obtained. The absence of significant upfield shifts indicated no inclusion of the extended arms inside the cavities, likely due to the mutual repulsion of their phosphates (Fig. S3).

### Photophysical properties of the tweezer constructs

**Fig. 2:**
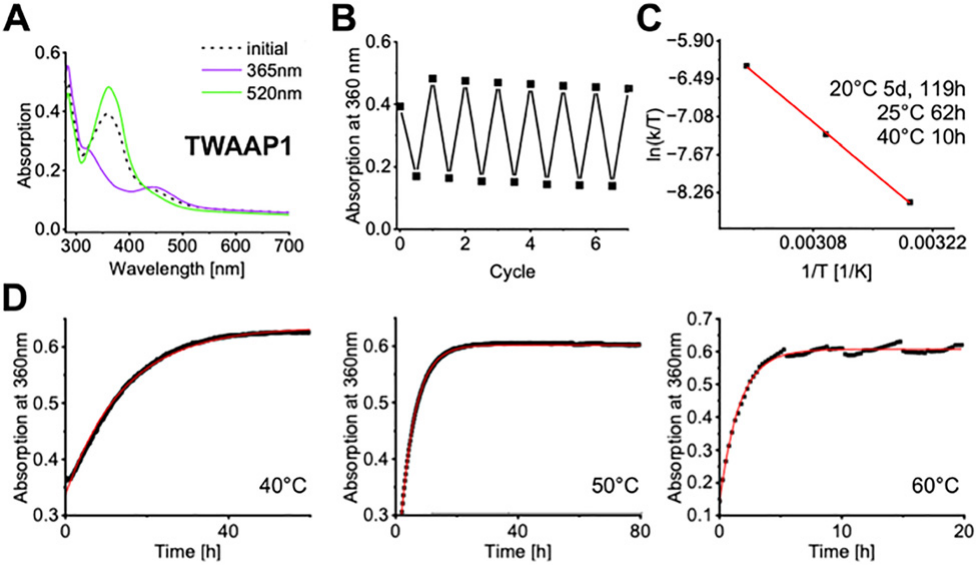
Photophysical properties of TW constructs. (A) Absorption spectra of the divalent tweezer TWAAP1 before (dashed line) and after irradiation with UV light (365 nm, 1 min, violet) or green light (520 nm, 5 min, green). (B) Photoisomerization of TWAAP1 over 7 cycles. (C). *E*yring plot for the thermal *Z*-to-*E* relaxation of TWAAP1 (*c* = 50 μM in H_2_O).

The photophysical properties of AAP tweezers TWAAP0-4 as well as those of AZO tweezers TWAB1-2 were consistent with previous reports on these photoswitches. The photophysical properties of AAP tweezers TWAAP0-4 as well as those of AZO tweezers TWAB1-2 were consistent with previous reports on these photoswitches. Irradiation with green light (520 nm, 5 min) led to a photostationary state (PSS) with a high concentration of *E* isomer, while UV irradiation (365 nm, 1 min) resulted in a PSS with a high concentration of the *Z* isomer. The two isomers are easily distinguished by the significant difference in their absorption bands (Fig. 2 and Fig. S4a, b). Additionally, the ^1^H NMR spectrum of the aromatic region around the AAP and the AZO moiety, respectively, displays a distinct splitting pattern, allowing for the determination of the *E*/*Z* ratio in the PSS (Fig. S5 and Table S1). This analysis revealed that the click reaction produces mixture of *E*/*Z* isomers, with a strong preference for the thermodynamically more stable *E* isomer. Irradiation with green light further increases the concentration of the *E* isomer, as confirmed by both UV/vis and NMR spectra. The switching process can be repeated for over 10 cycles without loss of configurational integrity or photodegradation of the tweezer. Thermal relaxation experiments of the *E*/*Z* isomers at increasing temperatures showed robust stability, with half-lives of several days at room temperature. (Fig. 2 and Table S1).

### Binding of photoswitchable tweezer constructs to Taspase 1

**Fig. 3:**
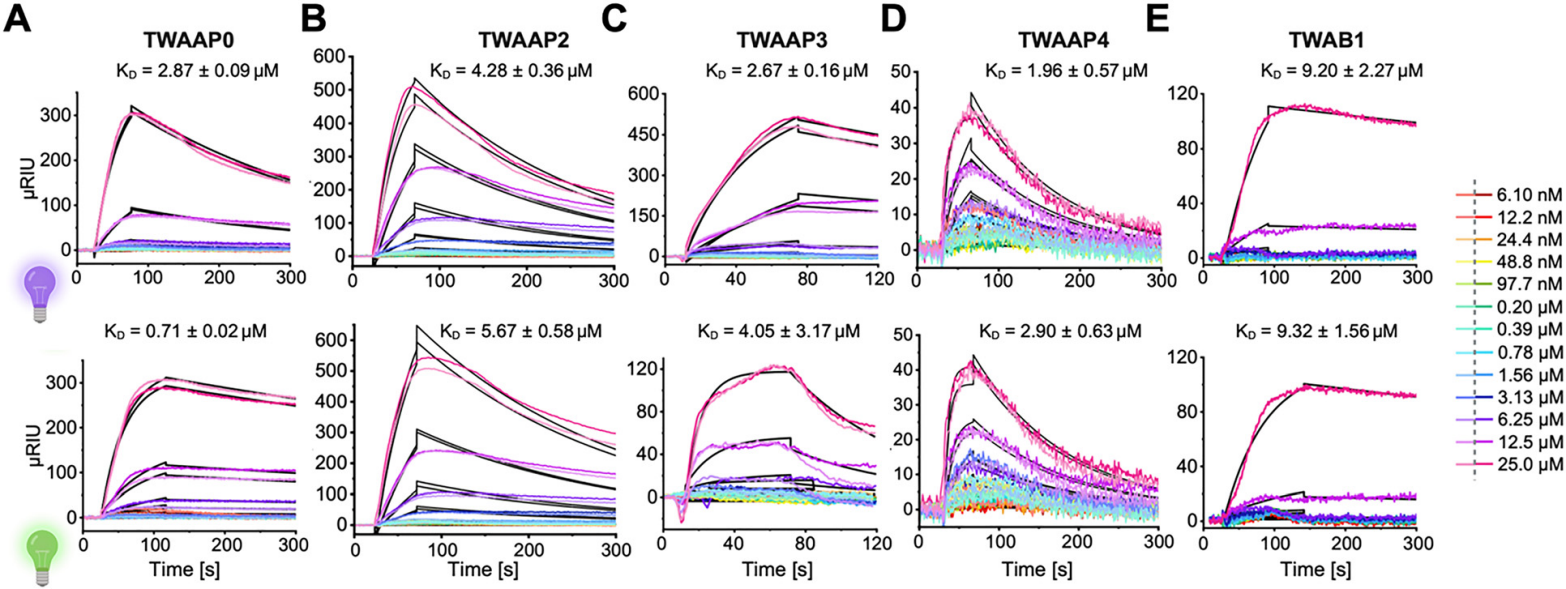
Photoswitchable tweezer constructs bind Taspase 1 with SPR binding affinities in the low micromolar range. Sensorgrams depict the binding interactions between TWAAP0-4, and TWAB1 with immobilized full-length, proteolytically inactive Taspase 1, as monitored by SPR. Duplicate measurements were performed, with the respective curves shown in the same colour at different brightness levels (right). As indicated, tweezer concentrations from bottom to top were 25, 12.5, 6.25, 3.13, 1.56, 0.78, 0.39, 0.20 μM, and 97.7, 48.8, 24.4, 12.2, and 6.10 nM. *K*_D_ values (Table S2) were determined through kinetic evaluation by fitting the individual curves to a 1 : 1 binding model. Detailed SPR data are provided in the SI (Table S3). Composed with https://BioRender.com.

First, we utilized an established surface plasmon resonance (SPR) assay to evaluate the overall binding of our ligands to Taspase 1 (Fig. 3).^10,54^ Notably, since wild-type Taspase 1 undergoes partial autoproteolysis over time, we employed a well-characterized catalytically inactive mutant in selected assay setups, such as SPR and other biochemical assays (see below), to prevent potential artifacts from molecular weight shifts due to autocleavage. Thus, full-length, inactive Taspase 1 protein was immobilized on a dextran-coated gold chip and titrated with solutions of a divalent *E*- or *Z*-enriched tweezers. High affinities were observed, with *K*_D_ values in the single micromolar range (Fig. 3, and Table S2). For instance, titration of TWAAP0 to immobilized Taspase 1 produced signals of approximately 300 μRIU for both *E* and *Z* isomers, with comparable association rate constants (*k*_a_) of 1.04 × 10^3^ M^1^ s^1^ for the *E* isomer and 1.12 × 10^3^ M^1^ s^1^ for the *Z* isomer (Table S3). Notably, the *E* and *Z* isomers exhibited different dissociation rates (*k*_d_). The *Z* isomer had a significantly higher dissociation rate constant (*k*_d_ = 3.22 × 10^−3^ s^−1^) compared to the *E* isomer (*k*_d_ = 7.43 × 10^−4^ s^−1^).

These data indicate that the *Z*-TWAAP0 molecule detaches more rapidly from Taspase 1 than the *E*-TWAAP0 molecule, resulting in a higher dissociation equilibrium constant (*K*_D_). Indeed, the *E* isomer forms a more stable complex, as evidenced by its *K*_D_ being approximately four times lower than that of the *Z* isomer (Fig. 3, and Table S2). In the case of TWAAP3, the signal for the *E* isomer is approximately 120 μRIU, while the *Z* isomer exhibits a markedly higher signal of about 500 μRIU. Additionally, the difference between the *E* and *Z* isomers is more pronounced in the *k*_*a*_ than in the *k*_d_. For *E*-TWAAP3, the association rate constant *k*_a_ = 3.81 × 10^3^ M^1^ s^1^, whereas *Z*-TWAAP3 shows much lower *k*_a_ = 9.57 × 10^2^ M^1^ s^1^ (Table S3). This distinction is also evident in the sensorgrams. Similarly, the ligand-analyte complex formed by the *E* isomer, which has a dissociation rate constant (*k*_d_) of 1.53 × 10^−2^ s^1^, dissolves at a faster rate than the *Z* isomer (*k*_d_ = 2.56 × 10^−3^ s^1^). This indicates that the complex formed by *E* isomer is generated at a significantly faster rate but is less stable compared to the complex formed by the *Z* isomer. For TWAAP3, the *K*_D_ of the *E* and *Z* isomers differ by a factor of 1.5, with the *Z* isomer forming the more stable complex.

We note that in contrast, TWAB1 binds with one order of magnitude lower affinity. Furthermore, TWAB1 shows only a small difference in the *k*_a_ values between the *E*- and *Z* isomers (Table S3), resulting in a negligible difference in the *K*_D_ (Table S2). Similarly, the ligand-analyte complex formed by the *E* isomer, which has a dissociation constant (*K*_D_) of 1.53 × 10^−2^ s^1^, dissolves at a faster rate than that of the *Z* isomer (*K*_D_ = 2.56 × 10^−3^ s^1^). This indicates that the complex formed by *E* isomer is generated at a significantly faster rate but is less stable compared to the complex formed by the *Z* isomer. In our study, Taspase 1 was immobilized without a specific tag using a carboxylated C1 sensor chip with a matrix-free surface for covalent attachment. Consequently, the chip likely contained a heterogeneous population of molecules in various orientations, with most retaining their functional integrity and mimicking the behavior of the soluble protein. While coupling events near the loop region could theoretically affect ligand recognition or proteolytic activity, our experiments demonstrated reversible and reproducible ligand binding, indicating a stable system.

### Interference of photoswitchable tweezer constructs with the Taspase 1/importin α interaction

A customized biochemical pull-down assay (Fig. S6A) was employed to assess the effects of our photoswitchable tweezer constructs on the Taspase 1/Importin α interaction.^10^ GST-coupled Importin α was immobilized on Sepharose and treated with an incubated Taspase 1-tweezer mixture containing either the *E* or *Z* isomer.

**Fig. 4:**
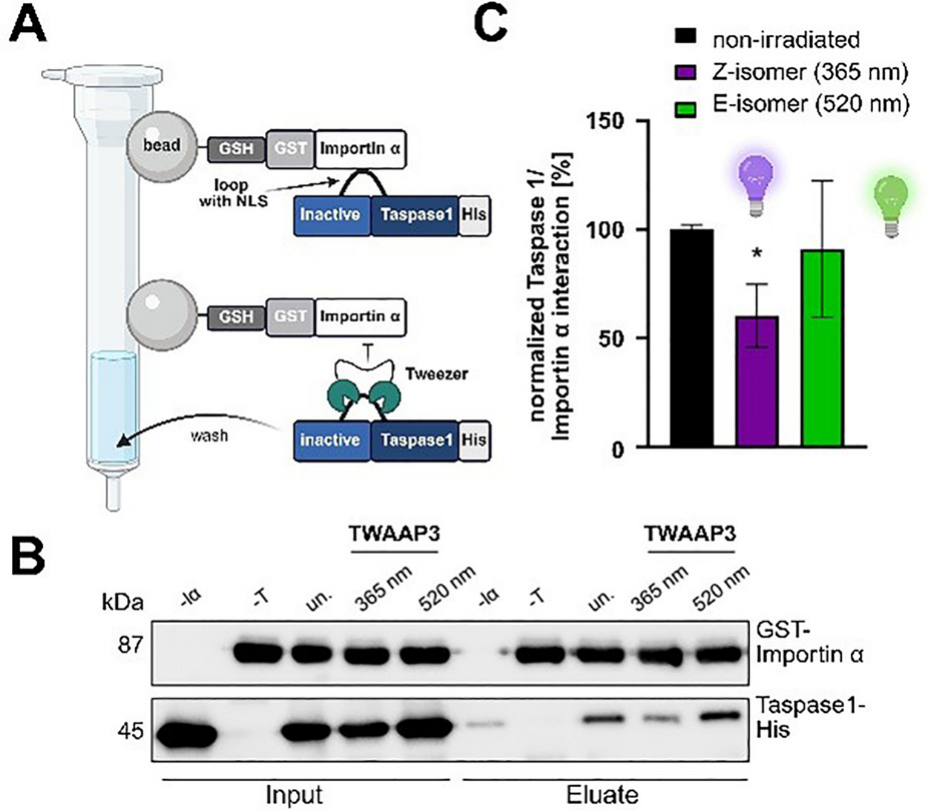
TWAAP3 disrupts the Taspase 1/Importin α interaction in its *E* isomeric form. Pull-down assays were conducted with 100 μM of TWAAP3 in either its *Z* or *E* isomer form or left untreated (un.), omitting the ligand. GST-Importin α (2.5 μM) was immobilized on the GSH-coated column as bait, while 2.2 μM inactive Taspase 1-His was used as prey. To achieve the respective *Z* or *E* isomer, TWAAP3 was irradiated with UV light (365 nm) or green light (520 nm) prior to incubation. All samples contained 3% DMSO during ligand incubation. Negative controls included samples lacking either GST-Importin α (-Iα) or Taspase 1 (-T). *, *p* < 0.05, *t*-test. Composed with https://BioRender.com.

However, apart from TWAAP3, no difference between the *E*/*Z* isomers was detected (Fig. 4). Here, only the folded *Z* isomer interfered with the PPI, but not the extended configuration. Moreover, control experiments omitting GST-Importin α revealed nonspecific binding of all TWAAP tweezers - except TWAAP3 - to the GSH beads (Fig. S6B–D). This was further supported by the persistent yellow coloration of the column material resulting from free tweezers, suggesting nonspecific attraction (Fig. S6D). Consequently, this assay setup does not provide a reliable means to comparatively evaluate the tweezer constructs' potential to disrupt this critical PPI. A possible explanation for this observation is discussed in the modeling section (*vide infra*). Additionally, due to severe protein aggregation under the respective buffer conditions, both TWAB constructs were unsuitable for this assay.

### Direct binding of photoswitchable tweezer constructs to the Taspase 1 loop

**Fig. 5:**
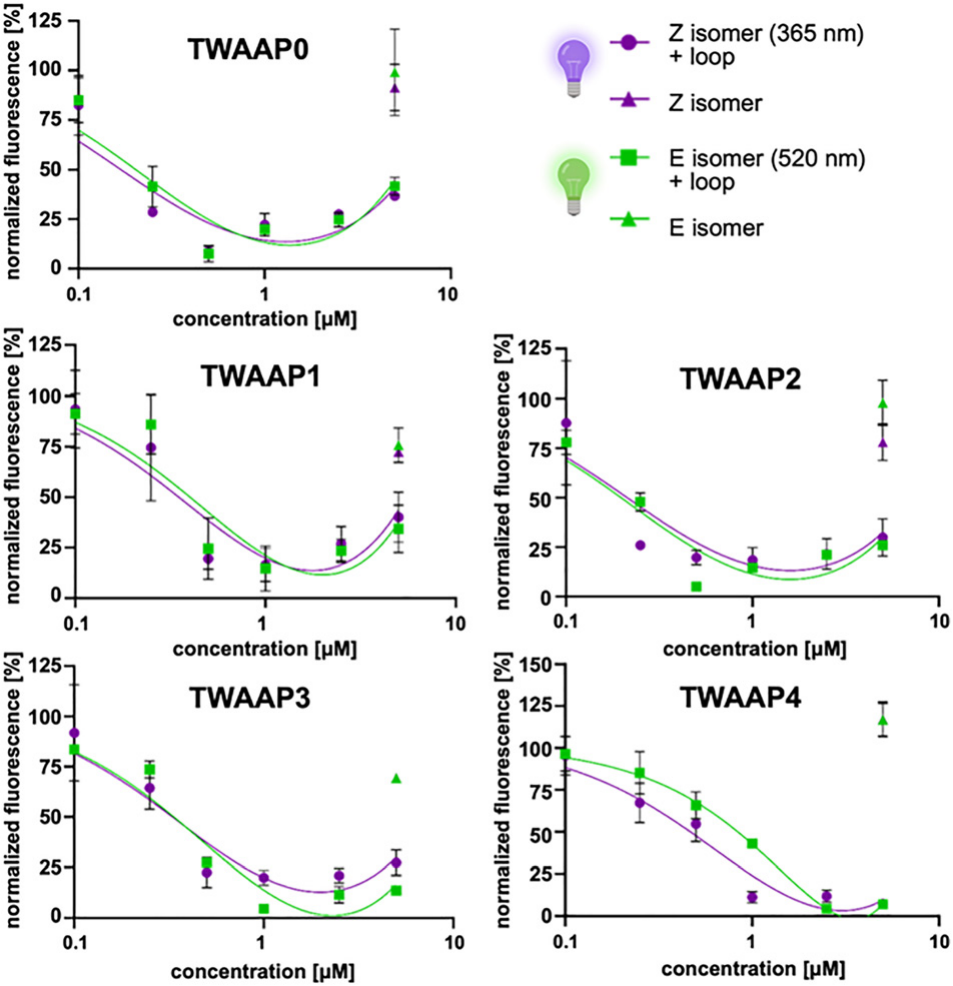
TWAAP ligands directly address the Taspase 1 loop. Fluorescence titrations were performed using a Cy5-labeled Taspase 1 loop and switchable tweezers. A 1 μM solution of the Cy5-labeled loop was incubated with increasing concentrations (0.1–5 μM) of TWAAP0-4, and fluorescence was measured at *λ*_ex_ = 645 nm/*λ*_em_ = 665 nm. Fluorescence intensity in solvent-treated samples was set to 100%, with ligand-treated samples normalized to this value. A decrease in normalized fluorescence intensity was observed with increasing ligand concentrations, indicating quenching due to ligand binding to the loop peptide. As a control, all ligands were added to Cy5 (without peptide) at their highest concentration, resulting in neglectable changes in fluorescence. Data represent the mean ± SD of three independent experiments. Composed with https://Biorender.com.

Since Importin binding critically depends on the basic NLS clusters within the Taspase 1 loop, pull-down assays typically provide a reliable readout for tweezer interaction with the relevant lysine and arginine residues. Alternatively, loop binding can be evaluated through ligand titration experiments using a fluorescently labeled Taspase 1 loop (Fig. 5).^10^ Indeed, fluorescence anisotropy measurements using a Cy5-labeled NLS-bearing loop peptide (aa S181-D233) and increasing concentrations of recombinant Importin α revealed a dissociation constant of 64 ± 20 nM indicative of a high-affinity interaction (Fig. S7A). Fluorescence titrations using the same Taspase 1 loop and the photoswitchable tweezers TWAAP0-4 revealed exceptionally strong binding of the ditopic ligands, with dissociation constants (*K*_D_) in the nanomolar range, reaching as low as 200 nM (see Table S4). This binding affinity is significantly stronger than previously reported for multivalent AIE tweezers.^9^ This assay demonstrated high robustness and reliability, with minimal variation, and no binding was observed for the fluorophore alone (Fig. S7B). Notably, while we characterized our photoswitchable TWAAP constructs as high-affinity loop binders, the differences between the *E* and *Z* isomers were absent or minimal, as for TWAAP4. However, unlike the pull-down assay, which revealed differential behavior of the TWAAP3 isomers, this experiment focuses solely on binding to a small segment of the Taspase 1 protein. As a result, potential effects related to the orientation of the flexible loop within the context of the globular protein are not captured. Of note, the slight increase in signal at the highest compound concentration likely reflects non-specific effects such as weak autofluorescence, inner filter artifacts, or molecular crowding, highlighting limitations of the assay at this range. Despite these effects, the data points were retained to provide a complete representation of the assay response.

To verify the competitive binding of our photoswitchable tweezers and Importin α to the loop in solution, we again employed fluorescence anisotropy measurements (see Fig. S7A),^10^ this time in a competitive setting.^55^ The loop was thus incubated with 20 μM of the irradiated tweezer constructs before competition with unlabelled Importin α (Fig. S7C). Unlike the characteristic exponential binding curve of Importin α, which eventually reached saturation (Fig. S7A), the presence of the tweezer constructs significantly impaired binding, shifting the dissociation constants from the low nanomolar range to the high nanomolar (TWAAP1) or even low micromolar range (TWAAP2-4). This not only confirms direct loop binding but also demonstrates strong interference with Importin α binding. Notably, previous pull-down assays could confirm this effect only for TWAAP3 (Fig. 4) in its *Z* isomeric form, as all other constructs exhibited nonspecific Sepharose bead binding.

Except for TWAAP2, the measured *E* and *Z* isomers exhibited a 2- to 4-fold difference in binding, showing a perfectly inverse correlation with the data from SPR measurements and fluorescence titrations (see also Table S4). To exclude the possibility that Importin α binds non-specifically to the Cy5 label rather than the loop, we conducted a titration experiment using increasing concentrations of Importin α (0–2000 nM) against 50 nM Cy5 without the coupled peptide as a negative control (Fig. S7B).

It should be noted that due to the very high affinity of the Taspase 1-Importin α interaction (Fig. S7A), attempts to perform reverse competition experiments starting from a pre-formed complex and displacing Importin α with the tweezers were not feasible. The molecular tweezers, exhibiting lower affinity, are unable to disrupt an already established Importin-Taspase 1 complex under the conditions tested. Consequently, the determination of IC_50_ and *K*_*i*_ values based on such displacement assays is not applicable here. Furthermore, while *R*^2^ values provide an indication of the goodness-of-fit for the binding curves, they are not direct measures of the precision or confidence intervals of the derived binding constants. In cases where binding saturation is incomplete or data cover only partial binding isotherms, such as for some TWAAP titrations, high *R*^2^ values may coexist with large standard deviations of fitted parameters. For consistency and comparability, we limited ligand concentrations to ranges compatible with all tweezers tested.

**Fig. 6:**
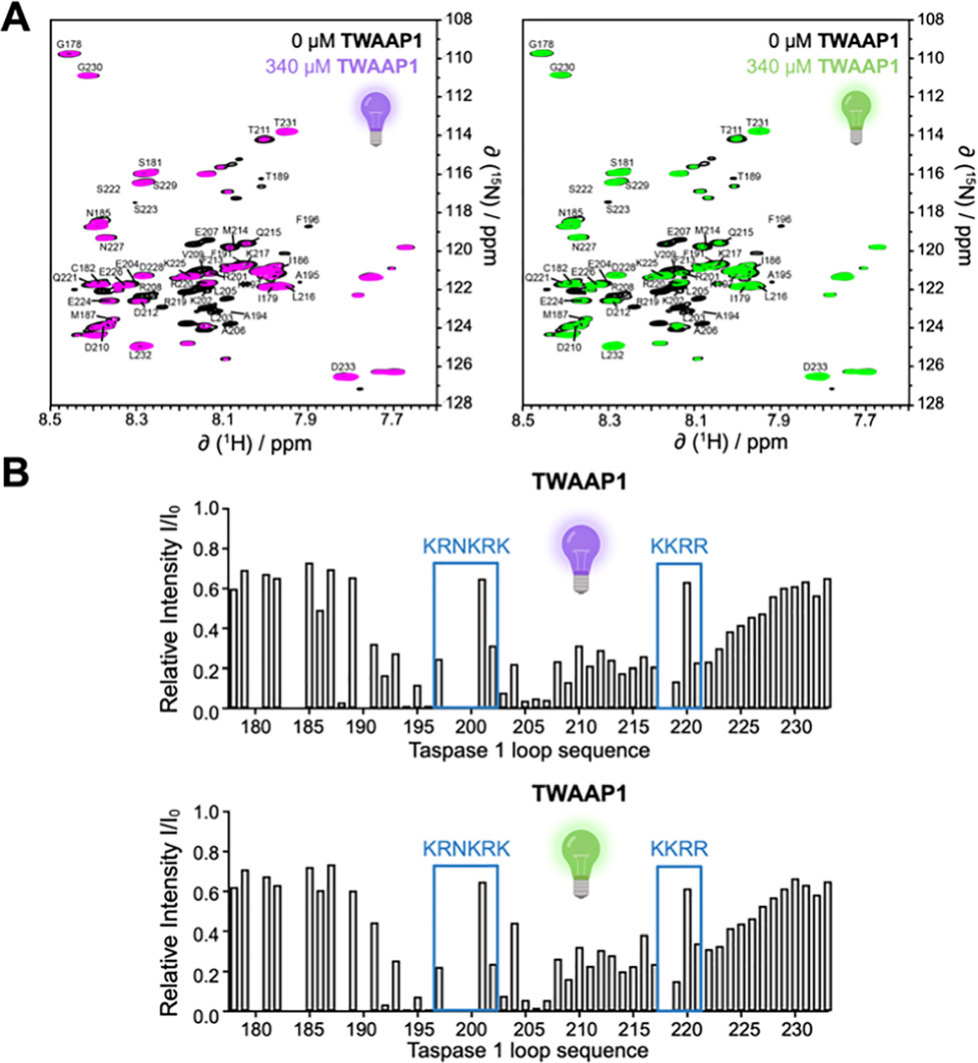
^1^H,^15^N-HSQC titrations of the ^15^N-Taspase loop with TWAAP1. A. 2D ^1^H-^15^N-HSQC spectra of the Taspase 1 loop after addition of *Z*-TWAAP1 (left) or *E*-TWAAP1 (right). Black: Taspase 1 loop without tweezers; magenta/green: with 1 equivalent of the indicated TWAAP. B. Changes in NMR signal intensities within the Taspase 1 loop178–233 with its two NLS regions (blue boxes) after complex formation with photoswitchable tweezer TWAAP1 in the *Z* (left) or the *E* (right) isomeric state in PBS buffer (pH 6.5/25 °C). Composed with https://BioRender.com.

Next, we conducted an exemplary 2D NMR experiment with the *E* and the *Z* isomers of TWAAP1 to map their contact regions on the Taspase 1 loop (Fig. 6). In this experiment, up to equimolar amounts of TWAAP1 were added to the ^15^N-labeled loop, for which a complete resonance assignment is available.^10^ The observed reduction in signal intensities for residues involved in binding is consistent with line broadening due to intermediate-exchange kinetics.^56^ Of note, chemical shift perturbations were not analyzed, as signal disappearance prevented reliable tracking of peak positions. The ^15^N-HSQC spectra of the resulting 1 : 1 complexes, compared to the unbound Taspase 1 loop (Fig. 6A), revealed a distinct reduction in relative signal intensities, particularly in regions containing the bipartite NLS with its two basic clusters, _197_KRNKRK_202_ and _217_KKRR_220_ (Fig. 6B). This effect was most pronounced for lysine residues, as those can be directly included in the tweezers’ cavities. Although this experiment confirmed the high-affinity binding of our photoswitchable tweezers to the loop, no significant differences were observed between the *E* and the *Z* isomer. This lack of distinction may be attributed to the high concentrations used and the intrinsic flexibility of the isolated loop structure.

Taken together, our experiments robustly demonstrate the direct interaction of our photoswitchable tweezer constructs with the helical NLS loop regions which contain accessible basic amino acids, where they interfere with Importin α binding - a prerequisite for proteolytic activation of Taspase 1. However, this PPI inhibition seems to be equally strong for *Z*- and *E*-isomer.

### Photoswitchable tweezer constructs inhibit Taspase 1 proteolytic activity

**Fig. 7:**
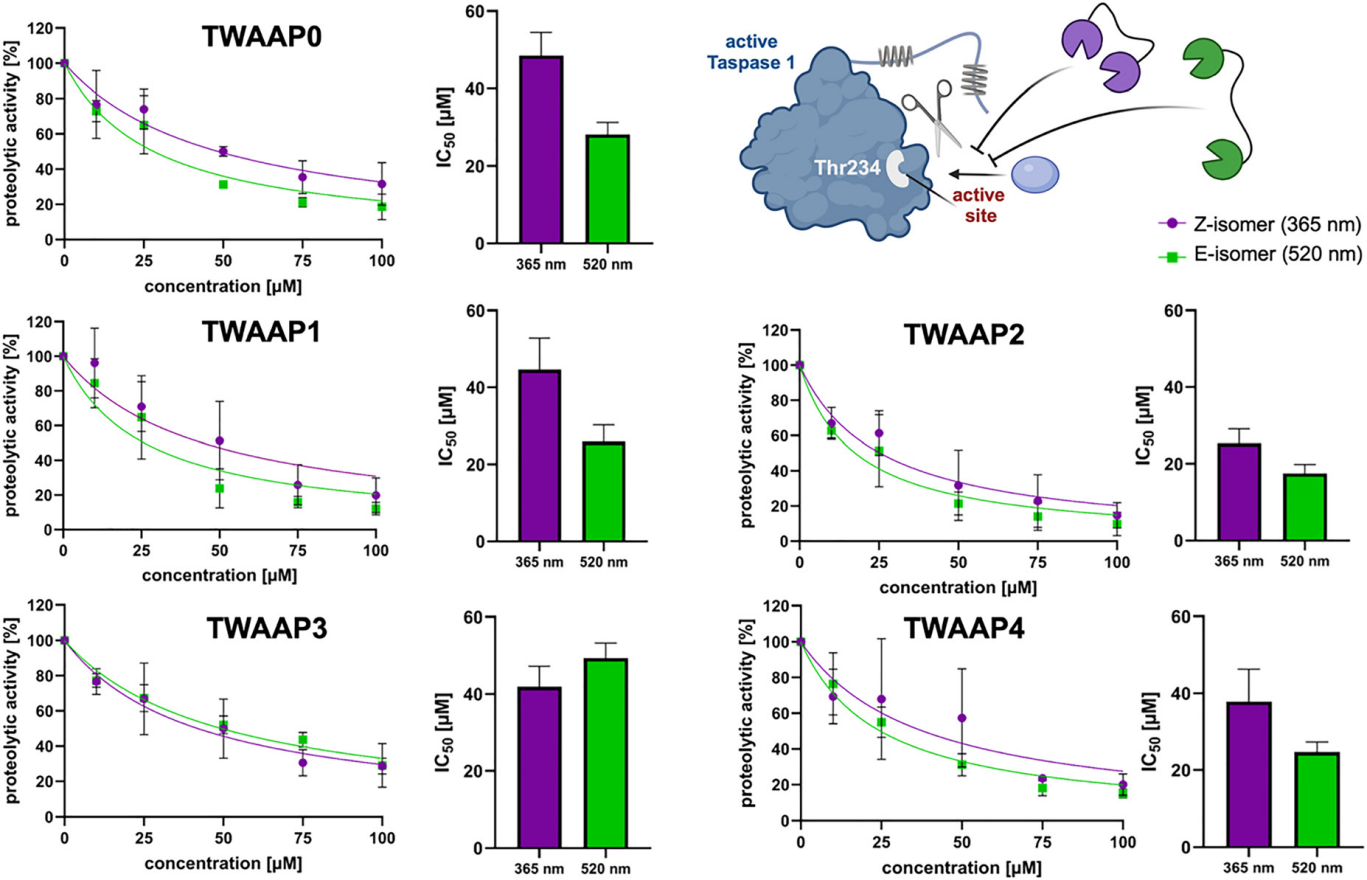
Switchable tweezer constructs interfere with Taspase 1 proteolytic activity. Cleavage assay utilizing the cleavage site CS2 from the natural substrate MLL (GST-MLL_2700–2850_-GFP-His) and active wildtype Taspase 1. Inhibition of proteolysis was evaluated by the relative amount of uncleaved substrate. Briefly, 5 μM recombinant substrate were incubated with 200 nM wildtype Taspase 1-His and increasing concentrations of TWAAP0-4 (10 - 100 μM), pre-irradiated with either UV light (365 nm, violet), or green light (520 nm, green), or solvent only. Proteolytic activity in solvent-treated samples was set to 100%, and data from TWAAP-treated samples were normalized accordingly. IC_50_ values were determined by densitometric quantification of the cleavage bands from the gels shown in Fig. S9, followed by nonlinear curve fitting. These values were also used to generate the summary bar plots shown on the right. Data are shown as the mean ± SD from three independent experiments. Composed with https://BioRender.com.

Given that Taspase 1's active site is positioned directly at the base of the loop harboring the bipartite NLS (Fig. 1A, and Fig. S1), it is reasonable to expect that our photoswitchable tweezer constructs, which recognize this signal, might also influence proteolytic activity - similar to the inhibition observed with our “first-generation” multivalent ligands.^10^ In principle the presence of a sterically demanding artificial ligand may hinder or even completely block substrate binding and subsequent cleavage. To assess this, we employed a well-established *in vitro* cleavage assay using a recombinant, truncated fusion protein harboring the second cleavage site (CS2) of the Mixed Lineage Leukemia 1 (MLL1) protein, a *bona fide* Taspase 1 substrate.^39^ In this assay, the cleaved and uncleaved substrate fractions of the GST-MLL_2700–2850_-GFP-His substrate were separated on a Coomassie-stained gel, and signal intensities of the different cleavage bands were quantified (Fig. 7, Fig. S8, and Fig. S9). The addition of recombinant, proteolytically active Taspase 1 (200 nM) led to efficient substrate cleavage, which was inhibited by all photoswitchable tweezer constructs in a concentration-dependent manner. IC_50_ values for the full series of switchable tweezer constructs ranged from 20 to 50 μM, with measurements taken separately for both *E* and *Z* isomers Fig. (S9). Notably, significant differences in inhibitory activity were observed between these isomers, particularly at 50 μM, for TWAAP0, TWAAP1 and TWAAP4, where the *E* isomer exhibited 1.5–1.7 times greater potency (Fig. 6). In contrast, TWAAP2, TWAAP3 and standard azobenzene dimers TWAB1 and TWAB2 showed no difference in inhibitory activity between their *E* and *Z* isomers (Fig. 7 and Fig. S8 and S9).

### Molecular bridging mode in *E* configuration maximally blocks substrate access

All experimental results were ultimately integrated into *in silico* molecular modeling to gain a deeper understanding of the interaction between our photoswitchable tweezer constructs and the flexible loop of Taspase 1. Molecular dynamics (MD) simulations were first performed using TWAAP1 as a representative switchable tweezer in a discrete water environment.^10^TWAAP1 was positioned either on lysines within the Taspase 1 loop or on lysines near the active site (Tables S5–S12). The flexible loop was analyzed both as an isolated structure and as part of the full-length protein. To explore interactions with the active site, the flexible loop was truncated to mimic autoproteolytic cleavage, allowing substrate access. MD simulations revealed that TWAAP1 can bridge the two α-helices (∼2 nm apart) of the flexible loop in both *E* and *Z* isomers, while only the *Z* isomer spans two lysines within the same basic cluster (Fig. S10 and S11). This behavior remained consistent even when the loop was integrated into the full-length Taspase 1 protein.

**Fig. 8:**
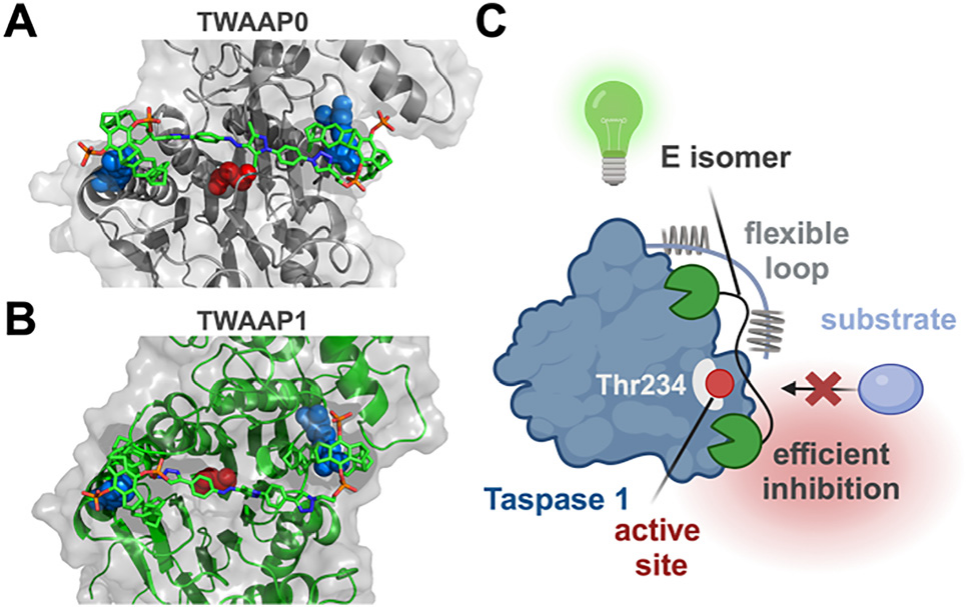
Molecular modelling of photoswitchable tweezer constructs on Taspase 1. TWAAP0 (A) and TWAAP1 (B) were docked onto K57 or K59 and R262, positioned opposite Taspase 1's active site, and subjected to extended MD simulations. Both tweezer molecules remained stably bound to their anchor points, effectively blocking access to the active site, represented by the catalytically active threonine, T234 (magenta), in the Connolly surface representation. Only extended dimeric tweezers in the *E* configuration can span the distance between opposing lysines and sterically obstruct the active site (C). The MD simulation was conducted in discrete water for 200 ns using the MacroModel tool within the Desmond simulation package (Schrödinger Suite). Composed with https://BioRender.com.

The ability of TWAAP1 to bind tightly to the lysine-rich protein loop, without a strong preference for the *E* or *Z* isomer, explains its binding behavior in assays using the Cy5-labeled loop. This situation changes for the much more extended TWAAP4, whose *Z* isomer is only a weak loop binder (Fig. 5). In contrast, the distance between lysines flanking the active site is larger (∼3 nm) and cannot be spanned by the *Z* isomer after switching from the *E* isomer. Additionally, this region is well-folded and compact, unlike the flexible loop. However, four lysines are positioned in pairs around the active site. In this configuration, substrate access is most effectively hindered when the tweezer operates in bridging mode, which requires the *E* configuration. This structural preference aligns with the varying degrees of enzyme inhibition observed for the *E* and *Z* isomers of TWAAP0, TWAAP1, and TWAAP4 in the cleavage assay. In their *E* configuration, all these constructs span the 3 nm gap between the lysines flanking the active site, leading to enhanced inhibition (Fig. 8). Conversely, while the *Z* isomer can still bridge lysine pairs on the same side of the active site, it only provides partial steric hindrance, necessitating higher concentrations for complete inhibition.

With caution, we deduct a preliminary structure activity relationship from the above considerations: Table S4 offers a good synoptic overview of all experimental results. Close inspection reveals that in most experiments, *E*/*Z* values differ only marginally for TWAAP2, TWAAP3 and TWAB1, TWAB2 with relatively short spacers. By contrast, the extended tweezers TWAAP0, TWAAP1 and TWAAP4 show pronounced differences, and in most cases, the *E* isomer is superior with respect to affinity as well as inhibition. Thus, SPR, fluorescence titrations and FP competition experiments show superior binding to the Taspase 1 loop with factors of up to 5 : 1. In line with earlier calculations on multivalent tweezers, we assign increased loop affinity to the ability of the extended tweezers to bridge both α-helices and prevent NLS docking of the receptor. For efficient enzymatic inhibition, however, a direct coverage of the active site would be most favourable, which is again only possible with the longest spacers in their *E* configuration (Fig. 7).

Since four basic residues around the active site offer good anchor points, we explain the most efficient inhibition of proteolysis by TWAAP0, TWAAP1 and TWAAP4 with their *E* isomer spanning the active site and preventing substrate access. Most likely, however, tweezer binding to the loop region also contributes to enzyme inhibition, so that the *E*/*Z* difference remains more modest (∼2 : 1).

## Conclusions

The rigid coupling of two molecular tweezers to either end of AAP photoswitches of varying lengths generates novel divalent protein ligands capable of targeting lysine clusters on protein surfaces. This design enables precise control over the distance between tweezer tips, with a significant shift in spacing between the *E* and *Z* isomers. The photoswitches can be repeatedly toggled between a predominantly *E* or *Z* isomer without compromising configurational integrity or undergoing photodegradation. These dimeric tweezers exhibit high nanomolar affinity for lysine-rich regions of the Taspase 1 protein. As a proof of concept, they were employed to disrupt the interaction between Taspase 1 and its receptor protein, Importin α, effectively inhibiting the enzyme's proteolytic activity. While no significant difference in protein–protein interaction (PPI) inhibition was observed between isomers, larger tweezer constructs in the *E* configuration exhibited notably stronger inhibitory effects. This enhanced activity likely results from the *E* isomer's ability to bridge flanking lysines on opposite sides of the enzyme's active site. Looking ahead, these switchable dimeric tweezers offer a powerful tool for precisely modulating protein function in other lysine-rich targets with two rigid lysine clusters. By leveraging visible light to toggle between isomeric states, researchers could dynamically regulate protein activity, enabling selective functional disruption and real-time studies of protein interactions.

## Author contributions

ALFB, AMAK, KH, MB, FCN, CB: investigation, methodology, formal analysis, validation, visualization; LM: investigation; PB: resources, project administration, funding acquisition; SKK, BJR, TS: conceptualization, methodology, formal analysis, data curation, resources, supervision, visualization, project administration, funding acquisition, writing – original draft, writing – review & editing. All authors contributed to writing the manuscript.

## Conflicts of interest

There are no conflicts to declare.

## Supplementary Material

CB-006-D5CB00069F-s001

CB-006-D5CB00069F-s002

## Data Availability

The data supporting this article have been included as part of the SI. Supplementary information is available. See DOI: https://doi.org/10.1039/d5cb00069f.
