# Supplementary material for: Photoresponsive molecular tweezers modulate Taspase 1 activity
Source: RSC Chem Biol. 2025 Sep 5;6(12):1837–47. doi: 10.1039/d5cb00069f (PMC12419465; doi:10.1039/d5cb00069f)
Supplement: CB-006-D5CB00069F-s002 [file CB-006-D5CB00069F-s002.pdf]

Supplementary Information (SI) for RSC Chemical Biology.
This journal is © The Royal Society of Chemistry 2025

Electronic Supplementary Information

Photoresponsive Molecular Tweezers Modulate Taspase 1 Activity

Antonio L. Figueroa Bietti[a]‡, Alisa-Maite A. Kauth[b]‡, Katrin Hommel[c]‡, Mike Blueggel[c], Laurenz Mohr[c], Felix C. Niemeyer[a], Christine Beuck[d], Peter Bayer[d], Shirley K. Knauer*[c], Bart Jan Ravoo[b], and Thomas Schrader*[a]

*correspondence to:

thomas.schrader@uni-due.de, b.j.ravoo@uni-muenster.de, shirley.knauer@uni-due.de

Experimental Procedures

Syntheses. All commercially available chemicals in this work were purchased from Sigma-Aldrich, Fisher Chemicals, VWR, Fluka, Acros Organics or TCI Chemicals and were used without further purification.

Synthesis of dialkynyl switch elements. Dialkynyl arylazopyrazoles were synthesized as published: A.-M. Kauth, R. Niebuhr, B. J. Ravoo, *J. Org. Chem.* **2024**, *89*, 6371–6376.

Dialkynyl azobenzene derivatives were synthesized as described below:

4,4´-Diethynylazobenzene

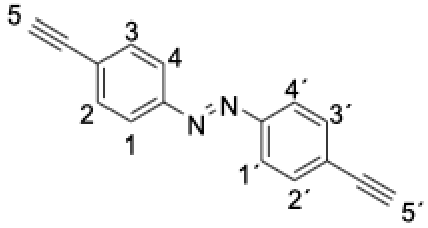

A 250 ml one-necked flask was filled with 2.5 g (21 mmol, 1 eq.) 4-ethynylaniline in 150 ml toluene and stirred. 42 g (483 mmol, 23 eq.) MnO_2 was added, and the reaction mixture was stirred for another 3.5 h at 120 °C. Subsequently, the reaction mixture was filtered over a D4-glas filter and the filtrate was evaporated to dryness. Pure product was obtained as a red shiny solid (0.8 g, 3.4 mmol, 32 %).

^{1}H NMR (400 MHz, Chloroform-d): δ = 7.91 - 7.86 (dt, 4H, H-1, H-1´,H-4, H-4´), 7.66 – 7.61 (dt, J = 5.8, 4.3 Hz, 2H, H-2, H-2´, H-3, H-3´), 3.24 (s, 2H, H-5, H-5´) ppm

^{13}C NMR (151 MHz, Chloroform-d): δ = 152.24, 133.36, 132.99, 125.18, 123.23, 123.16, 123.06, 83.46, 83.27, 80.29, 79.39 ppm

ESI-MS: for $C_{16}H_{10}N_2$ calc. 230.27 g/mol, found 230.27 g/mol

(4,4´-Dihydroxymethyl)azobenzene

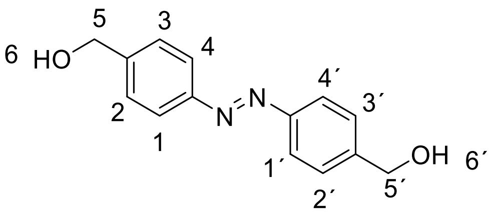

A 250 ml three-necked flask equipped with reflux condenser and dropping funnel was filled under Argon with 4-nitrobenzyl alcohol (3.0 g, 19.5 mmol, 1 eq.) in 30 ml ethanol. Aqueous NaOH (12.5 g, 310 mmol, 16 eq. in 75 mL H_2O) was added and the reaction mixture was stirred for 30 min at 50 °C. Subsequently, an aqueous glucose solution was added dropwise (19.6 g, 110 mmol, 5.55 eq. in 40 mL H_2O) and the solution was stirred for another hour at 50 °C. Argon supply was removed, and the solution was further stirred overnight at room temperature. Subsequently, acetic acid was added until pH 5 was reached, and the reaction mixture was filtered over a D4 glass filter. Finally, the filtrate was lyophylized and the product was obtained as orange needles (260 mg, 1.07 mmol, 11 %).

^{1}H NMR (400 MHz, Chloroform-d): δ = 9.75 (s, 2H, H-6, H-6´), 7.69 (d, J = 6.9 Hz, 2H, H-1, H-1´, H-4, H-4´), 6-70(d, J = 8.9 Hz, 2H, H-2, H-2´, H-3, H-3´), 4,22 (bs, 4H, H-5, H-5´) ppm

^{13}C NMR (151 MHz, Chloroform-d): δ = 190.55, 152.42, 133.99, 132.48, 127.85, 114.60, 114.23, 77.36 ppm

ESI-MS: for $C_{14}H_{14}N_2O_2$ calc. 242.28 g/mol, found 242.28 g/mol

4,4-´Bis(prop-2-yn-1-yloxymethyl)azobenzene

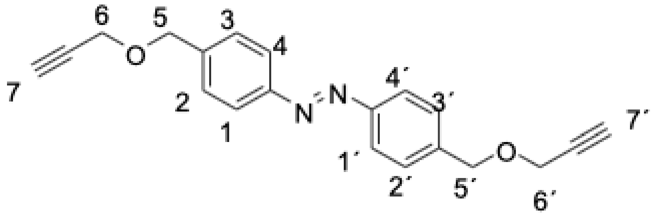

In a 250 ml round bottom flask 1.27 g (5.2 mmol, 1 eq.) of the benzylic alcohol were dissolved in freshly distilled dry THF and cooled to 0 °C. The solution was carefully treated with 0.302 g (12.58 mmol, 2.4 eq.) sodium hydride and the reaction mixture was stirred for 10 min. Subsequently, 1.4 ml (12.58 mmol, 2.4 eq.) propargyl bromide was added and the reaction mixture was stirred overnight and slowly warmed to room temperature. Full conversion was monitored with TLC and the reaction was terminated with careful addition of 20 ml water. The resulting mixture was extracted with dichloromethane (3 x 50 ml) and the combined organic phases were washed with saturated aqueous NaCl (3 x 50 mL). The organic phase was dried over $MgSO_4$ and the solvent was removed in vacuo. The crude product was purified by column chromatography over silica gel (cyclohexane/ethyl acetate 10:1) and obtained as yellow solid (0.527 g, 1.65 mmol, 32%).

^{1}H NMR (400 MHz, Chloroform-d): δ = 7.88 (d, 4H, H-1, H-1´,H-4, H-4´), 7.54 (d, J = 5.8, 4.3 Hz, 2H, H-2, H-2´, H-3, H-3´), 4.63 (s, 4H, H-5, H-5´), 4,60 (m, 4H, H-5, H-5´), 4.28 – 4.21 (m, 4H, H-6, H-6´), 3.51 (t, 2H, H-7, H-7´) ppm

^{13}C NMR (151 MHz, Chloroform-d): δ = 152.37, 140.57, 128.67, 123.09, 79.57, 75.03, 71.14, 57.47 ppm

HR ESI-MS: $[M+H^{+}]^{+}$ for $C_{20}H_{19}N_2O_2$ calc. 319.1441 g/mol, found 319.1442 g/mol

Note: Isolated spacers with terminal phosphate groups could serve in principle as reference compounds; however, the isolated spacers, specifically the photoswitchable units bearing terminal alkynes, are insoluble in water or aqueous buffer. While attaching phosphate groups to these spacers could improve their solubility, it would introduce artificial phosphate moieties not present in the actual tweezer conjugates.

Synthesis of the azidotweezer. The tweezer derivative carrying a terminal azido moiety was synthesized as published: Höing, A.; Kirupakaran, A.; Beuck, C.; Pörschke, M.; Niemeyer, F.; Seiler, T.; Hartmann, L.; Bayer, P.; Schrader, T.; Knauer, S., *Biomacromolecules* **2022**, *23*, 4504-4518.

Connection between tweezers and switch via Click methodology.

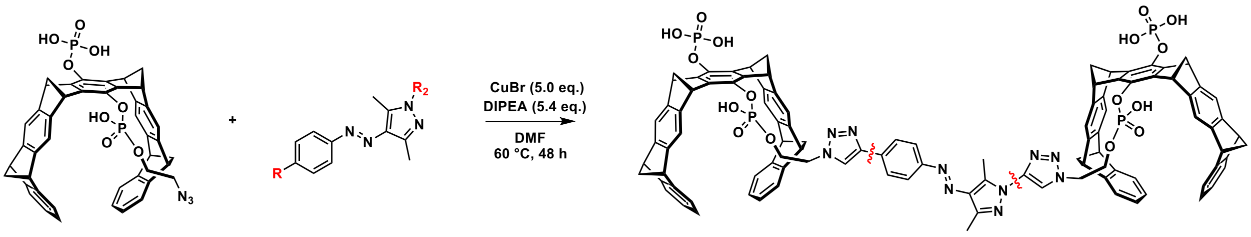

Scheme S1. Optimized CuAAc reaction between two azidotweezer units and one dialkynyl arylazopyrazole switch element leading to the new ditopic photoswitchable molecular tweezers. Critical parameters are pure DMF solvent, 5 equivs. CuBr, 5.4 equivs. DIPEA and iterative coupling of two equivalents of azidotweezer **20** to the dialkynyl switch at 40 °C (for the detailed procedure see main text – Experimental Procedures).

Characterization of new dimeric tweezers: Free phosphoric acids

TWAAP0

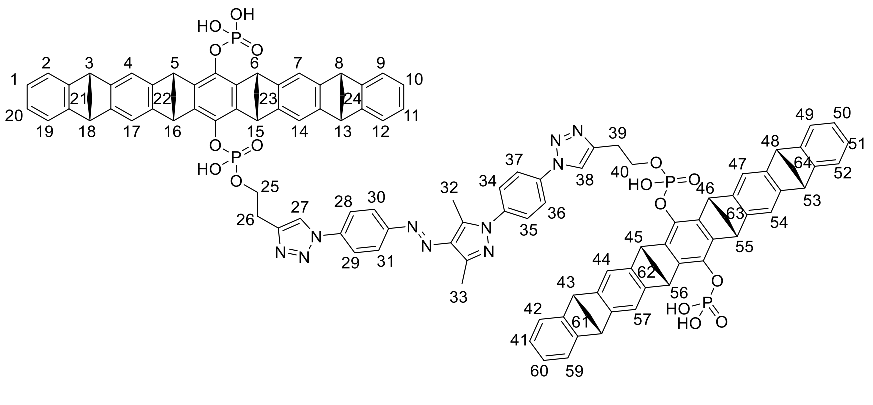

Chemical Formula: $C_{109}H_{86}N_{10}O_{16}P_4$
Molecular Weight: 1915,84

Yield: 40 %

^{1}H NMR (400 MHz, DMSO-d$_6$): δ = δ 8.87 (s, 2H, H-27, H-38), 8.12 (m, 4H, H-28, H-29, H-37, H-36), 8.01 (d, J = 8.7 Hz, 2H, H-30, H-31), 7.87 (d, J = 8.4 Hz, 2H, H-34, H-35), 7.17 – 6.94 (m, 16H, H-2, H-4, H-7, H-9, H-12, H-14, H-17, H-19, H-42, H-44, H-47, H-49, H-52, H-54, H-57, H-59), 6.76 (m, 8H, H-1, H-10, H-11, H-20, H-41, H-50, H-51, H-60), 4.36 – 4.26 (m, 8H, H-5, H-6, H-15, H-16), 4.21 (m, 4H, H-26, H-39), 4.06 (m, 8H, H-3, H-8, H-13, H-18), 3.06 (m, 4H, H-25, H-40), 2.74 (s, 3H, H-32), 2.52 (s, 3H, H-33), 2.34 – 2.11 (m, 16H, H-21, H-22, H-23, H-24, H-61, H-62, H-63, H-64) ppm

^{31}P NMR (243 MHz, DMSO-d$_6$): δ = -3.76, -4.15 ppm

HRMS: calc. $[M\text{-}H^+]^{2+}$ 958.7676 g/mol, found 958.7654 g/mol

Note: The free phosphoric acids of **TWAAP0-4** were not soluble in sufficient amounts to obtain ^{13}C NMR spectra, as opposed to their sodium salts. Here ^{13}C NMR spectra are reported. Similarly, ^{31}P NMR spectra could not be obtained from the phosphoric acids of **TWAB1-2** but were available from their sodium salts (vide infra). Unfortunately, no HRMS spectrum was obtained for **TWAAP2**.

TWAAP1

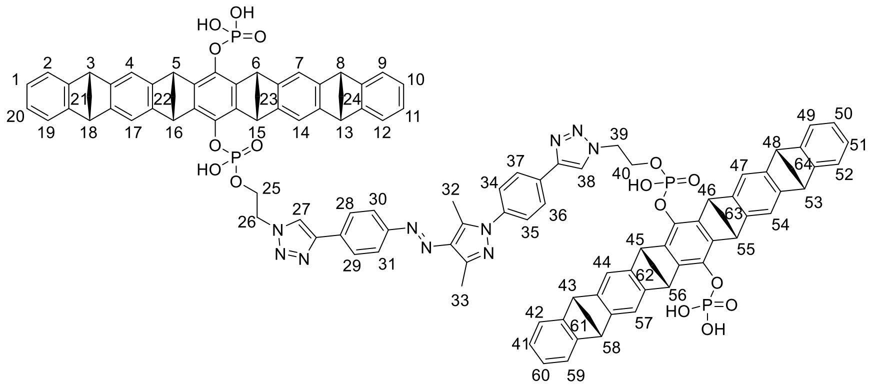

Chemical Formula: $C_{109}H_{86}N_{10}O_{16}P_4$
Molecular Weight: 1915,84

Yield: 37 %

^{1}H NMR (400 MHz, DMSO-d$_6$): δ = 8.76 (s, 2H), 8.10 – 7.69 (m, 8H), 7.06 (m, 16H H-2, H-4, H-7, H-9, H-12, H-14, H-17, H-19, H-42, H-44, H-47, H-49, H-52, H-54, H-57, H-59), 6.76 (m, 8H, H-1, H-10, H-11, H-20, H-41, H-50, H-51, H-60), 4.62 (s, 4H H-26, H-39), 4.37 – 4.17 (m, 12H, H-5, H-6, H-15, H-16, H-25, H-40), 4.12 – 4.01 (m, 8H H-3, H-8, H-13, H-18), 2.68 (d, J = 20.4 Hz, 6H, H-32, H-33), 2.32 – 2.15 (m, 16H, H-21, H-22, H-23, H-24, H-61, H-62, H-63, H-64) ppm

^{31}P NMR (243 MHz, DMSO-d$_6$): δ = -5.43, -6.0 ppm

HRMS: $[M-H^+]^{2+}$ calc. 958.7676 g/mol, found 958.7660 g/mol

TWAAP2

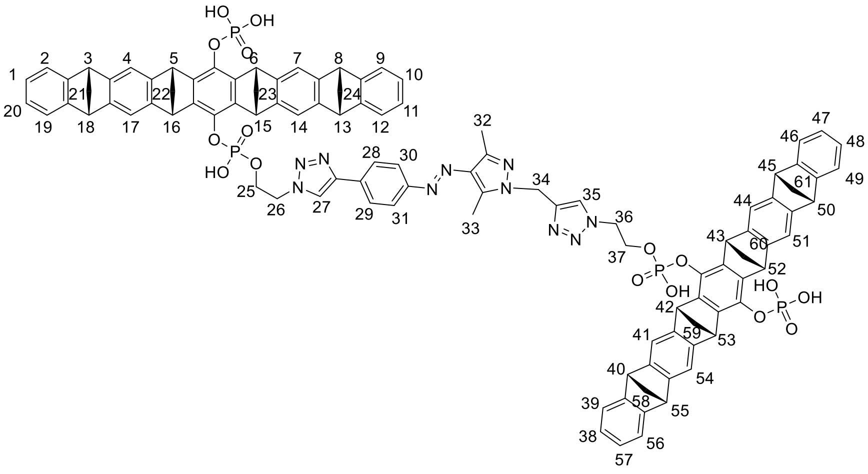

Chemical Formula: $C_{104}H_{84}N_{10}O_{16}P_4$
Molecular Weight: 1853,77

Yield: 38 %

^{1}H NMR (400 MHz, DMSO-d$_6$): δ = 8.79 (s, 2H, H-27, H-35), 8.20 – 7.78 (m, 4H, H28, H-29, H-30, H-31), 7.17 – 6.97 (m, 16H H-2, H-4, H-7, H-9, H-12, H-14, H-17, H-19, H-39, H-41, H-44, H-46, H-49, H-51, H-54, H-56), 6.82 – 6.69 (m, 8H, H-1, H-10, H-11, H-20, H-38, H-47, H-48, H-57), 5.45 (s, 2H, H-34), 4.63 (s, 2H, H-26), 4.50 (s, 2H, H-36), 4.37 – 4.27 (m, 8H, H-5, H-6, H-15, H-16), 4.25 – 4.20 (m, 4H, H-25, H-37), 4.10 – 4.03 (m, 8H; H-3, H-8, H-13, H-18, H-40, H-45, H-50, H-55), 2.75 – 2.66 (s, 3H, H-32), 2.38 (s, 3H, H-33), 2.33 – 2.13 (m, 16H, H-21, H-22, H-23, H-24, H-58, H-59, H-60, H-61) ppm

^{31}P NMR (243 MHz, DMSO-d$_6$): δ = -5.49, -5.98 ppm

TWAAP3

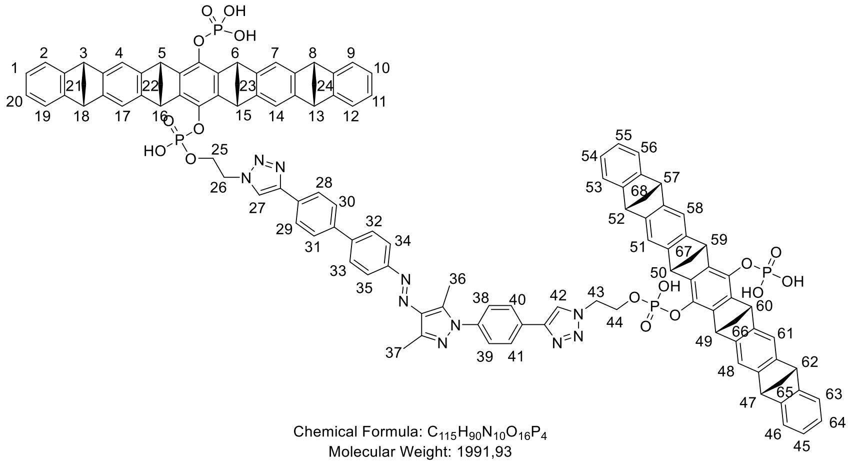

Yield: 34 %

^{1}H NMR (400 MHz, Dimethylsulfoxid-d$_6$): δ = 8.79 (m, 2H, H-27, H-42), 8.12 – 7.79 (m, 12H, H-28, H-29, H-30, H-31, H-32, H-33, H-34, H-35, H-38, H-39, H-40, H-41), 7.12 – 6.96 (m, 16H H-2, H-4, H-7, H-9, H-12, H-14, H-17, H-19, H-46, H-48, H-51, H-53, H-56, H-58, H-61, H-63), 6.76 (m, 8H, H-1, H-10, H-11, H-20, H-45, H-54, H-55, H-64), 4.58 (s, 4H, H-26, H-43), 4.33 – 4.19 (m, 12H, H-5, H-6, H-15, H-16, H-25, H-44, H-49, H-50, H-59, H-60), 4.05 (m, 8H, H-3, H-8, H-13, H-18, H-47, H-52, H-57, H-62), 2.89 (s, 3H, H-36), 2.73 (s, 3H, H-37), 2.29 – 2.12 (m, 16H, H-21, H-22, H-23, H-24, H-65, H-66, H-67, H-68) ppm

^{31}P NMR (243 MHz, Dimethylsulfoxid-d$_6$): δ = -5.44, -5.93 ppm

HRMS: $[M-H^+]^{2+}$ calc. 996.7832 g/mol, found 996.7830 g/mol

TWAAP4

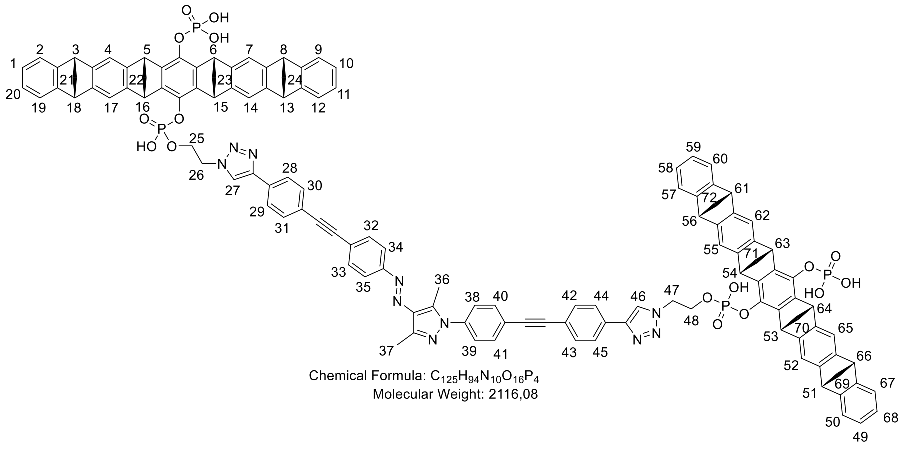

Chemical Formula: $C_{125}H_{94}N_{10}O_{16}P_4$
Molecular Weight: 2116,08

Yield: 29 %

^{1}H NMR (400 MHz, DMSO-d$_6$): δ = 8.80 (s, 2H, H-27, H-46), 7.97 (m, 4H, H-28, H-29, H-44, H-45), 7.77 – 7.59 (m, 12H, H-30, H-31, H-32, H-33, H-34, H-35, H-38, H-39, H-40, H-41, H-42, H-43), 7.10 – 6.98 (m, 16H, H-2, H-4, H-7, H-9, H-12, H-14, H-17, H-19, H-50, H-52, H-55, H-57, H-60, H-62, H-65, H-67), 6.76 (m, 8H, H-1, H-10, H-11, H-20, H-49, H-58, H-59, H-68), 4.51 (s, 4H, H-26, H-47), 4.28 (m, 8H, H-5, H-6, H-15, H-16, H-53, H-54, H-63, H-64), 4.14 (s, 4H, H-25, H-48), 4.05 (m, 8H, H-3, H-8, H-13, H-18, H-51, H-56, H-61, H-66), 2.89 (s, 3H, H-36), 2.72 (s, 3H, H-37), 2.29 – 2.12 (m, 16H, H-21, H-22, H-23, H-24, H-69, H-70, H-71, H-72) ppm

^{31}P NMR (243 MHz, DMSO-d$_6$): δ = 5.39, - 5.54 ppm

HRMS: $[M\text{-}H^+]^{2+}$ calc. 1058.7989 g/mol, found 1058.7990 g/mol

TWAB1

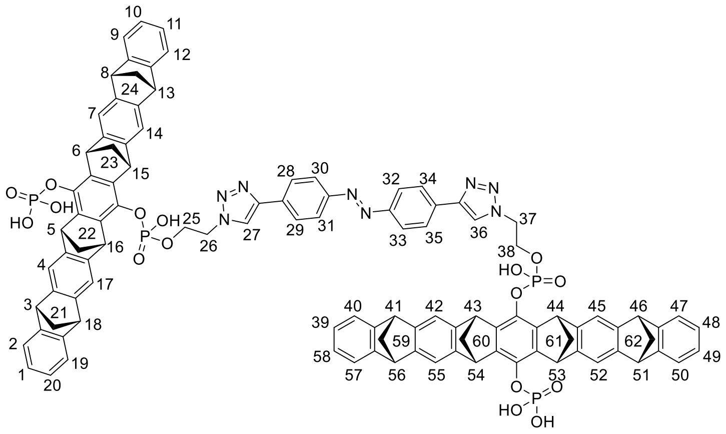

Chemical Formula: $C_{104}H_{80}N_8O_{16}P_4$
Molecular Weight: 1821,72

Yield: 42 %

^{1}H NMR (400 MHz, DMSO-d$_6$): δ = 8.87 (s, 2H, H-27. H-36), 8.14 (m, 2H, H-28, H-29), 8.03 (m, 2H, H-34, H-35), 7.95 (s, 2H, H-30, H-33), 7.93 – 7.90 (m, 1H, H-31), 7.71 (d, *J* = 8.3 Hz, 1H, H-32), 7.18 – 7.00 (m, 16H, H-2, H-4, H-7, H-9, H-12, H-14, H-17, H-19, H-40, H-42, H45, H-47, H-50, H-52, H-55, H-57), 6.77 (m, 8H, H-1, H-10, H-11, H-20, H-39, H-48, H-49, H-58), 4.63 (s, 2H, H-26,), 4.48 (s, 2H, H-37), 4.31 (m, 8H, H-5, H-6, H-15, H-16, H-43, H-44, H-53, H-54), 4.23 (s, 2H, H-25, H-38), 4.10 – 4.01 (m, 8H, H-3, H-8, H-13, H-18, H-41, H-46, H-51, H-56), 2.32 – 2.13 (m, 16H, H-21, H-22, H-23, H-24, H-59, H-60, H-61, H-62) ppm

^{13}C NMR (151 MHz, DMSO-d$_6$): δ = 162.30, 150.27, 146.91, 146.85, 146.76, 146.65, 141.29, 140.95, 133.01, 126.11, 124.43, 123.42, 122.99, 121.45, 116.77, 67.79, 50.28, 48.00, 47.88, 40.06, 35.78, 30.77 ppm

HRMS: $[M-H^+]^{2+}$ calc. 911.7410 g/mol, found 911.7384 g/mol

TWAB2

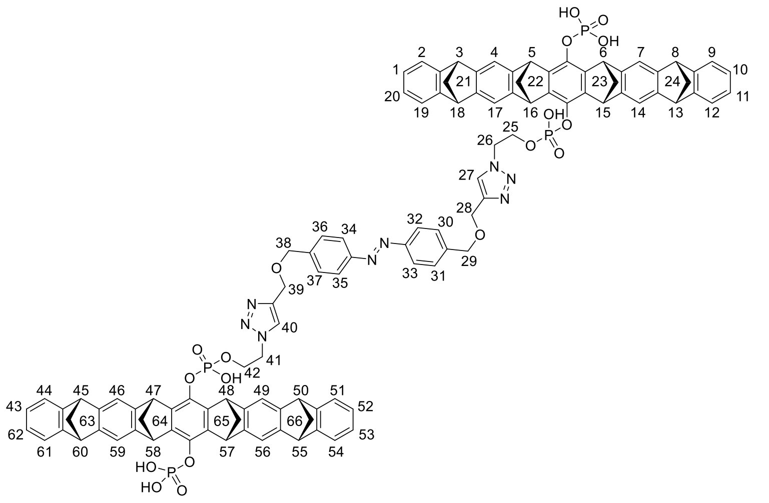

Chemical Formula: $C_{108}H_{88}N_8O_{18}P_4$
Molecular Weight: 1909,83

Yield: 37 %

^{1}H NMR (400 MHz, DMSO-d_6): δ = 8.44 – 8.07 (m, 2H, H-27, H-40), 8.03 – 7.42 (m, 8H, H-30, H-31, H-32, H-33, H-34, H-35, H-36, H-37), 7.22 – 6.93 (m, 16H, H-2, H-4, H-7, H-9, H-12, H-14, H-17, H-19, H-44, H-46, H-49, H-51, H-54, H-56, H-59, H-61), 6.77 (m, 8H, H-1, H-10, H-11, H-20, H-43, H-52, H-53, H-62), 4.71 – 4.49 (m, 8H, H-28, H-29, H-38, H-39), 4.32 (m, 4H, H-5, H-6, H-57, H-58), 4.27 – 4.21 (m, 4H, H-15, H-16, H-47, H-48), 4.12 – 4.01 (m, 8H, H-3, H-8, H-13, H-18, H-45, H-50, H-55, H-60), 2.34 – 2.12 (m, 16H, H-21, H-22, H-23, H-24, H-63, H-64, H-65, H-66) ppm

^{13}C NMR (151 MHz, DMSO-d_6): δ = 151.26, 150.29, 147.46, 146.97, 146.94, 146.91, 146.87, 146.78, 146.70, 146.66, 143.89, 141.90, 141.75, 141.62, 141.30, 140.98, 140.18, 136.62, 136.30, 135.99, 128.58, 128.37, 127.13, 124.59, 124.53, 124.46, 122.58, 122.55, 122.50, 122.44, 121.51, 121.47, 121.37, 119.91, 116.83, 116.78, 116.58, 116.19, 116.13, 70.71, 70.66, 69.09, 68.99, 68.21, 67.80, 65.01, 63.16, 62.45, 57.68, 53.55, 50.30, 50.25, 49.97, 48.01, 47.92, 47.83, 47.72, 46.76, 46.70, 40.06, 18.08, 13.97, 1.15 ppm

HRMS: [M-H^{+}] calc. 1910.5272 g/mol, found 1910.5244 g/mol

Neutralization of the free phosphoric acids to their corresponding sodium salts:

Careful neutralization of the diphosphoric acid tweezer derivatives with 4.0 equivalents of 1 M aqueous NaOH in a 1:1 water/THF mixture produced the corresponding tetrasodium salts in quantitative yield. After stirring at 1h at ambient temperature, THF was removed by distillation and the remaining aqueous solution water was lyophilized to dryness. All tetrasodium salts are very well water soluble in methanol and water and were used for all binding experiments and bioassays.

Characterization of new dimeric tweezers: Sodium salts

TWAAP0

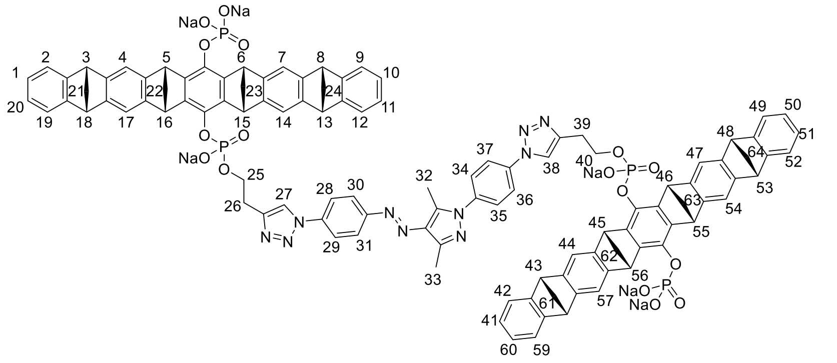

Chemical Formula: $C_{109}H_{80}N_{10}Na_6O_{16}P_4$
Molecular Weight: 2047,73

Yield: 100 %

^{1}H NMR (600 MHz, methanol-d_4): δ = 8.65 (s, 2H, H-27, H-38), 8.19 – 7.87 (m, 5H, H-28, H-29, H-36, H-37, H-30), 7.85 – 7.69 (m, 3H, H-31, H-34, H-35), 7.24 – 6.71 (m, 24H,H-1, H-2, H-4, H-7, H-9, H-10, H-11, H-12, H-14, H-17, H-19, H-20, H-42, H-44, H-47, H-49, H-52, H-54, H-57, H-59), 4.51 – 4.41 (m, 8H, H-5, H-6, H-15, H-16, H-45, H-46, H-55, H-56), 4.03 – 3.90 (m, 8H, H-26, H-39), 3.69 – 3.60 (m, 8H, H-3, H-8, H-13, H-18, H-43, H-48, H-53, H-58), 2.85 – 2.70 (m, 3H, H-32), 2.66 – 2.58 (m, 3H, H-33), 2.40 – 2.16 (m, 16H, H-21, H-22, H-23, H-24, H-61, H-62, H-63, H-64).ppm

^{13}C NMR (151 MHz, methanol-d_4): δ = 127.49, 125.97, 125.89, 124.62, 122.87, 122.80, 122.56, 122.43, 122.37, 122.14, 118.26, 117.81, 117.49, 117.47, 117.15, 68.76, 68.61, 68.47, 68.36, 68.20, 68.05, 52.68, 52.66, 50.15, 50.10, 50.02, 49.93, 49.87, 49.67, 49.53, 49.37, 49.23, 49.08, 48.93, 26.21, 26.08, 25.94, 25.56, 14.80, 11.78 ppm

TWAAP1

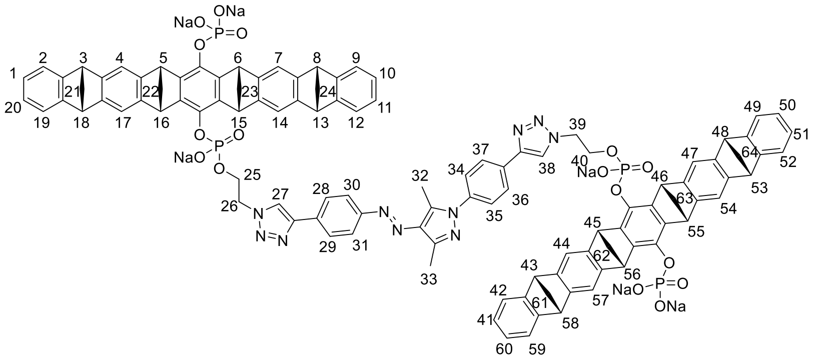

Chemical Formula: $C_{109}H_{80}N_{10}Na_6O_{16}P_4$
Molecular Weight: 2047,73

Yield: 100 %

^{1}H NMR (600 MHz, methanol-d_4): δ = 8.70 – 8.62 (m, 2H), 8.27 – 8.22 (m, 1H), 8.16 (td, *J* = 7.0, 3.1 Hz, 2H), 8.02 – 7.96 (m, 2H), 7.75 – 7.68 (m, 2H), 7.37 – 6.65 (m, 24H), 4.86 (d, *J* = 4.7 Hz, 7H), 4.72 (s, 4H), 4.43 (d, *J* = 10.5 Hz, 3H), 4.04 – 3.93 (m, 8H), 2.78 (s, 2H), 2.64 (s, 3H), 2.52 – 2.17 (m, 17H) ppm

^{13}C NMR (151 MHz, methanol-d_4): δ = 170.34, 161.51, 154.58, 152.27, 152.06, 150.09, 149.95, 149.93, 148.44, 148.04, 147.95, 147.53, 144.97, 143.57, 141.62, 139.72, 138.22, 137.30, 134.11, 133.91, 133.51, 132.47, 127.85, 127.60, 126.83, 126.17, 126.14, 125.85, 123.94, 123.86, 123.64, 122.98, 122.09, 117.93, 117.90, 117.32, 69.41, 68.95, 65.40, 52.42, 52.35, 51.39, 49.86, 49.81, 49.58, 39.70, 39.55, 39.41, 39.28, 30.79, 30.34, 28.13, 14.44, 14.23, 11.41 ppm

TWAAP2

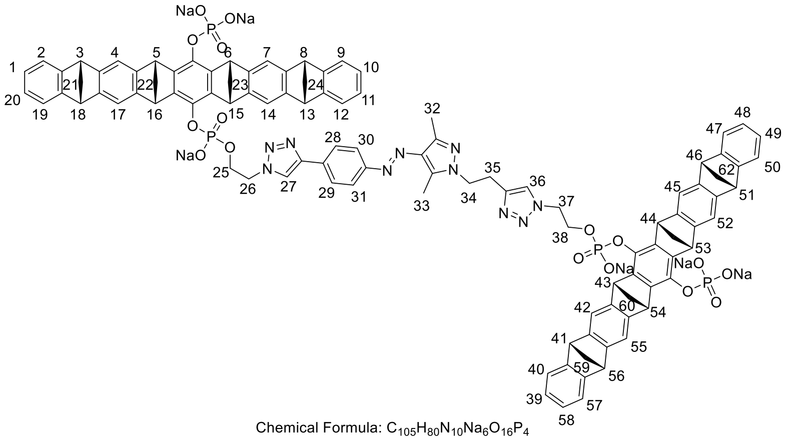

Yield: 100 %

^{1}H NMR (600 MHz, methanol-d$_4$): δ = 8.58 (d, J = 35.4 Hz, 1H), 8.17 (d, J = 4.1 Hz, 1H), 8.12 – 8.05 (m, 1H), 7.93 – 7.82 (m, 1H), 7.32 (d, J = 3.4 Hz, 1H), 7.29 – 6.46 (m, 24H), 5.57 – 5.32 (m, 2H), 4.87 – 4.78 (m, 8H), 4.66 (d, J = 66.7 Hz, 3H), 4.48 – 4.34 (m, 3H), 4.02 – 3.90 (m, 7H), 2.89 (s, 2H), 2.48 (s, 3H), 2.46 – 2.16 (m, 16H) ppm

^{13}C NMR (151 MHz, methanol-d$_4$): δ = 170.50, 161.64, 154.74, 152.37, 152.35, 152.33, 152.18, 152.11, 150.52, 150.18, 150.08, 150.03, 148.61, 148.57, 148.46, 148.40, 148.23, 148.12, 148.07, 144.05, 143.71, 142.97, 141.76, 141.65, 141.20, 138.35, 136.85, 136.80, 133.26, 131.01, 127.64, 126.28, 126.19, 126.03, 125.98, 125.95, 125.92, 123.90, 123.61, 122.24, 122.20, 122.13, 122.10, 122.05, 118.09, 118.00, 117.44, 117.37, 117.20, 116.69, 69.55, 69.36, 69.14, 69.10, 65.56, 65.39, 52.55, 52.47, 52.34, 51.57, 51.24, 50.00, 49.93, 49.82, 49.72, 49.68, 45.37, 39.98, 39.84, 39.70, 39.56, 39.42, 36.69, 33.21, 30.93, 30.74, 30.64, 30.59, 30.48, 30.45, 28.27, 27.06, 23.88, 14.59, 14.47, 10.45 ppm

TWAAP3

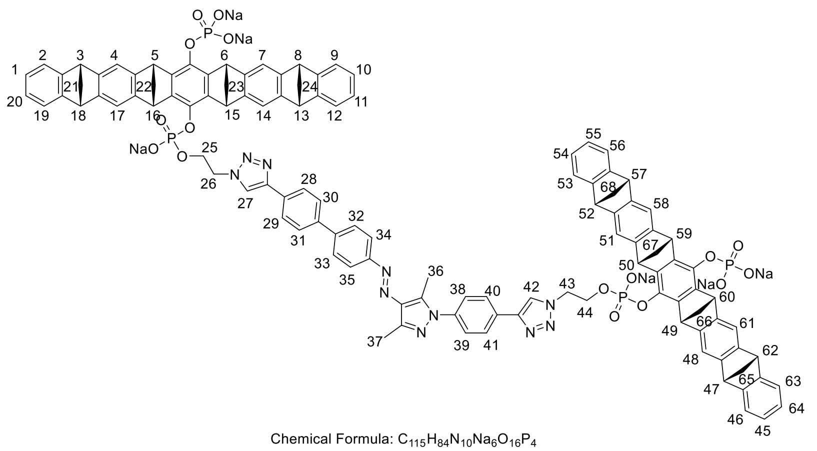

Chemical Formula: $C_{115}H_{84}N_{10}Na_6O_{16}P_4$
Molecular Weight: 2123,82

Yield:100 %

^{1}H NMR (600 MHz, methanol-d$_4$): δ = 8.71 – 8.53 (m, 2H), 8.25 (d, J = 8.1 Hz, 1H), 8.17 – 8.10 (m, 1H), 8.00 – 7.94 (m, 2H), 7.93 – 7.80 (m, 4H), 7.77 – 7.68 (m, 2H), 7.37 – 6.65 (m, 24H), 4.86 – 4.80 (m, 8H), 4.75 (s, 3H), 4.49 – 4.35 (m, 4H), 4.04 – 3.92 (m, 8H), 2.81 – 2.74 (m, 3H), 2.63 (d, J = 2.8 Hz, 3H), 2.52 – 2.17 (m, 17H) ppm

^{13}C NMR (151 MHz, methanol-d$_4$): δ = 161.65, 154.35, 152.42, 152.36, 152.19, 150.65, 150.31, 150.17, 148.57, 148.37, 148.15, 148.03, 147.65, 145.08, 143.76, 141.67, 139.86, 138.29, 137.44, 132.63, 128.73, 128.69, 128.00, 127.66, 126.97, 126.33, 125.97, 124.10, 123.73, 122.23, 122.04, 118.12, 117.38, 117.12, 69.58, 69.09, 65.54, 52.57, 52.50, 50.00, 49.97, 49.82, 49.72, 49.70, 30.93, 30.48, 28.27, 23.88, 14.34, 11.53 ppm

TWAAP4

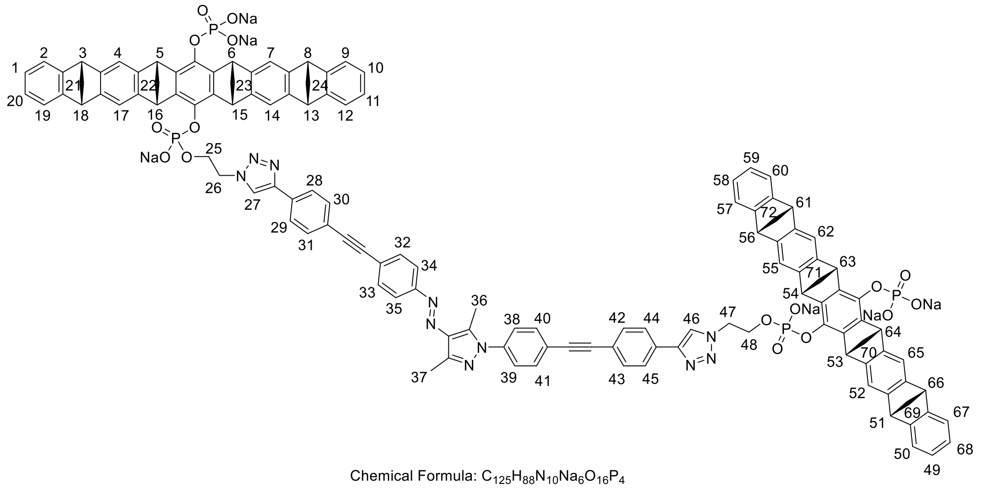

Chemical Formula: $C_{125}H_{88}N_{10}Na_6O_{16}P_4$
Molecular Weight: 2247,97

Yield: 100 %

^{1}H NMR (600 MHz, methanol-d$_4$): δ = 8.73 – 8.42 (m, 2H, H-27, H-47), 8.12 – 7.19 (m, 16H, H-28, H-29, H-30, H-31, H-32, H-33, H-34, H-35, H-38, H-39, H-40, H-41, H-42, H-43, H-44, H-45), 7.17 – 6.63 (m, 24H, H-1, H-2, H-4, H-7, H-9, H-10, H-11, H-12, H-14, H-17, H-19, H-20, H-49, H-50, H-52, H-55, H-57, H-58, H-59, H-60, H-62, H-65, H-67, H-68), 4.75 – 4.57 (m, 4H, H-26, H-47), 4.50 – 4.36 (m, 4H, H-25, H-48), 4.04 – 3.94 (m, 8H, H-3, H-8, H-13, H-18, H-51, H-56, H-61, H-66), 2.59 (s, 3H, H-36), 2.49 (s, 3H, H-37), 2.27 – 2.00 (m, 16H, H-21, H-22, H-23, H-24, H-69, H-70, H-71, H-72). ppm

^{13}C NMR (151 MHz, Methanol-d$_4$): δ = 176.83, 170.48, 161.64, 152.76, 150.81, 148.67, 143.64, 142.84, 138.81, 137.63, 133.87, 130.98, 125.98, 124.01, 122.21, 118.24, 117.24, 73.99, 69.14, 66.48, 64.55, 63.46, 61.79, 52.56, 52.50, 49.30, 49.27, 43.06, 37.27, 33.22, 30.93, 30.91, 30.87, 30.77, 30.62, 30.60, 30.46, 30.38, 27.15, 23.88, 14.59 ppm

TWAB1

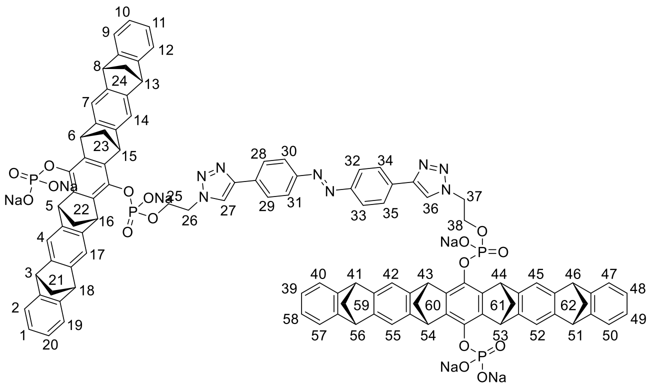

Chemical Formula: $C_{104}H_{74}N_8Na_6O_{16}P_4$
Molecular Weight: 1953,61

Yield: 100 %

^{1}H NMR (600 MHz, methanol-d_4): δ = 8.72 – 8.53 (m, 2H, H-27, H-36), 8.27 – 8.04 (m, 4H, H-28, H-29, H-34, H-35), 7.93 – 7.63 (m, 2H, H-30, H-32), 7.38 – 7.26 (m, 2H, H-31, H-33), 7.20 – 6.63 (m, 24H, H-1, H-2, H-4, H-7, H-9, H-10, H-11, H-12, H-14, H-17, H-19, H-20, H-39, H-40, H-42, H-45, H-, H-47, H-48, H-49, H-50, H-52, H-54, H-57, H-58), 4.63 (s, 4H, H-26, H-37), 4.41 (m, 3H, H-25, H-38), 4.04 – 3.94 (m, 8H, H-5, H-6, H-15, H-16, H-43, H-44, H-53, H-54), 3.70 – 3.56 (m, 8H, H-3, H-8, H-13, H-18, H-41, H-46, H-51, H-56), 2.45 – 2.10 (m, 16H, H-21, H-22, H-23, H-24, H-59, H-60, H-61, H-62) ppm

^{13}C NMR (151 MHz, methanol-d_4): δ = 134.20, 127.84, 126.33, 126.01, 125.98, 125.96, 125.89, 124.83, 124.32, 124.17, 122.25, 122.20, 122.15, 122.08, 122.05, 118.53, 118.11, 117.38, 117.17, 117.13, 116.88, 116.66, 69.70, 52.57, 52.54, 52.50, 50.00, 49.93, 49.87, 49.72, 49.63, 49.38, 49.24, 49.09, 49.06, 48.97, 48.93, 48.78, 48.61, 21.79 ppm

^{31}P NMR (243 MHz, methanol-d_4): δ = 2.64, 1.85, -4.45, -4.9 ppm

TWAB2

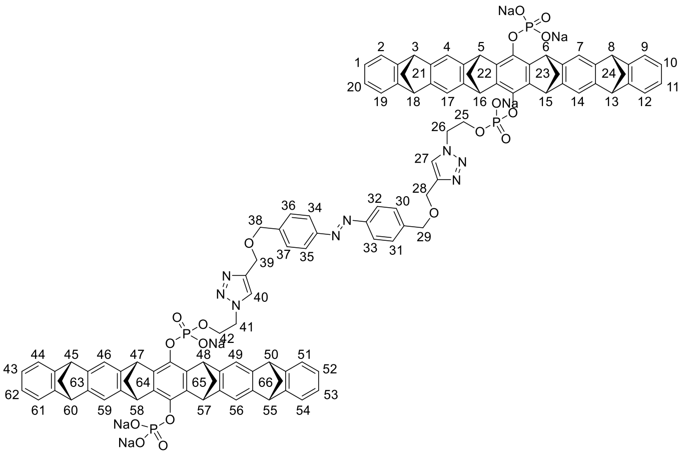

Chemical Formula: $C_{108}H_{82}N_8Na_6O_{18}P_4$
Molecular Weight: 2041,72

Yield: 100 %

^{1}H NMR (600 MHz, methanol-d_4): δ = 8.27 – 7.97 (m, 2H, H-27, H-40), 7.94 – 7.24 (m, 8H, H-30, H-31, H-32, H-33, H-34, H-35, H-36, H-37), 7.23 – 6.61 (m, 24H, H-1, H-2, H-4, H-7, H-9, H-1, H-11, H-12, H-14, H-17, H-19, H-20, H-43, H-44, H-46, H-49, H-51, H-52, H-53, H-54, H-56, H-59, H-61, H-62), 4.85 – 4.76 (m, 8H, H-5, H-6, H-15, H-16, H-47, H-48, H-57, H-58), 4.73 (m, 4H, H-29, H-38), 4.59 (s, 2H, H-28), 4.42 (s, 2H, H-39), 4.02 – 3.91 (m, 8H, H-3, H-8, H-13, H-18, H-45, H-50, H-55, H-60), 2.50 – 2.16 (m, 16H, H-21, H-22, H-23, H-24, H-63, H-64, H-65, H-66) ppm

^{13}C NMR (151 MHz, methanol-d_4): δ = 162.5, 152.3, 150.1, 146.9, 141.5, 137.1, 135.8, 124.3, 121.1, 116.5, 67.8, 67.1, 64.0, 57.9, 50.4, 47.7, 29.4 ppm

^{31}P NMR (243 MHz, methanol-d_4): δ = 1.67, 1.28, -4.73, -5.1 ppm

Supplementary Figures

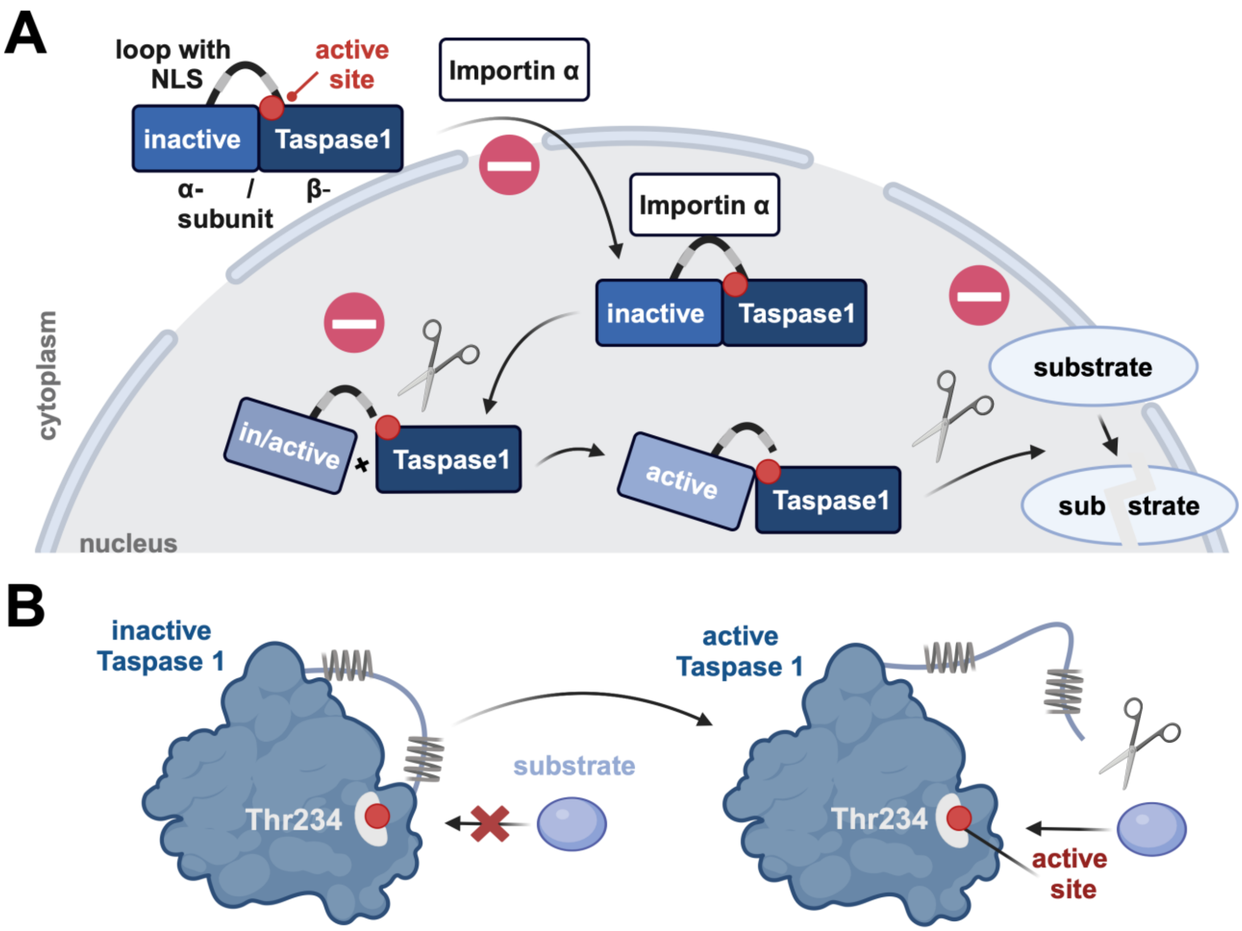

Figure S1. Activation of the protease Taspase 1. The transport of Taspase 1 (blue) into the cell nucleus by Importin α (dark grey, **A**) is followed by its autoproteolytic activation opening the active site (red) for substrate binding and cleavage (**B**). Red signs indicate possible interference points for switchable molecular tweezers. The enzyme is initially shown as the inactive α/β-monomer (medium/dark blue), with the flexible loop containing the NLS (grey) located on the α-subunit (light blue). After nuclear translocation, proteolysis generates the active α/β-heterodimer to enable processing of intracellular substrates (light blue). For simplicity, the active Taspase 1 heterodimer is visualized as a single molecule in **B**. Composed with BioRender.com.

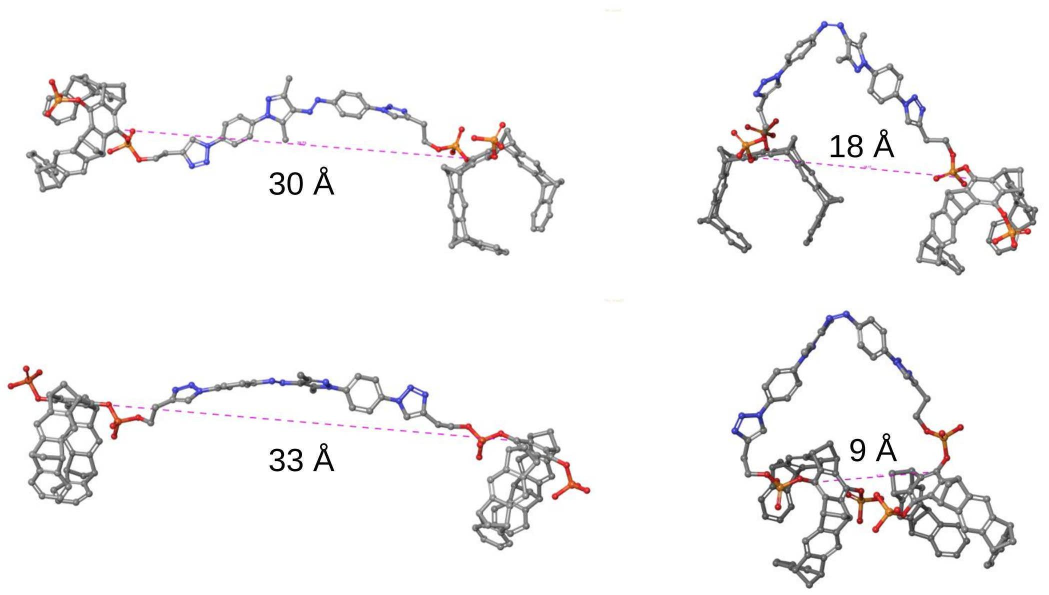

Figure S2a. MD simulation of the dimeric photoswitchable tweezer TWAAP0. Top: Energy-minimized E- and Z-isomer structures, each representing the global minimum of the simulation. Bottom: Extended and compressed E/Z conformations, showing the maximum and minimum distances between the internal tweezer phosphates.

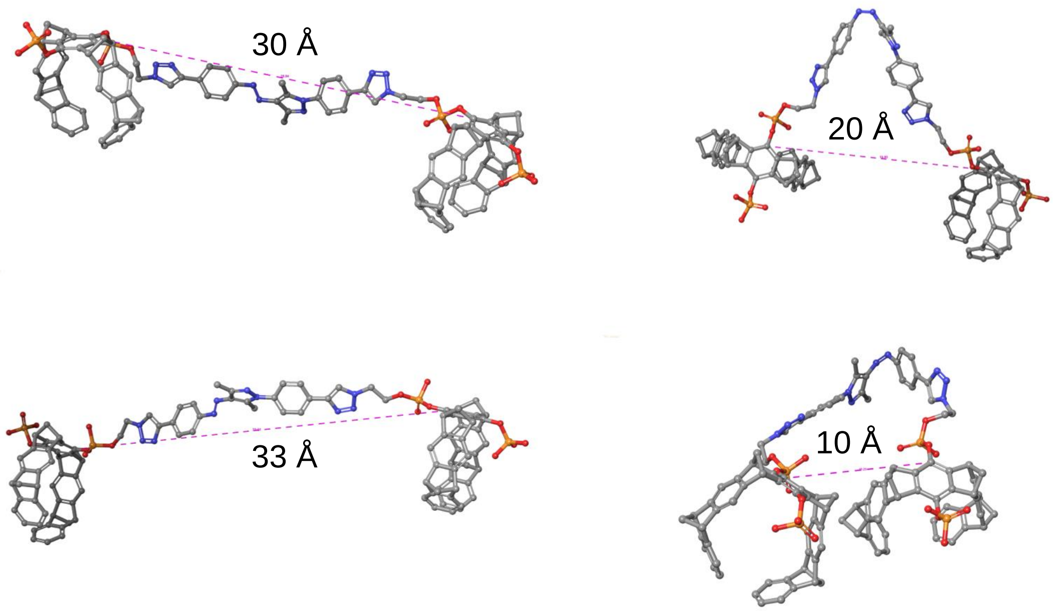

Figure S2b. MD simulation of the dimeric photoswitchable tweezer TWAAP1. Top: Energy-minimized E- and Z-isomer structures, each representing the global minimum of the simulation. Bottom: Extended and compressed E/Z conformations, showing the maximum and minimum distances between the internal tweezer phosphates.

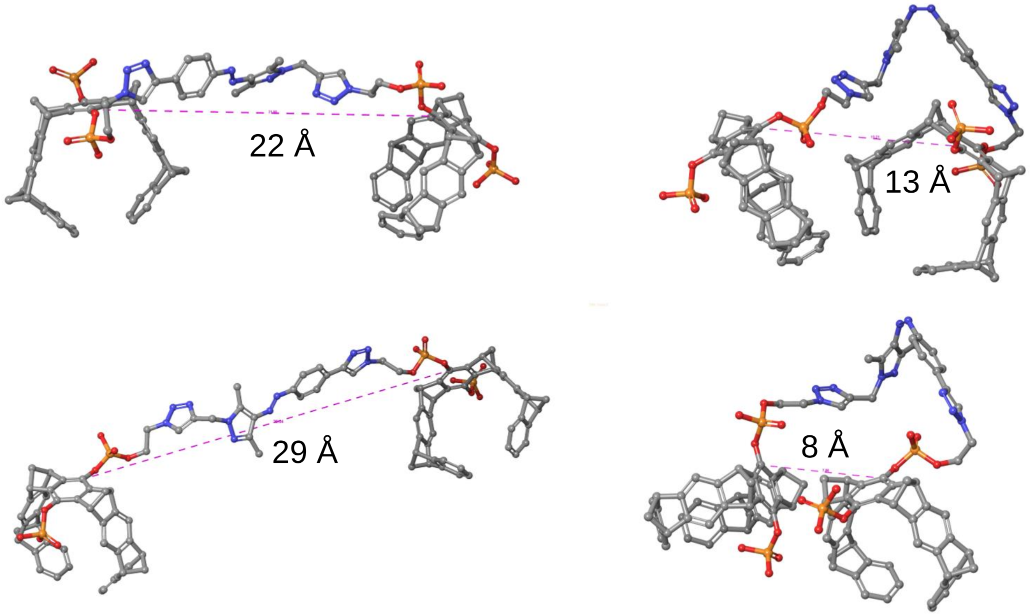

Figure S2c. MD simulation of the dimeric photoswitchable tweezer TWAAP2. Top: Energy-minimized E- and Z-isomer structures, each representing the global minimum of the simulation. Bottom: Extended and compressed E/Z conformations, showing the maximum and minimum distances between the internal tweezer phosphates.

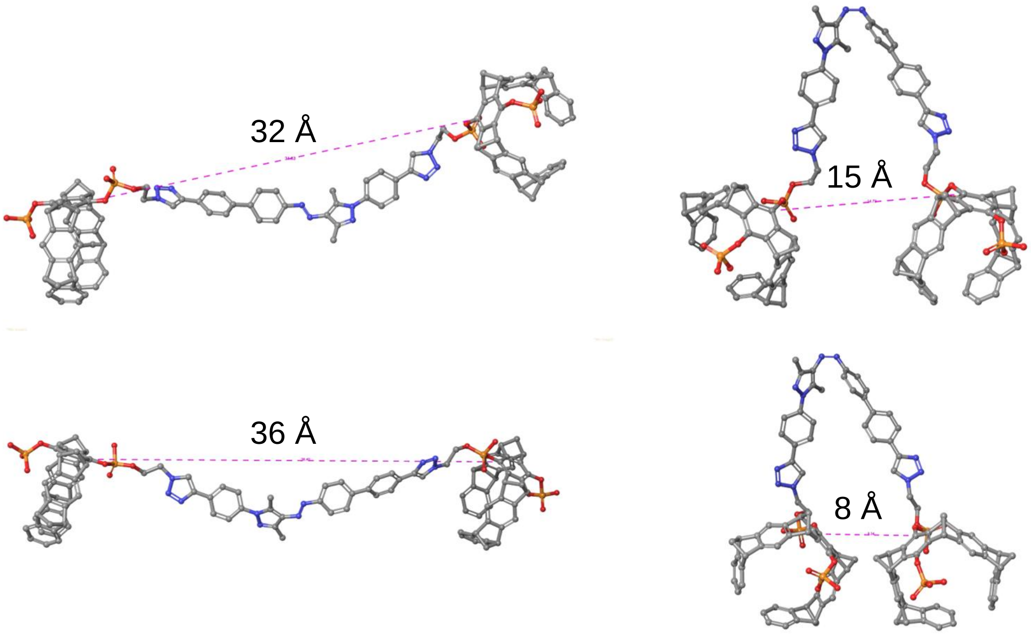

Figure S2d. MD simulation of the dimeric photoswitchable tweezer TWAAP3. Top: Energy-minimized E- and Z-isomer structures, each representing the global minimum of the simulation. Bottom: Extended and compressed E/Z conformations, showing the maximum and minimum distances between the internal tweezer phosphates.

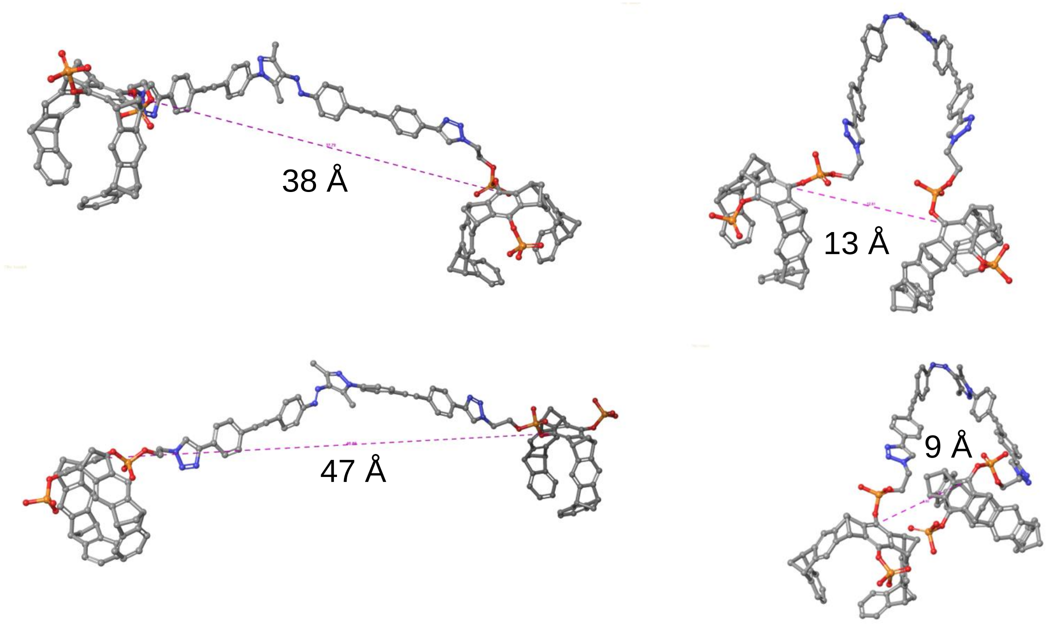

Figure S2e. MD simulation of the dimeric photoswitchable tweezer TWAAP4. Top: Energy-minimized E- and Z-isomer structures, each representing the global minimum of the simulation. Bottom: Extended and compressed E/Z conformations, showing the maximum and minimum distances between the internal tweezer phosphates.

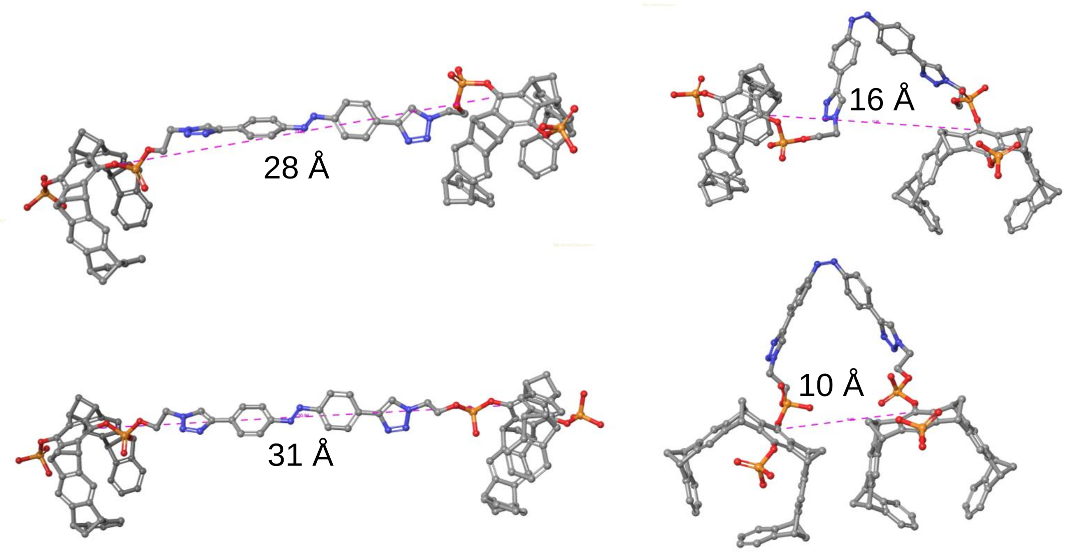

Figure S2f. MD-Simulation of dimeric photoswitchable tweezer TWAB1. Top: minimized E- and Z-structure, each representing the global minimum of the simulation. Bottom: extended and compressed E/Z structure producing maximum and minimum distance between the internal tweezer phosphates.

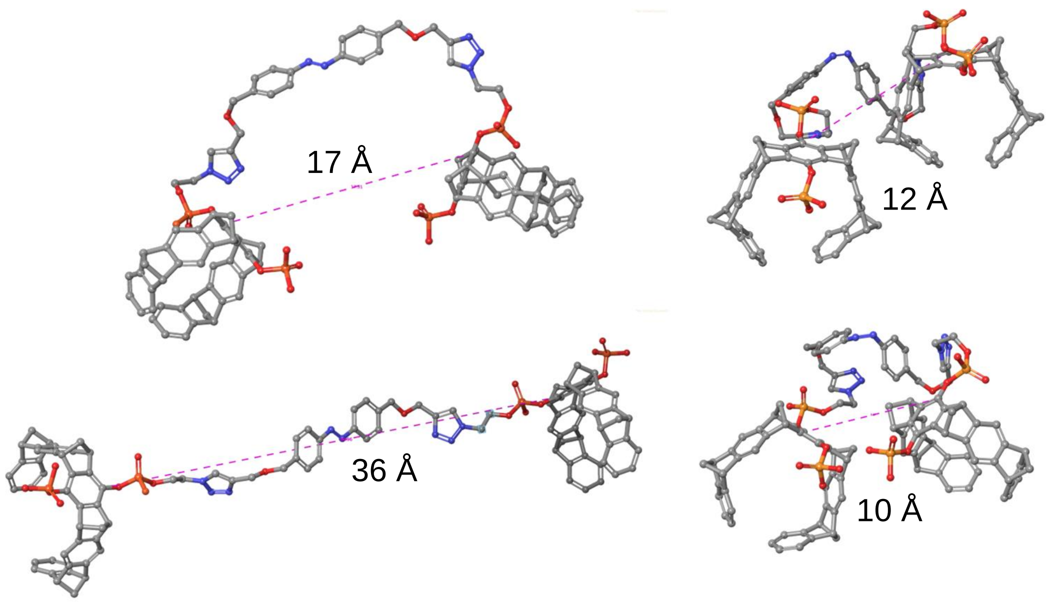

Figure S2g. MD simulation of the dimeric photoswitchable tweezer TWAB2. Top: Energy-minimized E- and Z-isomer structures, each representing the global minimum of the simulation. Bottom: Extended and compressed E/Z conformations, showing the maximum and minimum distances between the internal tweezer phosphates. The geometries presented for the new tweezers in Fig. 2a-g are the final conformations found after extended MD simulations. These and very similar geometries have been identified as the predominant species which were preserved over a long time in the simulation.

Note: In our earlier work, especially in various QM/MM calculations of tweezer conjugates, the distance between the phosphate-carrying C-atoms of the spacer have been used: This also applies here to be consistent with these papers for comparison.

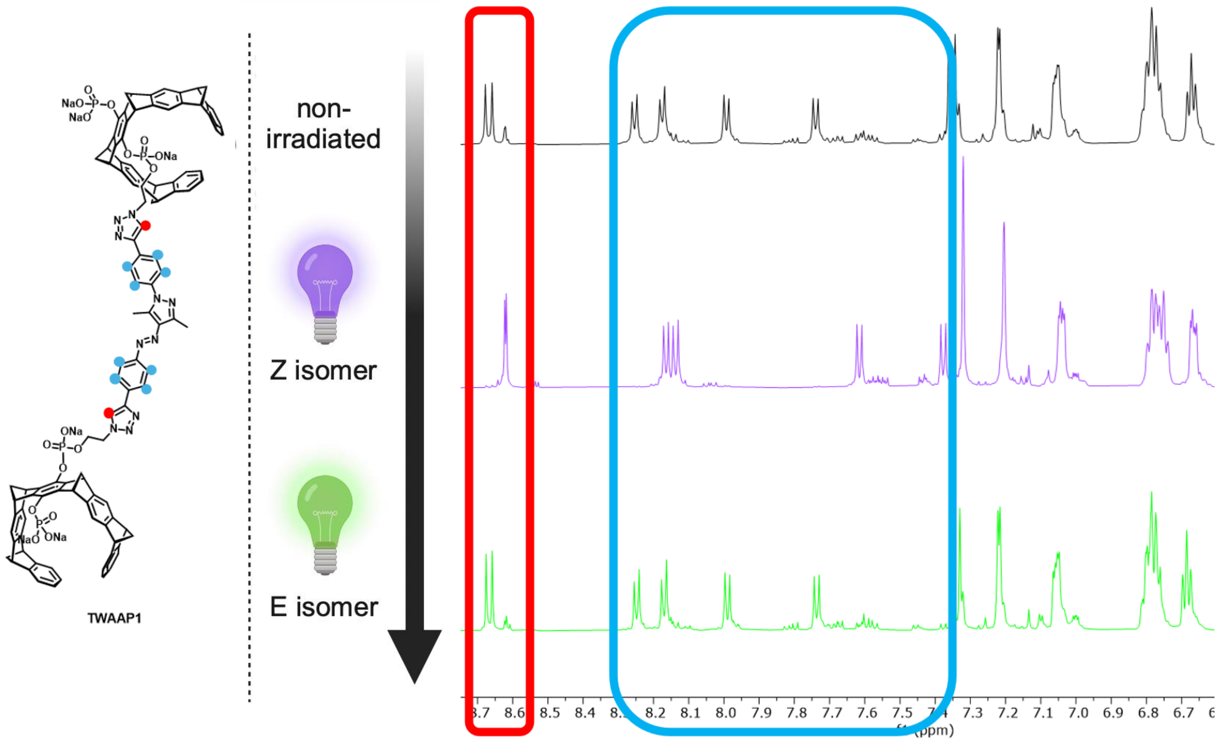

Figure S3. Typical NMR signature of E/Z isomers in photoswitchable tweezers. ^{1}H NMR experiment depicting characteristic changes in signal splitting of the arylazopyrazole switch (blue) and the triazole (red) moiety of **TWAAP1**. Synthetic product mixture (black), majority Z isomer obtained by irradiation with 365 nm for 4 min (violet) and majority E isomer obtained from irradiation with 520 nm for 6 min (green). Close inspection of all three spectra of Tweezer **TWAAP1** in the above Figure reveals additional small signals: The E-isomer carries a small signal (dd) at 7.8 ppm, which however does not appear in the Z-isomer. Conversely, the Z-isomer shows a small signal (dd) at 8.05 ppm, which does not appear in the E-isomer. This is experimental evidence for a compound which is also switched between the E and Z configuration. Most likely this is not the unconnected switch precursor, because such a nonpolar impurity would be separated in the work-up process. We tentatively assign this signal to a minor conformer of the product **TWAAP1** in which one of the triazole rings has been rotated by 180°. Such a compound belongs to the product **TWAAP1**, but displays a slightly shifted set of aromatic signals and will also be switched on irradiation. Integration of the main E- and Z- signals (green and purple spectrum) confirms that the efficiency of switching by light is higher than 90 %.

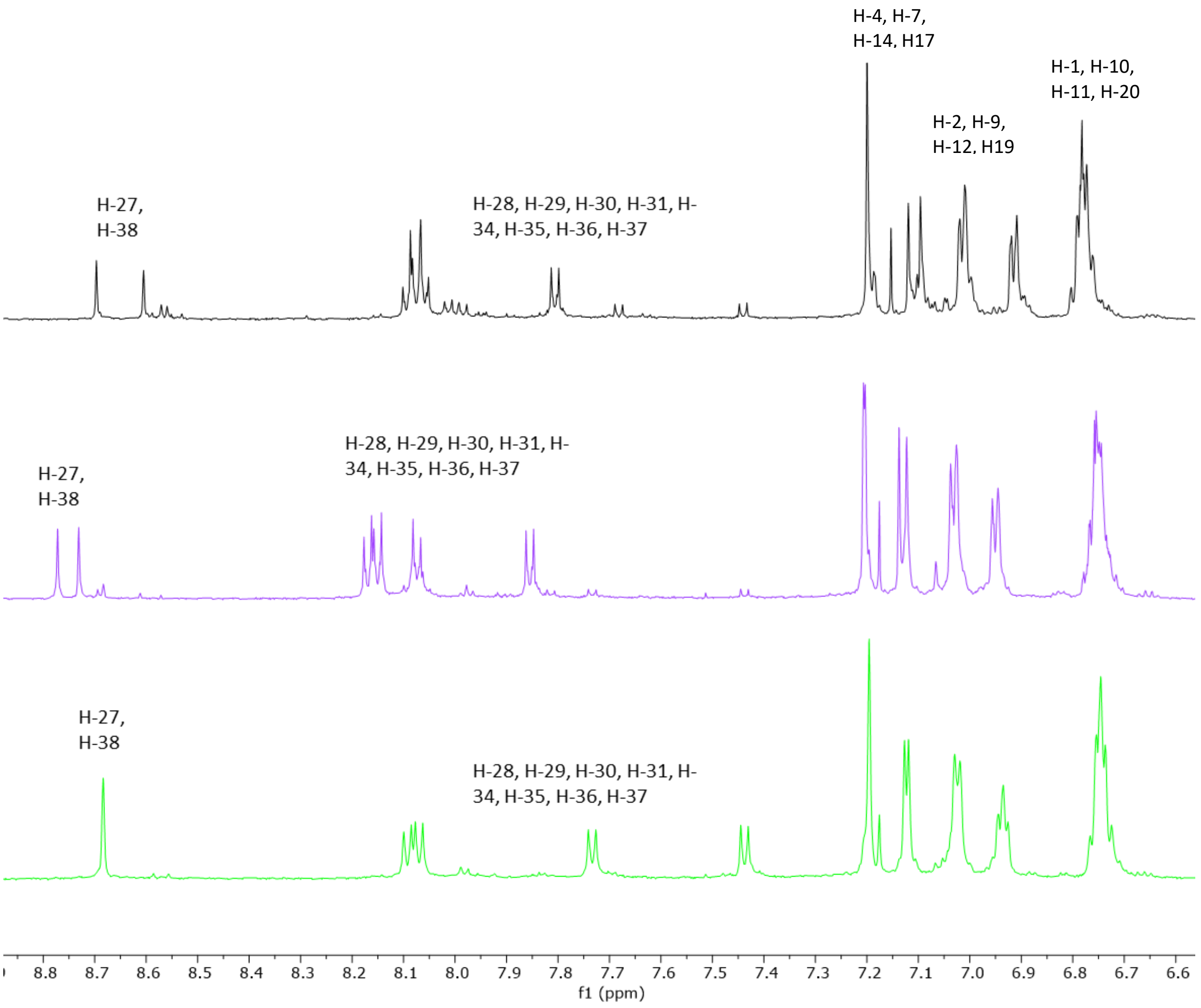

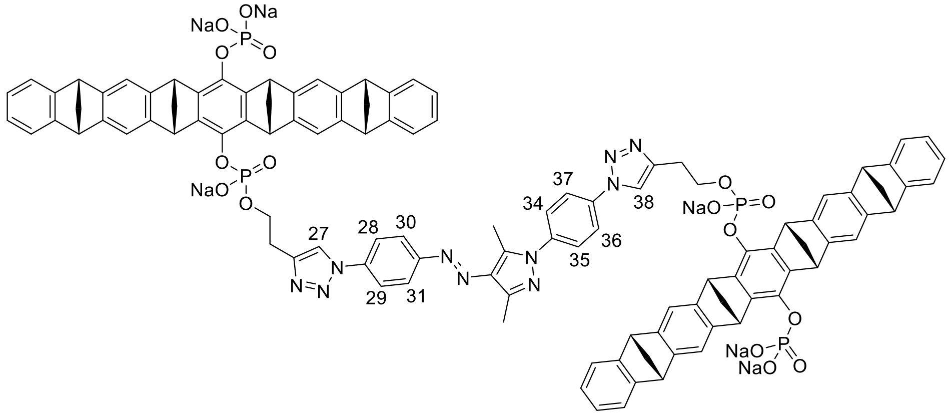

Figure S3a. Full spectra of isolated E/Z isomers of photoswitchable tweezer TWAAP0. ^{1}H NMR experiment depicting all changes in signal splitting. Major changes occur in the arylazopyrazole switch and the triazole moiety of **TWAAP0**. Synthetic product mixture (black), majority Z isomer obtained by irradiation with 365 nm for 4 min (violet) and majority E isomer obtained from irradiation with 520 nm for 6 min (green).

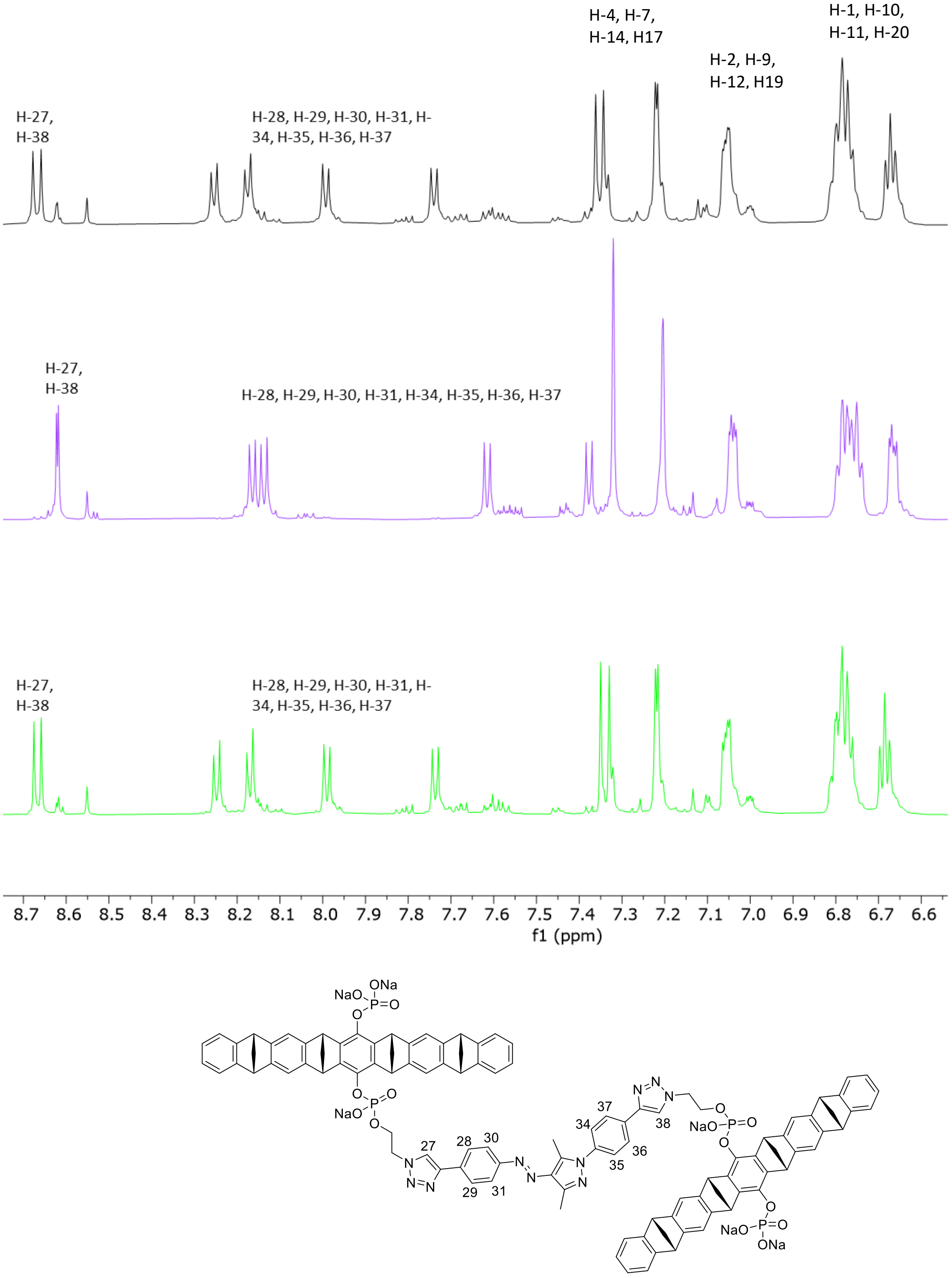

Figure S3b. Full spectra of isolated E/Z isomers of photoswitchable tweezer TWAAP1. ^{1}H NMR experiment depicting all changes in signal splitting. Major changes occur in the arylazopyrazole switch and the triazole moiety of **TWAAP1**. Synthetic product mixture (black), majority Z isomer obtained by irradiation with 365 nm for 4 min (violet) and majority E isomer obtained from irradiation with 520 nm for 6 min (green).

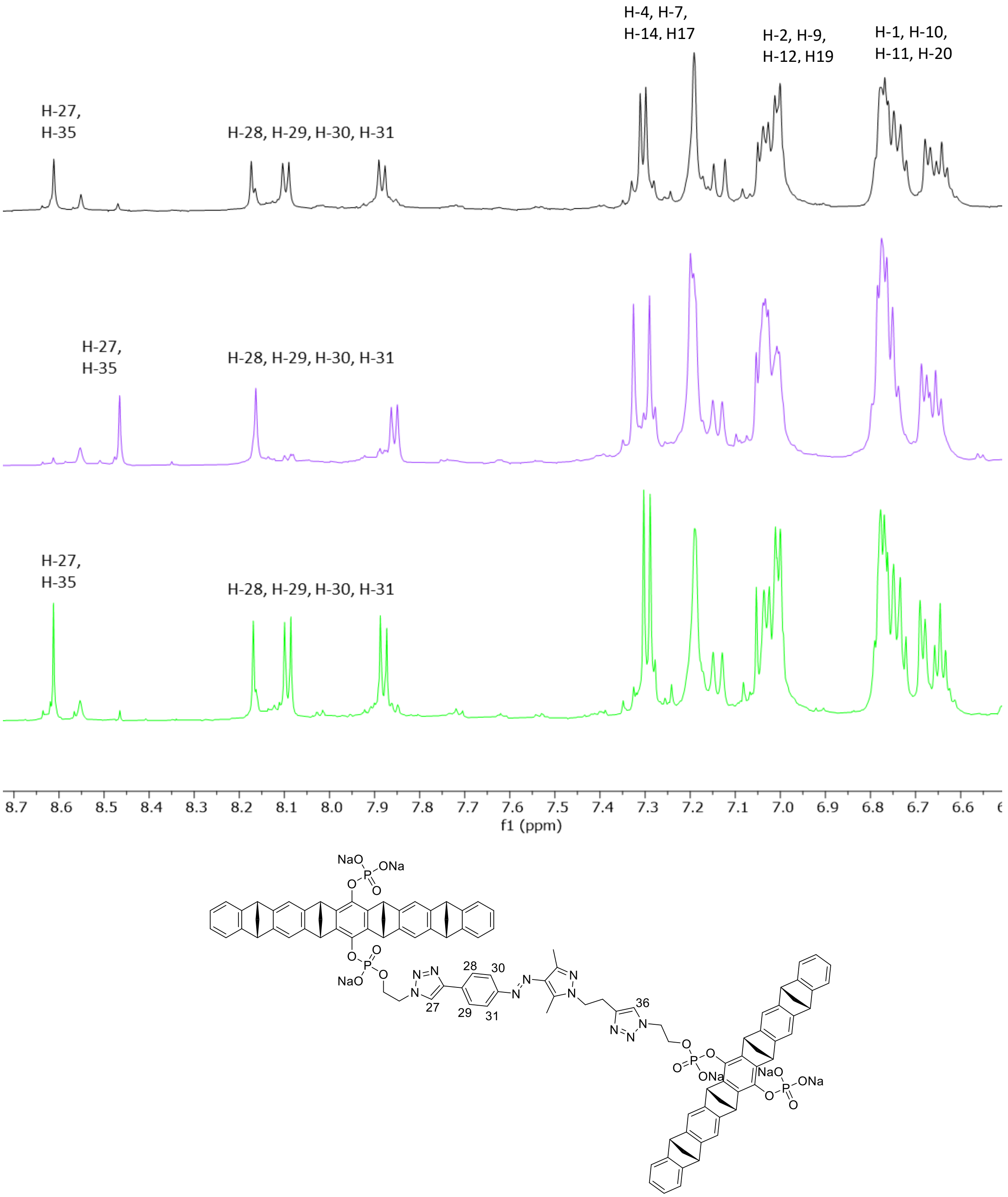

Figure S3c. Full spectra of isolated E/Z isomers of photoswitchable tweezer TWAAP2. ^{1}H NMR experiment depicting all changes in signal splitting. Major changes occur in the arylazopyrazole switch and the triazole moiety of **TWAAP2**. Synthetic product mixture (black), majority Z isomer obtained by irradiation with 365 nm for 4 min (violet) and majority E isomer obtained from irradiation with 520 nm for 6 min (green).

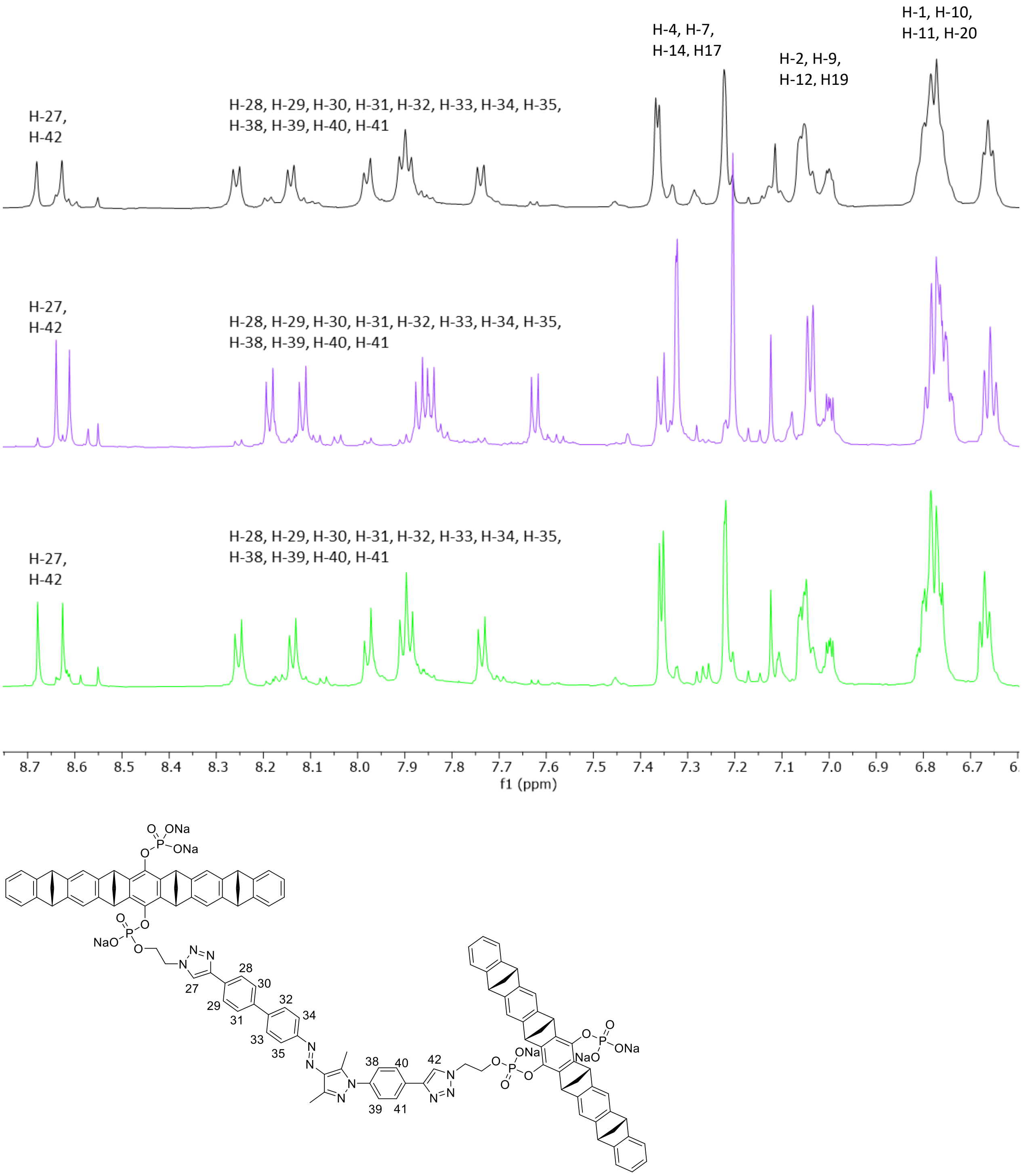

Figure S3d. Full spectra of isolated E/Z isomers of photoswitchable tweezer TWAAP3. ^{1}H NMR experiment depicting all changes in signal splitting. Major changes occur in the arylazopyrazole switch and the triazole moiety of **TWAAP3**. Synthetic product mixture (black), majority Z isomer obtained by irradiation with 365 nm for 4 min (violet) and majority E isomer obtained from irradiation with 520 nm for 6 min (green).

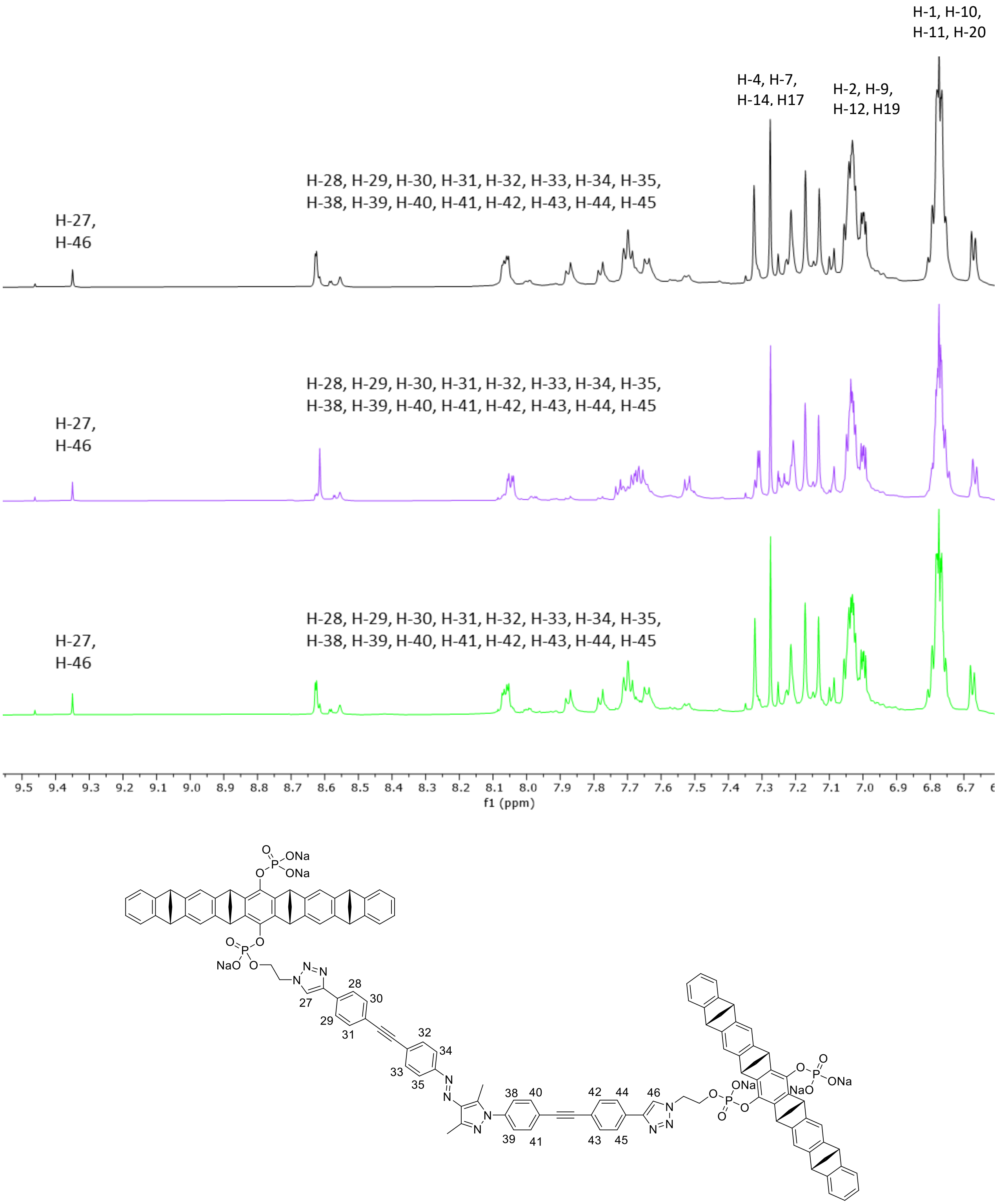

Figure S3e. Full spectra of isolated E/Z isomers of photoswitchable tweezer TWAAP4. ^{1}H NMR experiment depicting all changes in signal splitting. Major changes occur in the arylazopyrazole switch and the triazole moiety of **TWAAP4**. Synthetic product mixture (black), majority Z isomer obtained by irradiation with 365 nm for 4 min (violet) and majority E isomer obtained from irradiation with 520 nm for 6 min (green).

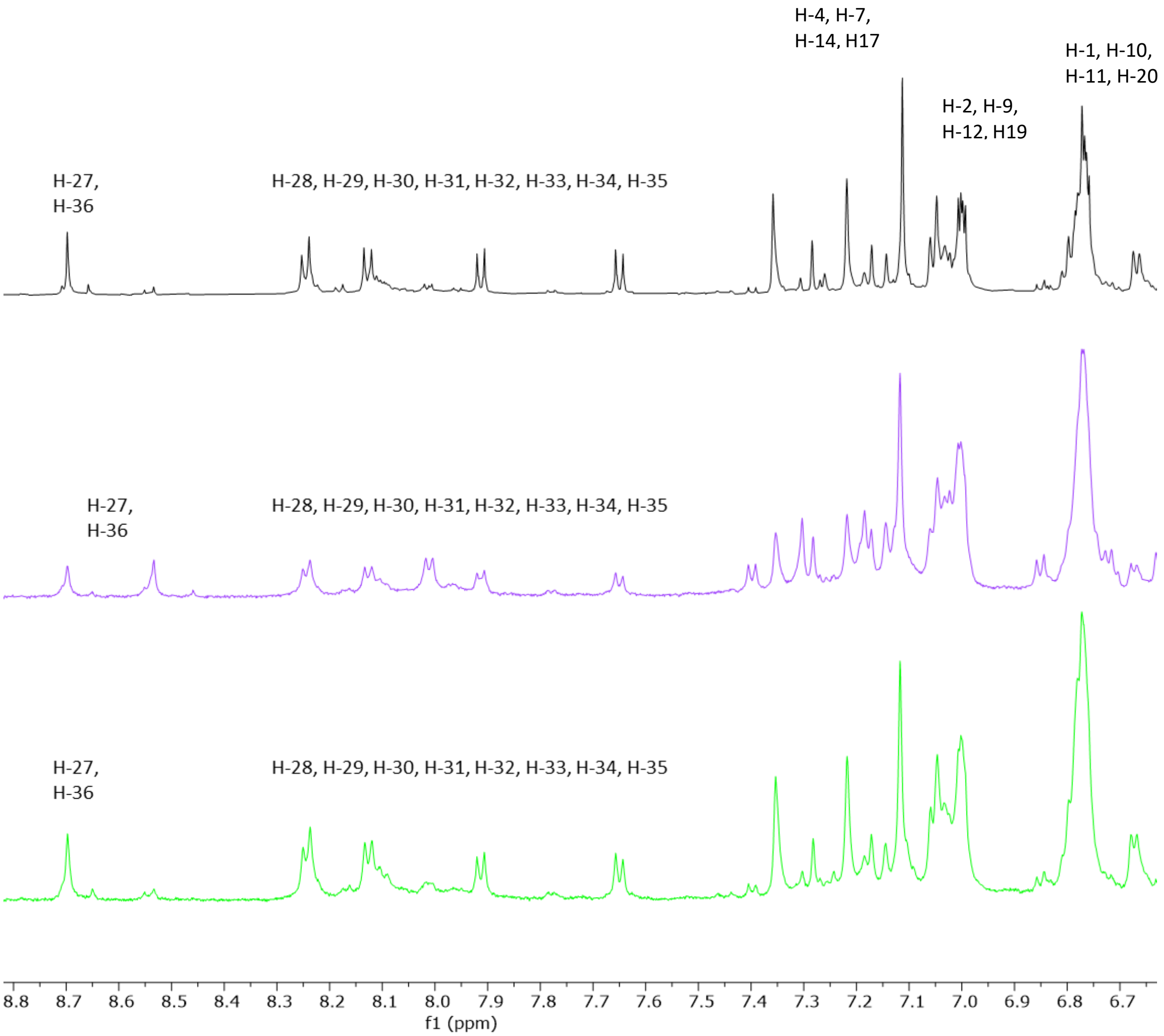

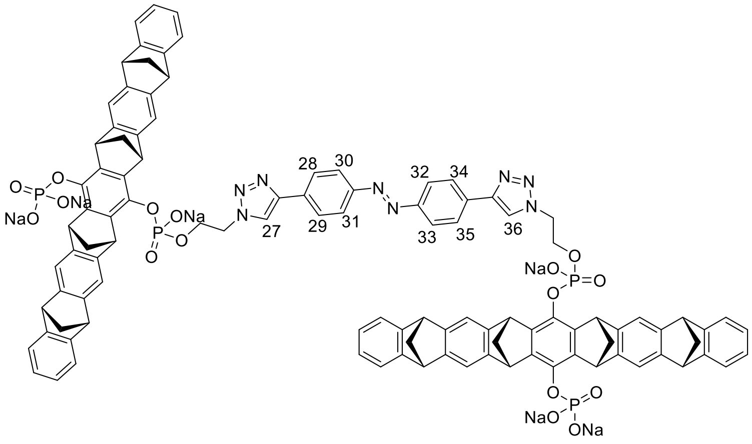

Figure S3f. Full spectra of isolated E/Z isomers of photoswitchable tweezer TWAB1. ^{1}H NMR experiment depicting all changes in signal splitting. Major changes occur in the arylazopyrazole switch and the triazole moiety of **TWAB1**. Synthetic product mixture (black), majority Z isomer obtained by irradiation with 365 nm for 4 min (violet) and majority E isomer obtained from irradiation with 520 nm for 6 min (green).

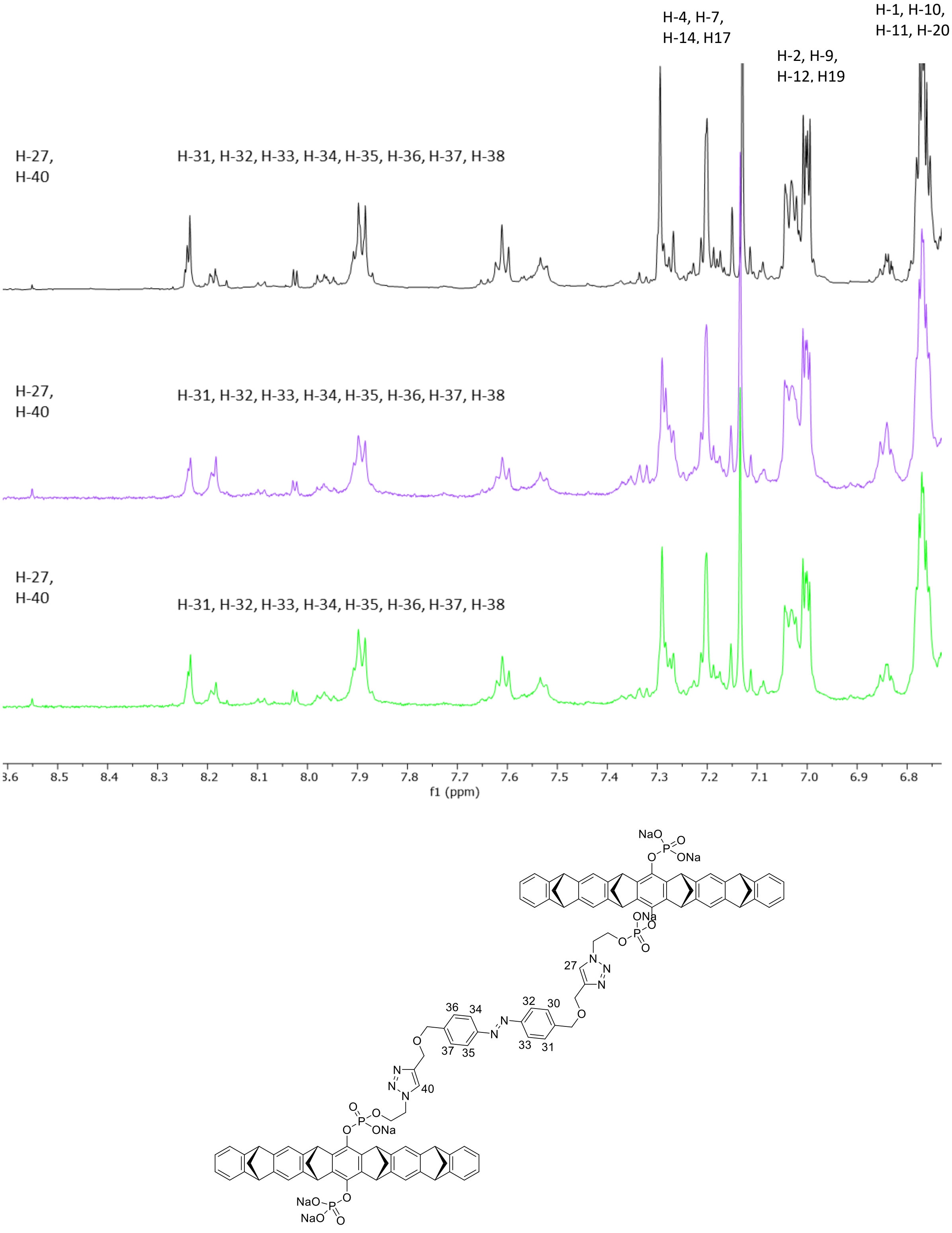

Figure S3g. Full spectra of isolated E/Z isomers of photoswitchable tweezer TWAB2. ^{1}H NMR experiment depicting all changes in signal splitting. Major changes occur in the arylazopyrazole switch and the triazole moiety of **TWAB2**. Synthetic product mixture (black), majority Z isomer obtained by irradiation with 365 nm for 4 min (violet) and majority E isomer obtained from irradiation with 520 nm for 6 min (green).

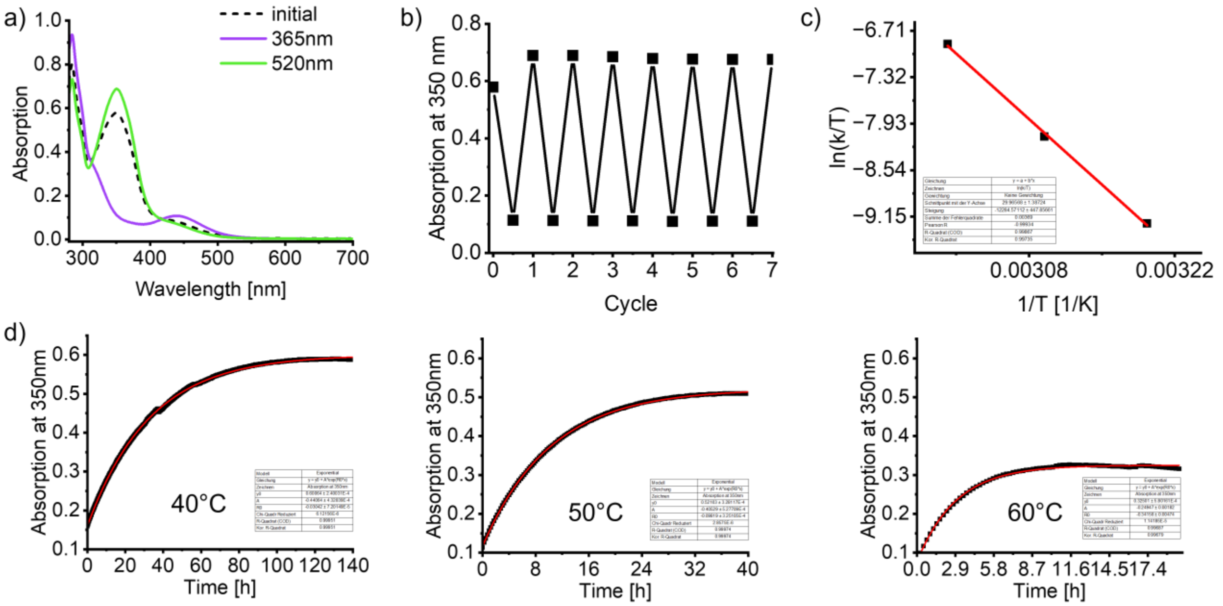

Figure S4a. Photophysical properties of TWAAP0. a) UV-Vis spectra measured after synthesis (black dotted line) and following irradiation with green (520 nm, 5 min, green line) and UV (365 nm, 1 min, violet line) light. b) Repeated photoswitching cycles demonstrating high configurational stability without degradation. c) Eyring plot for the thermal Z-to-E relaxation. d) Thermal Z-to-E relaxation at 40 °C, 50 °C and 60 °C.

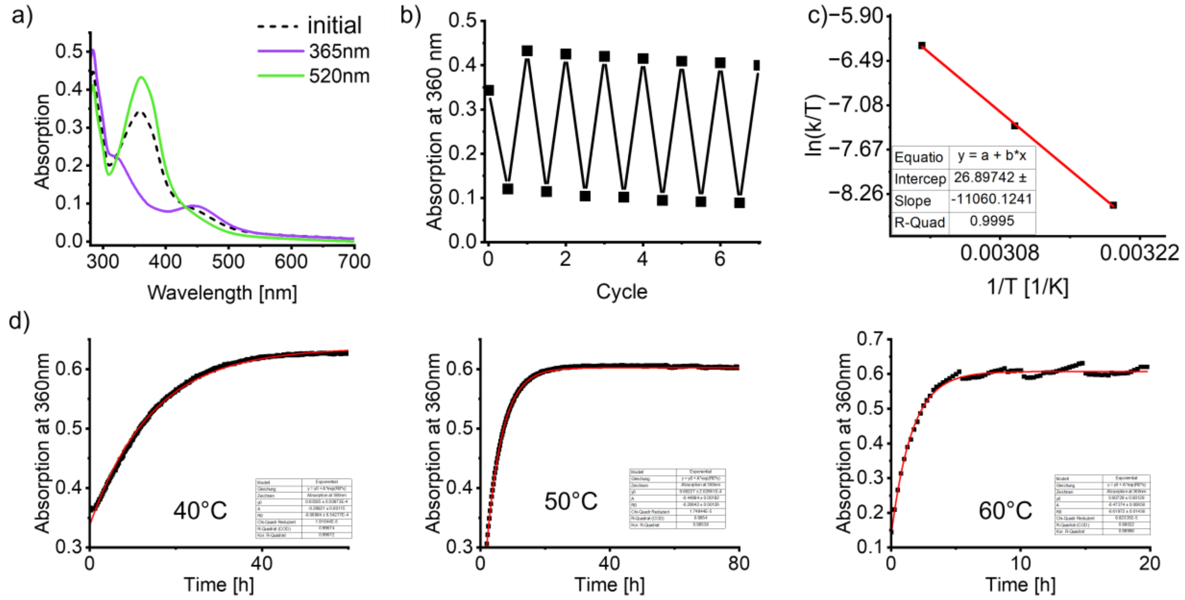

Figure S4b. Photophysical properties of TWAAP1. a) UV-Vis spectra measured after synthesis (black dotted line) and following irradiation with green (520 nm, 5 min, green line) and UV (365 nm, 1 min, violet line) light. b) Repeated photoswitching cycles demonstrating high configurational stability without degradation. c) Eyring plot for the thermal Z-to-E relaxation. d) Thermal Z-to-E relaxation at 40 °C, 50 °C and 60 °C.

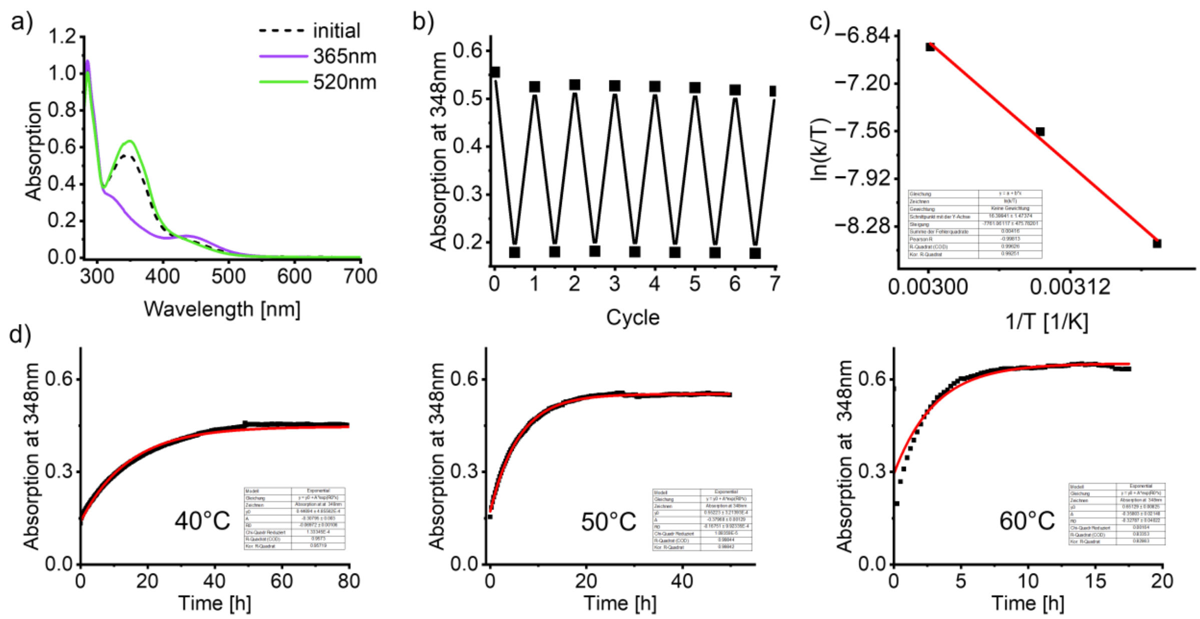

Figure S4c. Photophysical properties of TWAAP2. a) UV-Vis spectra measured after synthesis (black dotted line) and following irradiation with green (520 nm, 5 min, green line) and UV (365 nm, 1 min, violet line) light. b) Repeated photoswitching cycles demonstrating high configurational stability without degradation. c) Eyring plot for the thermal Z-to-E relaxation. d) Thermal Z-to-E relaxation at 40 °C, 50 °C and 60 °C.

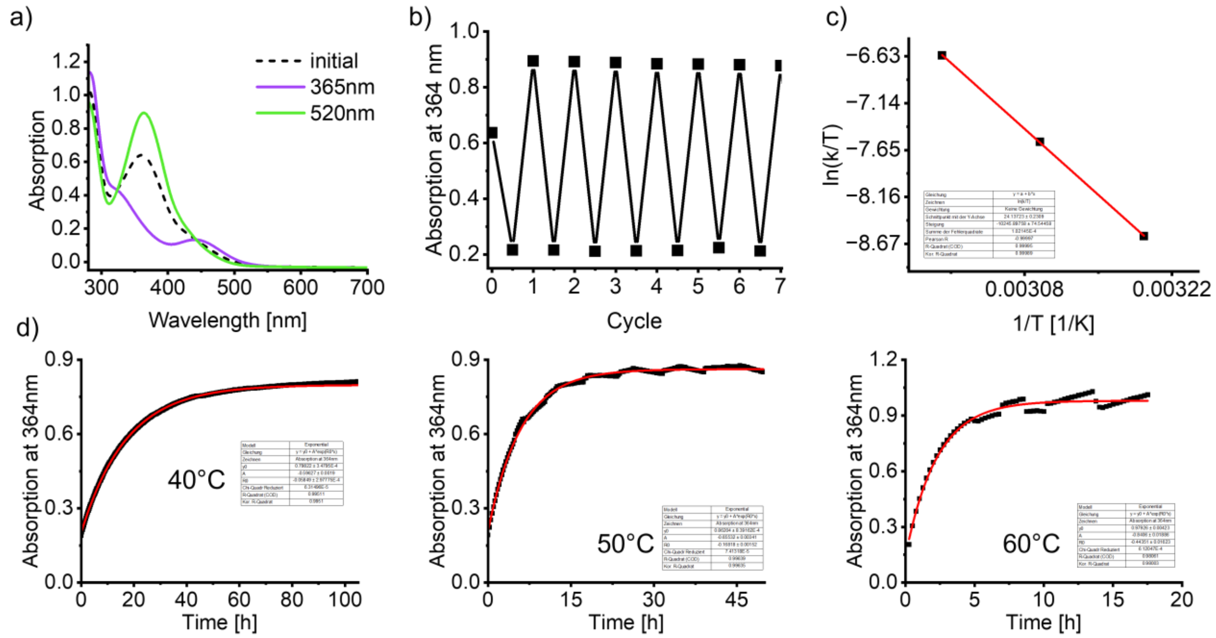

Figure S4d. Photophysical properties of TWAAP3. a) UV-Vis spectra measured after synthesis (black dotted line) and following irradiation with green (520 nm, 5 min, green line) and UV (365 nm, 1 min, violet line) light. b) Repeated photoswitching cycles demonstrating high configurational stability without degradation. c) Eyring plot for the thermal Z-to-E relaxation. d) Thermal Z-to-E relaxation at 40 °C, 50 °C and 60 °C.

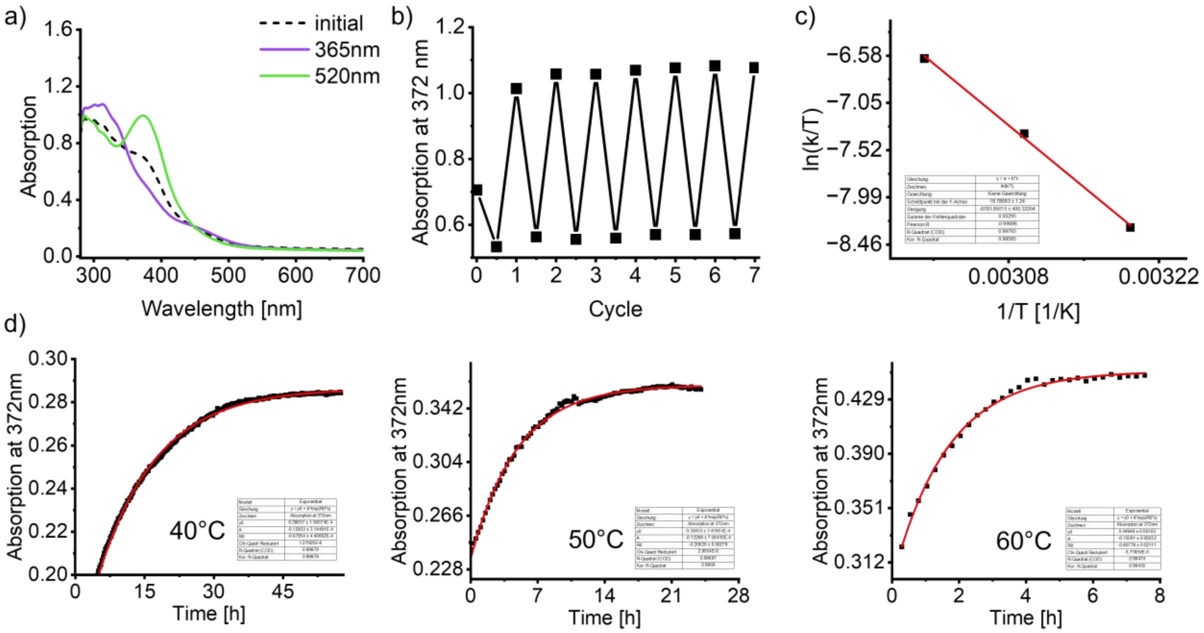

Figure S4e. Photophysical properties of TWAAP4. a) UV-Vis spectra measured after synthesis (black dotted line) and following irradiation with green (520 nm, 5 min, green line) and UV (365 nm, 1 min, violet line) light. b) Repeated photoswitching cycles demonstrating high configurational stability without degradation. c) Eyring plot for the thermal Z-to-E relaxation. d) Thermal Z-to-E relaxation at 40 °C, 50 °C and 60 °C.

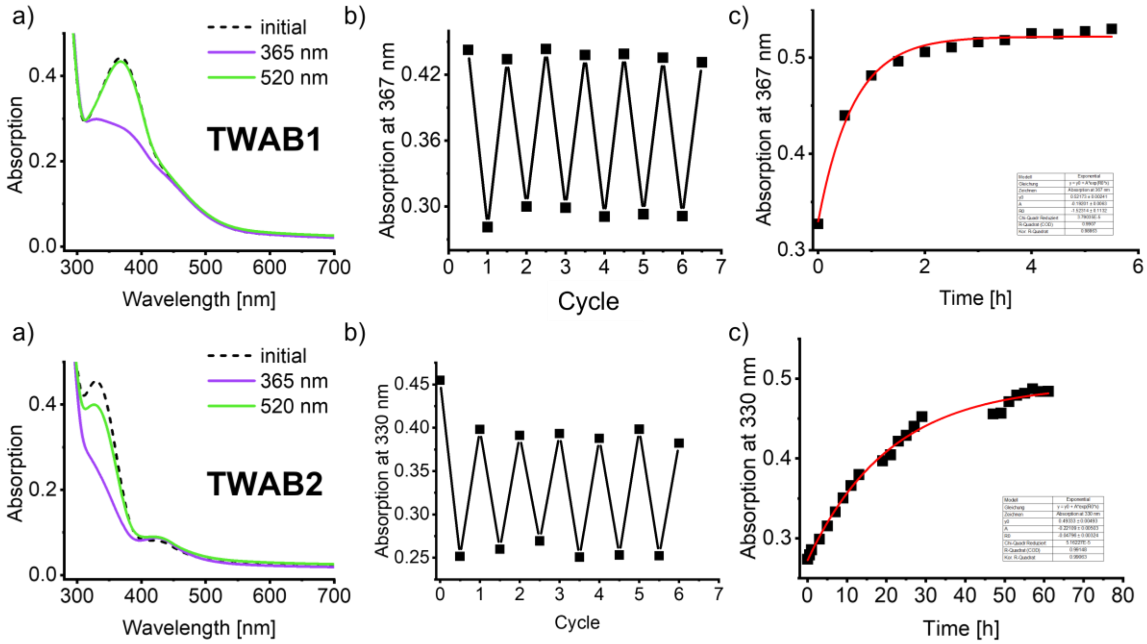

Figure S4f. Photophysical properties of TWAB1-2. a) UV-Vis spectra of the ditopic azobenzene tweezers measured after synthesis (dotted black line) and following irradiation with green (520 nm, 5 min, green line) and UV (365 nm, 1 min, violet line) light. b) Repeated photoswitching cycles demonstrating high configurational stability without degradation. c) Thermal Z-to-E relaxation at 40 °C.

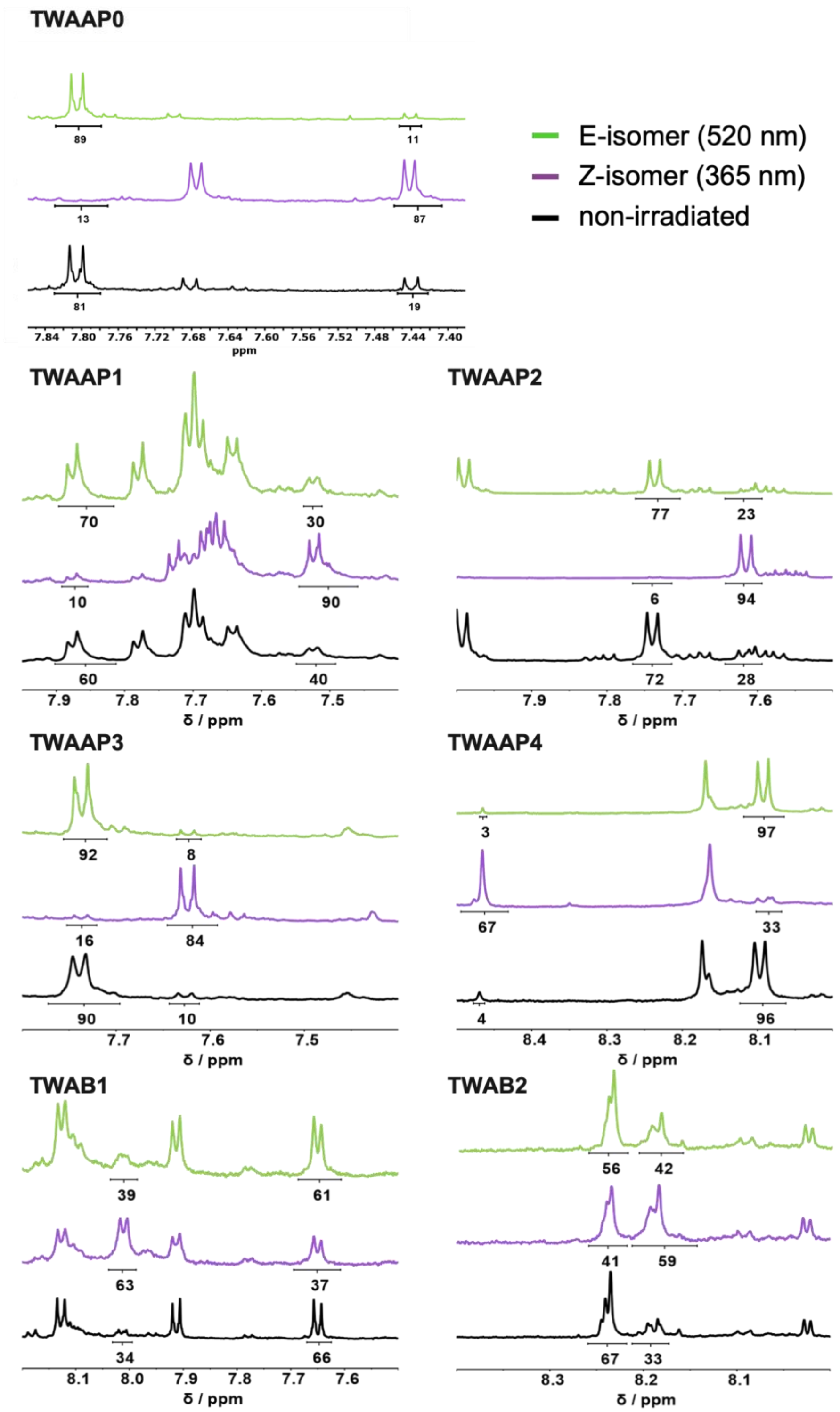

Figure S5. Aromatic regions of the^{1}H-NMR spectrum for the determination of the PSS of TWAAP0-4 and TWAB1-2 (c = 2 mM, 600 MHz, MeOH-d4, 299 K). Black line: initial state, violet: irradiation with UV light (λ = 365 nm, 4 min) (E→Z), green: irradiation with green light (λ = 520 nm, 6 min) (Z→E). Composed with BioRender.com.

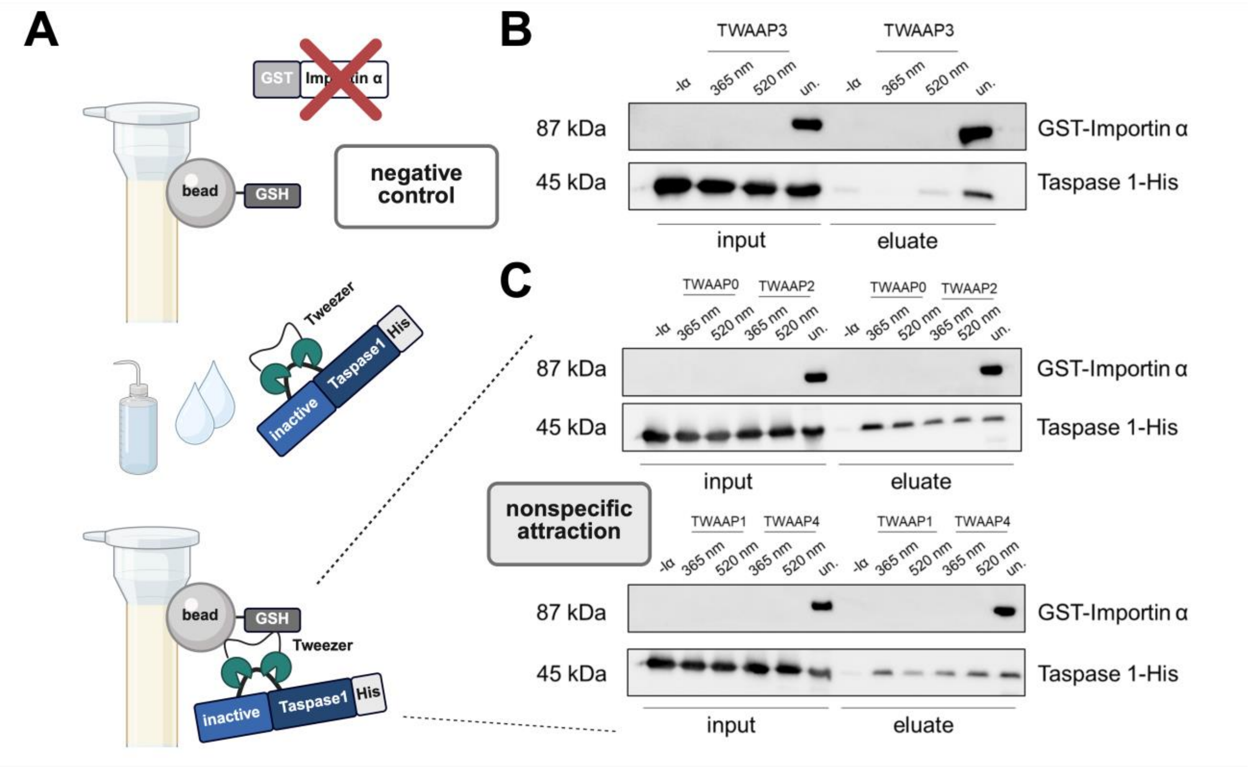

Figure S6. Controls for pull-down assays with TWAAP constructs. A. Schematic assay representation: GST-Importin α was immobilized on a glutathione-coated Sepharose matrix in a spin column. After GST binding, Taspase 1-His pre-incubated with tweezer constructs was added. Unbound proteins were washed away, and eluted proteins were analyzed by SDS-PAGE and Western blot. **B./C.** Pull-down assays using 100 µM of the indicated **TWAAP** constructs pre-irradiated into either the Z- or E-isomeric state. To assess nonspecific attraction of the tweezer constructs to the column beads (**D**), control samples omitted GST-Importin α while retaining 2.2 µM inactive Taspase 1-His as prey. Only **TWAAP3** showed no detectable nonspecific binding (**B**), while **TWAAP0-2** and **TWAAP4** displayed Taspase 1-His in the eluate (**C**). Notably, the nonspecific association of these constructs was apparent from the persistent yellowish coloration of the column, which remained even after washing. (**D**). Composed with BioRender.com.

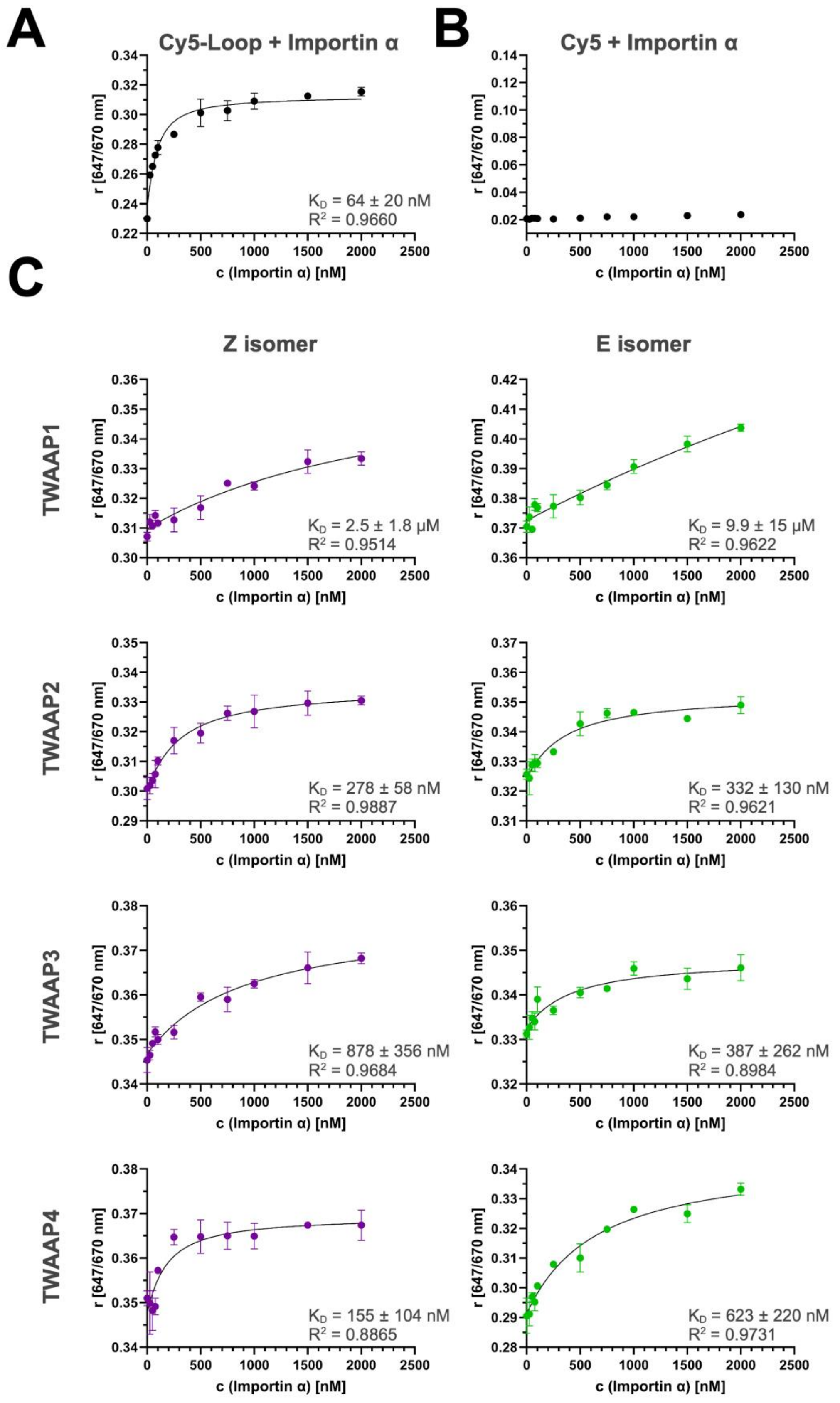
A
Cy5-Loop + Importin α
KD = 64 ± 20 nM
R2 = 0.9660
B
Cy5 + Importin α
C
Z isomer
E isomer
TWAAP1
KD = 2.5 ± 1.8 μM
R2 = 0.9514
KD = 9.9 ± 15 μM
R2 = 0.9622
TWAAP2
KD = 278 ± 58 nM
R2 = 0.9887
KD = 332 ± 130 nM
R2 = 0.9621
TWAAP3
KD = 878 ± 356 nM
R2 = 0.9684
KD = 387 ± 262 nM
R2 = 0.8984
TWAAP4
KD = 155 ± 104 nM
R2 = 0.8865
KD = 623 ± 220 nM
R2 = 0.9731
r [647/670 nm]
c (Importin α) [nM]

Figure S7. Photoswitchable TWAAP constructs interfere with binding of Importin α to the Taspase 1 loop. Fluorescence anisotropy measurements were performed using a Cy5-labeled Taspase 1 loop and switchable tweezers at λ_{ex} = 647 nm/λ_{em} = 670 nm. **A.** Characteristic exponential binding curve of Importin α to the Taspase 1 loop containing the bipartite NLS, with a dissociation constant (K_D) in the low nanomolar range. **B.** Increasing concentrations of Importin α (0-2000 nM) were titrated against 50 nM Cy5 without the coupled peptide as a negative control. **C.** To evaluate the impact on binding, the loop was pre-incubated with 20 µM of the irradiated **TWAAP** constructs before competition with concentrations up to 2000 nM of unlabelled Importin α. Indeed, the presence of the tweezer constructs significantly impaired binding, shifting the K_D to the high nanomolar (**TWAAP1**) or even low micromolar range (**TWAAP2-4**). Data represent the mean ± SD of three independent experiments. Composed with BioRender.com.

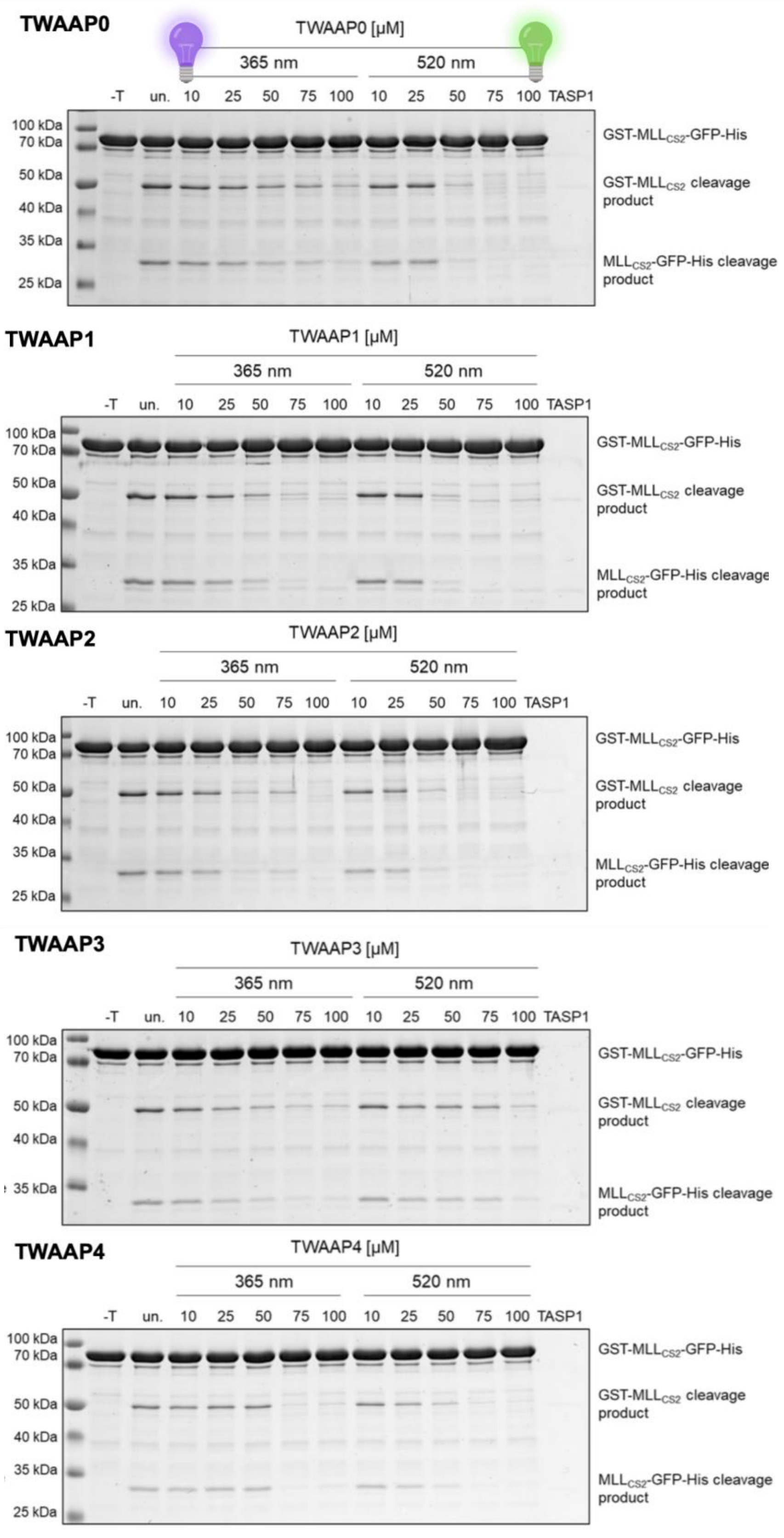

TWAAP0
TWAAP0 [μM]
365 nm
520 nm
-T un. 10 25 50 75 100 10 25 50 75 100 TASP1
100 kDa
70 kDa
50 kDa
40 kDa
35 kDa
25 kDa
GST-MLL$_{CS2}$-GFP-His
GST-MLL$_{CS2}$ cleavage product
MLL$_{CS2}$-GFP-His cleavage product
TWAAP1
TWAAP1 [μM]
365 nm
520 nm
-T un. 10 25 50 75 100 10 25 50 75 100 TASP1
100 kDa
70 kDa
50 kDa
40 kDa
35 kDa
25 kDa
GST-MLL$_{CS2}$-GFP-His
GST-MLL$_{CS2}$ cleavage product
MLL$_{CS2}$-GFP-His cleavage product
TWAAP2
TWAAP2 [μM]
365 nm
520 nm
-T un. 10 25 50 75 100 10 25 50 75 100 TASP1
100 kDa
70 kDa
50 kDa
40 kDa
35 kDa
25 kDa
GST-MLL$_{CS2}$-GFP-His
GST-MLL$_{CS2}$ cleavage product
MLL$_{CS2}$-GFP-His cleavage product
TWAAP3
TWAAP3 [μM]
365 nm
520 nm
-T un. 10 25 50 75 100 10 25 50 75 100 TASP1
100 kDa
70 kDa
50 kDa
40 kDa
35 kDa
GST-MLL$_{CS2}$-GFP-His
GST-MLL$_{CS2}$ cleavage product
MLL$_{CS2}$-GFP-His cleavage product
TWAAP4
TWAAP4 [μM]
365 nm
520 nm
-T un. 10 25 50 75 100 10 25 50 75 100 TASP1
100 kDa
70 kDa
50 kDa
40 kDa
35 kDa
25 kDa
GST-MLL$_{CS2}$-GFP-His
GST-MLL$_{CS2}$ cleavage product
MLL$_{CS2}$-GFP-His cleavage product

Figure S8. Photoswitchable TWAAP constructs interfere with Taspase 1's proteolytic activity. Cleavage assay utilizing the cleavage site CS2 from the natural substrate MLL (GST-MLL$_{2700-2850}$-GFP-His, 70 kDa) and active wildtype Taspase 1. Inhibition of proteolysis was evaluated by the relative amount of uncleaved in relation to cleaved substrate (50 kDa and 20 kDa, respectively). Briefly, 5 µM recombinant substrate were incubated with 200 nM wildtype Taspase 1-His and increasing concentrations (10 - 100 µM) of indicated **TWAAP** irradiated with UV (365 nm, left gel panels) or green light (520 nm, right gel panels). Solvent-treated samples (referred to as untreated, un.) served as reference, and as additional controls, Taspase 1 (-T) was omitted or only Taspase 1 (TASP1) was applied. Representative images of three independent experiments are shown. Composed with BioRender.com.

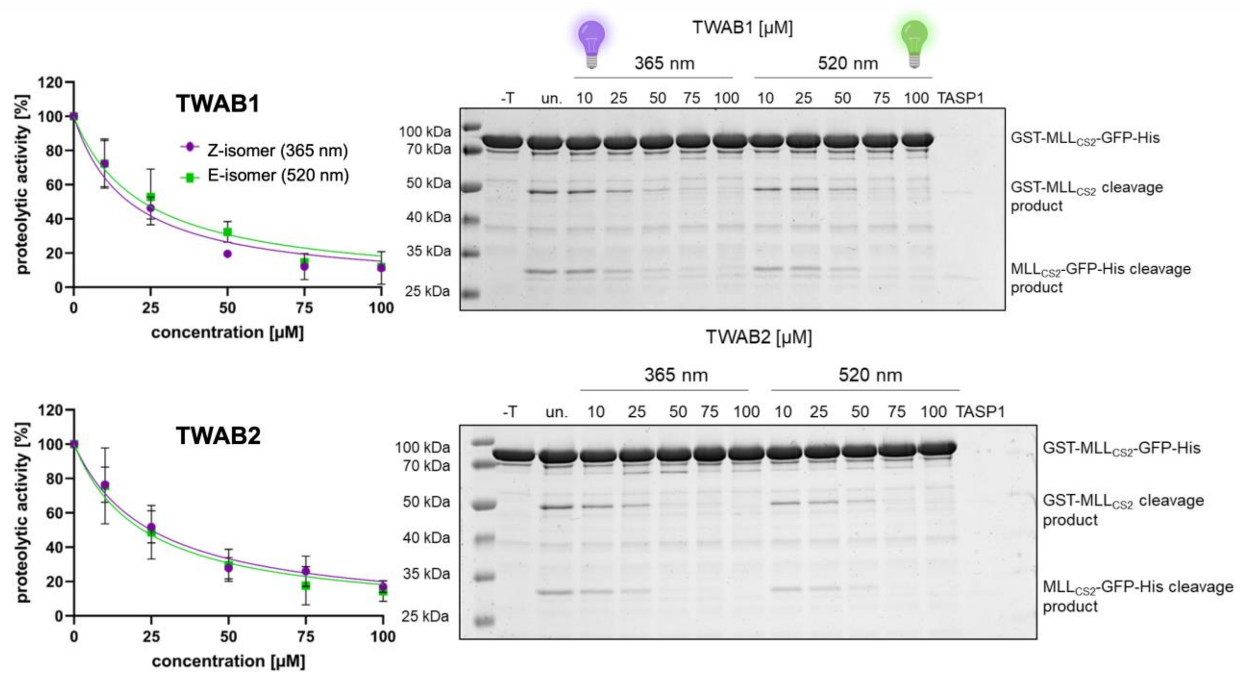

Figure S9. Photoswitchable TWAAB constructs interfere with Taspase 1's proteolytic activity. Cleavage assay utilizing the cleavage site CS2 from the natural substrate MLL (GST-MLL$_{2700-2850}$-GFP-His, 70 kDa) and active wildtype Taspase 1. Inhibition of proteolysis was evaluated by the relative amount of uncleaved in relation to cleaved substrate (50 kDa and 20 kDa, respectively). Briefly, 5 µM recombinant substrate were incubated with 200 nM wildtype Taspase 1-His and increasing concentrations (10 - 100 µM) of indicated **TWAB** irradiated with UV (365 nm, magenta/left gel panels) or green light (520 nm, green/right gel panels). Solvent-treated samples (referred to as untreated, un.) served as reference, and as additional controls, Taspase 1 (-T) was omitted or only Taspase 1 (TASP1) was applied. Proteolytic activity after solvent treatment was set to 100 %, and samples treated with **TWAB** constructs were referenced to this value to allow densitometric quantification of the colorimetric cleavage assays (left panels). Data are shown as the mean ± SD from three independent experiments. Representative images of three independent experiments are shown (right panels). Composed with BioRender.com.

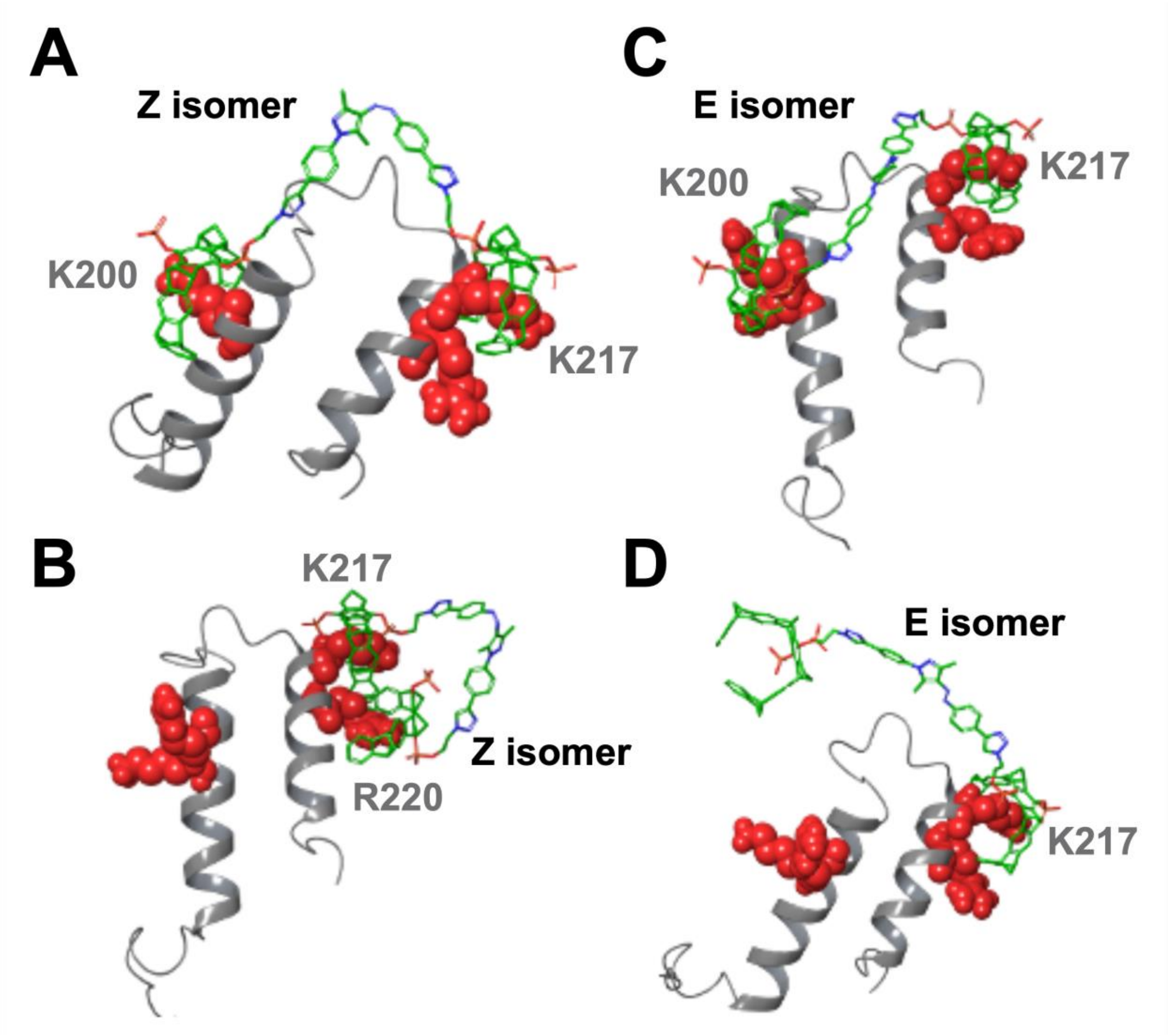

Figure S10. Binding modes of TWAAP1 to the Taspase 1 loop. A. Z isomer bridging both α-helices between K200 and K217. **B.** Z isomer bridging K217 and R220 within the same α-helix. **C.** E isomer bridging both α-helices between K200 and K217. **D.** E isomer on K217 without bridging ability. Composed with BioRender.com.

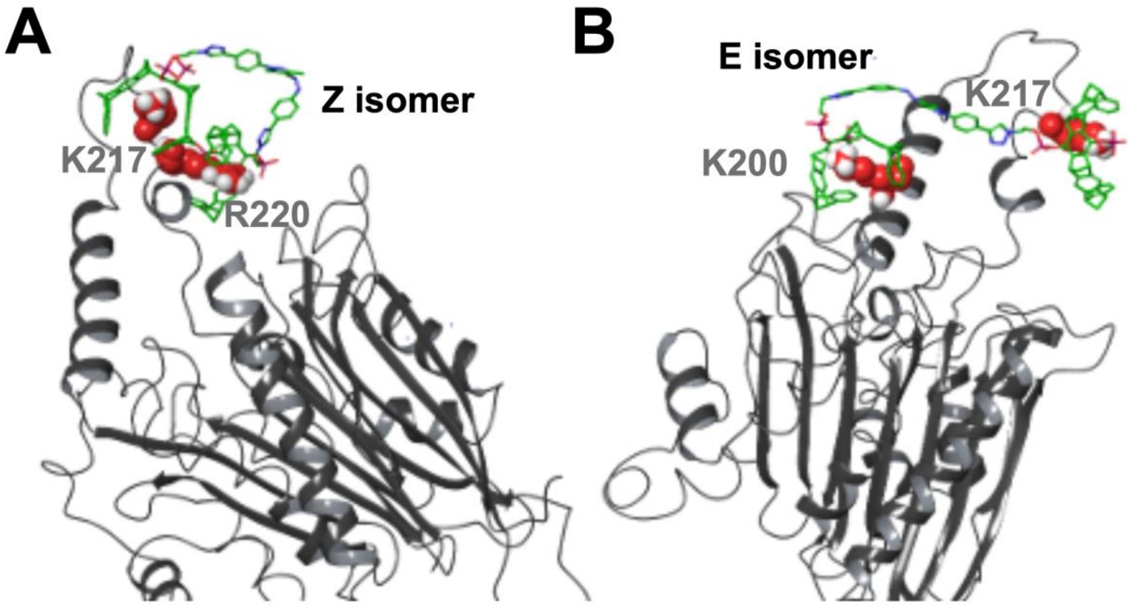

Figure S11. MD simulation of the complex between TWAAP1 and full-length Taspase 1. A. Z isomer bridging the same α-helix between K217 and R220. **B.** E isomer bridging both α-helices on K200 and K217. Composed with BioRender.com.

Supplementary Tables

Supplementary Table S1. Photostationary states ($PSS_{E\rightarrow Z}$ and $PSS_{Z\rightarrow E}$) and half-life of Z isomers of **TWAAP0-4** and **TWAB1-2** at 20 °C ($(t_{1/2})_{Z,\ 20°C}$) as determined by ^{1}H NMR and UV/vis spectroscopy, respectively. n.d., not determinable.

**TW** construct	$PSS_{E\rightarrow Z}$ [%]	$PSS_{Z\rightarrow E}$ [%]	$(t_{1/2})_{Z,\ 20°C}$ [h]
**TWAAP0**	87	89	361
**TWAAP1**	90	70	119
**TWAAP2**	94	77	91
**TWAAP3**	84	92	117
**TWAAP4**	67	97	63
**TWAB1**	63	61	n.d.
**TWAB2**	59	56	n.d.

Supplementary Table S2. K_D values of the E or Z enriched isomers as determined by SPR (see **Fig. 3**).

**TW** construct	K_D (Z) [µM]	K_D (E) [µM]
**TWAAP0**	2.87 ± 0.09	0.71 ± 0.02
**TWAAP2**	4.28 ± 0.36	5.67 ± 0.58
**TWAAP3**	2.67 ± 0.16	4.05 ± 3.17
**TWAAP4**	1.96 ± 0.57	2.90 ± 0.63
**TWAB1**	9.20 ± 2.27	9.32 ± 1.56

Note: Compound TWAAP1 was not included in Figure 3 and Table S2 because SPR measurements were not feasible due to technical limitations encountered with this compound.

Supplementary Table S3. Kinetic parameters of photoswitchable tweezer constructs as determined by SPR (see **Fig. 3**). Values were derived from fitted sensorgrams with a 1:1 two state binding model from n = 2 measurements for the indicated constructs. Analysis was performed using the Trace Drawer software 1.9.2 (TraceDrawer Software, Sweden).

**TW** construct	Isomer	k_a [$M^{-1}s^{-1}$]	k_d [s^{-1}]	K_D [µM]	Chi^2	U-value [%]
**TWAAP0**	Z	1.04e3±2.05e1	7.43e-4±4.98-6	0.71±0.02	6.01	2.30
	E	1.12e3±3.40e1	3.22e-3±7.17e-7	2.87±0.09	9.56	1.30
**TWAAP2**	Z	7.50e2±7.62e1	4.26e-3±1.03e-6	5.67±0.58	253.60	1.60
	E	1.17e3±9.64e1	5.01e-3±9.88e-7	4.28±0.36	140.16	1.30
**TWAAP3**	Z	3.81e3±2.09e3	1.53e-2±2.50e-5	4.05±3.17	9.92	3.40
	E	9.57e2±5.61e1	2.56e-3±4.04e-6	2.67±0.16	31.77	1.70
**TWAAP4**	Z	3.34e3±6.88e2	9.67e-3±4.04e-6	2.90±0.63	1.77	3.00
	E	4.69e3±1.26e3	9.18e-3±4.22e-6	1.96±0.57	1.78	3.80
**TWAB1**	Z	6.79e2±4.05e2	6.33e-4±3.06e-4	9.32±1.56	5.23	9.4
	E	5.96e2±1.16e2	5.48e-4±2.40e-5	9.20±2.27	3.51	6.9

Supplementary Table S4. Summarized binding and activity data of photoswitchable tweezer constructs (Z and E isomers) as evaluated by the indicated experimental methods. The distances of the tweezer moieties were determined using Pymol (**see Table 1**). Determined K_D values from the SPR equilibrium response curve of the full-length Taspase 1 protein fitted by using a 1:1 two state binding model and standard deviation from n = 2 measurements (see **Fig. 3**). Determined percentage of the PPI of Importin α with full-length Taspase 1 in the recombinant pull-down assay and standard deviation from n = 3 independent experiments (see **Fig. 7**). Determined K_D values from fluorescence titrations of the Cy5-labelled loop peptide fitted to a 1:1 binding model and standard deviation from n = 3 independent experiments (see **Fig. 4**). Determined K_D values from fluorescence polarization competition assays utilizing the Cy5-labelled loop peptide and excess Importin α fitted to a 1:1 binding model and standard deviation from n = 3 independent experiments (see **Fig. S7**). Determined IC_{50} values for inhibition of proteolytic activity of full-length Taspase 1 in the recombinant cleavage assay (see **Fig. 5** and **Fig. 6**) referenced to solvent treatment and standard deviation from n = 3 independent experiments. n.d., not done. *p ≤ 0.05.

**TW** construct	Iso-mer	Distance [Å]	K_D **TW** [µM] (SPR, full-length protein; ligand conc. 0.06 - 25 µM)	PPI **TW** [%] (pull-down, full-length protein; ligand conc. 25 µM)	K_D **TW** [µM] (fluorescence titration, loop; ligand conc. 0.1 - 10 µM)	K_D Imp α [µM] (fluorescence polarization, loop:Importin α competition; ligand conc. 20 µM)*	IC_{50} **TW** [µM] (recombinant cleavage, full-length protein; ligand conc. 10 - 100 µM)
**TWAAP0**	Z	18	2.9 ± 0.1	n.d.	0.3 ± 0.1	n.d.	44.6 ± 8.2
	E	30	0.7 ± 0.0	n.d.	0.2 ± 0.1	n.d.	25.9 ± 4.4
**TWAAP1**	Z	20	n.d.	n.d.	0.7 ± 0.3	2.5 ± 1.8	48.5 ± 6.0
	E	30	n.d.	n.d.	0.5 ± 0.4	9.9 ± 15	28.2 ± 3.2
**TWAAP2**	Z	13	4.3 ± 0.1	n.d.	0.3 ± 0.1	0.3 ± 0.1	25.4 ± 3.8
	E	22	5.7 ± 0.6	n.d.	0.3 ± 0.2	0.3 ± 0.1	17.5 ± 2.3
**TWAAP3**	Z	15	2.7 ± 0.2	60 ± 10	0.6 ± 0.3	0.9 ± 0.4	41.9 ± 5.3
	E	32	4.1 ± 3.2	90 ± 25	0.4 ± 0.2	0.4 ± 0.3	49.4 ± 3.9
**TWAAP4**	Z	13	2.0 ± 0.6	n.d.	4.9 ± 3.1	0.2 ± 0.1	37.9 ± 8.4
	E	38	2.9 ± 0.6	n.d.	0.8 ± 0.4	0.6 ± 0.2	24.7 ± 2.7
**TWAB1**	Z	16	9.2 ± 2.3	n.d.	n.d.	n.d.	17.9 ± 1.9
	E	28	9.3 ± 1.6	n.d.	n.d.	n.d.	22.3 ± 2.4
**TWAB2**	Z	12	n.d.	n.d.	n.d.	n.d.	24.9 ± 2.2
	E	17	n.d.	n.d.	n.d.	n.d.	22.3 ± 3.3

*) Please note that the fluorescence polarization assay assesses the competition between **TWAAP** constructs and Importin α for binding to the Cy5-labeled Taspase 1 loop (K_D = 64 ± 20 nM). A higher K_D indicates stronger **TWAAP** binding, as it more effectively disrupts complex formation.

Supplementary Tables S5-S12 present all relevant amino acid distances within the loop and surrounding the active site of Taspase 1. These distances can be correlated with the distances between the two tweezers in the E- and Z-isomers, as shown in Fig. S2.

Supplementary Table S5. Relevant distances between the lysine / arginine residues within the first basic NLS cluster (^{197}KRNKRK^{202}).

Amino acid I	Amino acid II	Distance [Å]
K197	R198	11.3
K197	R201	13.7
R201	K202	12.3

Supplementary Table S6. Relevant distances between the lysine / arginine residues within the second basic NLS cluster (^{217}KKRR^{220}).

Amino acid I	Amino acid II	Distance [Å]
K217	K218	13.8
K217	R219	11.9
K218	R219	11.6
R219	R220	14.0

Supplementary Table S7. Relevant distances between the lysine / arginine residues of both basic NLS clusters (^{197}KRNKRK^{202} and ^{217}KKRR^{220}).

**Amino acid I**	**Amino acid II**	**Distance [Å]**
K217	K197	33.6
K217	R198	27.7
K217	K200	28.6
K217	R201	27.0
K218	K197	27.5
K218	R198	25.2
R219	K197	28.6
R219	R198	26.8 (sterically not accessible)
R220	K197	26.1

Supplementary Table S8. Relevant distances between the lysine / arginine residues of the basic NLS clusters (^{197}KRNKRK^{202} or ^{217}KKRR^{220}) and Lys57.

**Amino acid I**	**Amino acid II**	**Distance [Å]**
K57	R198	10.0
K57	K202	13.5
K57	K217	27.1
K57	K218	29.4
K57	R219	27.5

Supplementary Table S9. All distances between the lysine / arginine residues within the first basic NLS cluster (^{197}KRNKRK^{202}).

Amino acid I	Amino acid II	Distance [Å]
K197	R198	11.3
K197	K200	10.1
K197	R201	13.7
K197	K202	15.1
R198	K200	16.6
R198	R201	15.5
R198	K202	7.8
K200	R201	10.0
K200	K202	15.4
R201	K202	12.3

Supplementary Table S10. All distances between the lysine / arginine residues within the second basic NLS cluster (^{217}KKRR^{220}).

Amino acid I	Amino acid II	Distance [Å]
K217	K218	13.8
K217	R219	11.9
K217	R220	9.4
K218	R219	11.6
K218	R220	15.6
R219	R220	14

Supplementary Table S11. All distances between the lysine / arginine residues of both basic NLS clusters (^{197}KRNKRK^{202} and ^{217}KKRR^{220}).

Amino acid I	Amino acid II	Distance [Å]
K217	K197	33.6
K217	R198	27.7
K217	K200	28.6
K217	R201	27.0
K217	K202	20.7
K218	K197	27.5
K218	R198	25.2
K218	K200	20.3
K218	R201	17.0
K218	K202	17.8
R219	K197	28.6
R219	R198	26.8
R219	K200	21.3
R219	R201	23.1
R219	K202	20.5
R220	K197	26.1
R220	R198	18.9
R220	K200	23.5
R220	R201	22.2
R220	K202	12.8

Supplementary Table S12. All distances between the lysine / arginine residues of the basic NLS clusters (^{197}KRNKRK^{202} or ^{217}KKRR^{220}) and Lys57.

**Amino acid I**	**Amino acid II**	**Distance [Å]**
K57	K217	27.1
K57	K218	29.4
K57	R219	27.5
K57	R220	17.8
K57	K197	18.6
K57	R198	10.0
K57	K200	23.1
K57	R201	24.2
K57	K202	13.5
